# In-hospital initiation of angiotensin receptor–neprilysin inhibition in acute heart failure: the PREMIER trial

**DOI:** 10.1093/eurheartj/ehae561

**Published:** 2024-08-31

**Authors:** Atsushi Tanaka, Keisuke Kida, Yuya Matsue, Takumi Imai, Satoru Suwa, Isao Taguchi, Itaru Hisauchi, Hiroki Teragawa, Yoshiyuki Yazaki, Masao Moroi, Koichi Ohashi, Daisuke Nagatomo, Toru Kubota, Takeshi Ijichi, Yuji Ikari, Keisuke Yonezu, Naohiko Takahashi, Shigeru Toyoda, Tsutomu Toshida, Hiroshi Suzuki, Tohru Minamino, Kazutaka Nogi, Kazuki Shiina, Yu Horiuchi, Kengo Tanabe, Daisuke Hachinohe, Shunsuke Kiuchi, Kenya Kusunose, Michio Shimabukuro, Koichi Node

**Affiliations:** Department of Cardiovascular Medicine, Saga University, 5-1-1 Nabeshima, Saga 849-8501, Japan; Department of Pharmacology, St. Marianna University School of Medicine, Kawasaki, Japan; Department of Cardiovascular Biology and Medicine, Juntendo University Graduate School of Medicine, Tokyo, Japan; Clinical Research Division, Organization for Clinical Medicine Promotion, Tokyo, Japan; Clinical and Translational Research Center, Kobe University Hospital, Kobe, Japan; Department of Cardiology, Juntendo University Shizuoka Hospital, Shizuoka, Japan; Department of Cardiology, Dokkyo Medical University Saitama Medical Center, Koshigaya, Japan; Department of Cardiology, Dokkyo Medical University Saitama Medical Center, Koshigaya, Japan; Department of Cardiovascular Medicine, JR Hiroshima Hospital, Hiroshima, Japan; Division of Cardiovascular Medicine, Toho University Ohashi Medical Center, Tokyo, Japan; Division of Cardiovascular Medicine, Toho University Ohashi Medical Center, Tokyo, Japan; Department of Cardiology, Tokyo Metropolitan Bokutoh Hospital, Tokyo, Japan; Division of Cardiology, Cardiovascular and Aortic Center, Saiseikai Fukuoka General Hospital, Fukuoka, Japan; Division of Cardiology, Cardiovascular and Aortic Center, Saiseikai Fukuoka General Hospital, Fukuoka, Japan; Department of Cardiology, Tokai University, Isehara, Japan; Department of Cardiology, Tokai University, Isehara, Japan; Department of Cardiology and Clinical Examination, Faculty of Medicine, Oita University, Yufu, Japan; Department of Cardiology and Clinical Examination, Faculty of Medicine, Oita University, Yufu, Japan; Department of Cardiovascular Medicine, Dokkyo Medical University, Mibu, Japan; Division of Cardiology, Department of Internal Medicine, Showa University Fujigaoka Hospital, Yokohama, Japan; Division of Cardiology, Department of Internal Medicine, Showa University Fujigaoka Hospital, Yokohama, Japan; Department of Cardiovascular Biology and Medicine, Juntendo University Graduate School of Medicine, Tokyo, Japan; Department of Cardiovascular Medicine, Nara Medical University, Kashihara, Japan; Department of Cardiology, Tokyo Medical University, Tokyo, Japan; Division of Cardiology, Mitsui Memorial Hospital, Tokyo, Japan; Division of Cardiology, Mitsui Memorial Hospital, Tokyo, Japan; Department of Cardiology, Sapporo Heart Center, Sapporo Cardio Vascular Clinic, Sapporo, Japan; Department of Cardiovascular Medicine, Toho University Faculty of Medicine, Tokyo, Japan; Department of Cardiovascular Medicine, Nephrology, and Neurology, Graduate School of Medicine, University of the Ryukyus, Okinawa, Japan; Department of Diabetes, Endocrinology, and Metabolism, Fukushima Medical University School of Medicine, Fukushima, Japan; Department of Cardiovascular Medicine, Saga University, 5-1-1 Nabeshima, Saga 849-8501, Japan

**Keywords:** Sacubitril/valsartan, Acute heart failure, N-terminal pro-B-type natriuretic peptide

## Abstract

**Background and Aims:**

The efficacy and safety of early sacubitril/valsartan (Sac/Val) initiation after acute heart failure (AHF) has not been demonstrated outside North America. The present study aimed to evaluate the effect of in-hospital Sac/Val therapy initiation after an AHF episode on N-terminal pro-B-type natriuretic peptide (NT-proBNP) level in Japanese patients.

**Methods:**

This was an investigator-initiated, multicentre, prospective, randomized, open-label, blinded-endpoint pragmatic trial. After haemodynamic stabilization within 7 days after hospitalization, eligible inpatients were allocated to switch from angiotensin-converting enzyme inhibitor or angiotensin receptor blocker to Sac/Val (Sac/Val group) or to continue angiotensin-converting enzyme inhibitor or angiotensin receptor blocker (control group). The primary efficacy endpoint was the 8-week proportional change in geometric means of NT-proBNP levels.

**Results:**

A total of 400 patients were equally randomized, and 376 (median age 75 years, 31.9% women, *de novo* heart failure rate 55.6%, and median left ventricular ejection fraction 37%) were analysed. The per cent changes in NT-proBNP level geometric means at Weeks 4/8 were −35%/−45% (Sac/Val group) and −18%/−32% (control group), and their group ratio (Sac/Val vs. control) was 0.80 (95% confidence interval 0.68–0.94; *P* = .008) at Week 4 and 0.81 (95% confidence interval 0.68–0.95; *P* = .012) at Week 8, respectively. In the pre-specified subgroup analyses, the effects of Sac/Val were confined to patients with a left ventricular ejection fraction < 40% and were more evident in those in sinus rhythm and taking mineralocorticoid receptor antagonists. No adverse safety signal was evident.

**Conclusions:**

In-hospital Sac/Val therapy initiation in addition to contemporary recommended therapy triggered a greater NT-proBNP level reduction in Japanese patients hospitalized for AHF. These findings may expand the evidence on Sac/Val therapy in this clinical situation outside North America.

**Clinical Trial Registration:**

ClinicalTrial.gov (NCT05164653) and Japan Registry of Clinical Trials (jRCTs021210046).

## Structured Graphical Abstract

**Structured Graphical Abstract:**
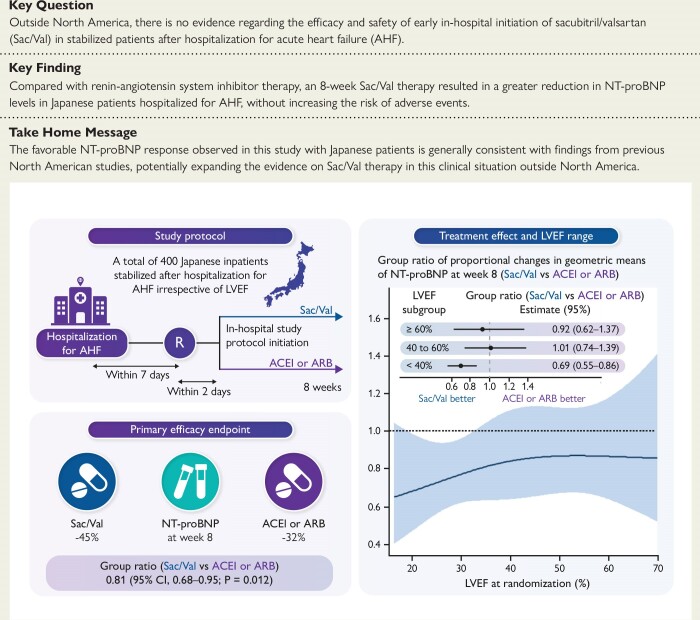
N-terminal pro-B-type natriuretic peptide response to early in-hospital initiation of angiotensin receptor–neprilysin inhibition (sacubitril/valsartan) in Japanese patients stabilized after hospitalization for acute heart failure. ACEI, angiotensin-converting enzyme inhibitor; AHF, acute heart failure; ARB, angiotensin receptor blocker; CI, confidence interval; LVEF, left ventricular ejection fraction; NT-proBNP, N-terminal pro-B-type natriuretic peptide; Sac/Val, sacubitril/valsartan.

**See the editorial comment for this article ‘Implementing medical therapy during worsening heart failure’, by A.S. Bhatt and M. Vaduganathan, https://doi.org/10.1093/eurheartj/ehae566.**

## Introduction

Acute heart failure (AHF) remains a global clinical challenge that poses huge socio-economic and medical burdens.^1^ Early initiation and accelerated optimization of guideline-directed medical therapy (GDMT) are recommended to improve post-AHF outcomes.^2–5^

Two landmark studies, the Comparison of Sacubitril-Valsartan vs. Enalapril on Effect of NT-proBNP in Patients Stabilized from an Acute Heart Failure Episode (PIONEER-HF) and the Prospective Comparison of ARNI and ARB Given Following Stabilization in Decompensated HFpEF (PARAGLIDE-HF) demonstrated that early initiation of sacubitril/valsartan (Sac/Val) led to a greater reduction in N-terminal pro-B-type natriuretic peptide (NT-proBNP) levels in patients stabilized after AHF, especially in those with a left ventricular ejection fraction (LVEF) below normal.^6–8^

The above-mentioned studies were conducted in North America and included few Asian patients. Generally, there are substantial differences between the USA and Japan in terms of medical systems, clinical practice, and outcomes of patients with AHF^9–11^; hence, it is clinically relevant to examine whether the current evidence on Sac/Val therapy in patients with AHF can expand outside North America. Therefore, the Program Angiotensin-Neprilysin Inhibition in Admitted Patients with Worsening Heart Failure (PREMIER) study was conducted to assess the efficacy and safety of in-hospital Sac/Val therapy initiation after an AHF event in the Japanese clinical setting.

## Methods

### Study design overview

The PREMIER study was an investigator-initiated, multicentre, prospective, randomized-controlled, open-label, blinded-endpoint trial. Initially, patients were screened for eligibility based on their medical records; subsequently, all potential participants received adequate explanations about the study plan and provided written informed consent. The participants were enrolled from December 2021 to June 2023 at 44 sites in Japan (see Supplementary data online, *Study sites list*). The full study protocol is presented in Supplementary data online, *Study protocol*.

### Study population

A complete list of inclusion and exclusion criteria is provided in Supplementary data online, *Inclusion* and *exclusion criteria*. In brief, the study included adult patients who were hospitalized owing to a primary diagnosis of AHF with signs and symptoms of heart failure (HF) [New York Heart Association (NYHA) functional Classes II–IV, irrespective of LVEF status and acute *de novo* or decompensated chronic HF] and had an NT-proBNP level ≥ 1200 pg/mL or a B-type natriuretic peptide level ≥ 300 pg/mL from 48 h prior to the index hospitalization to the time of eligibility assessment. Participants were also required to be on renin–angiotensin system inhibitors, namely angiotensin-converting enzyme inhibitor or angiotensin receptor blocker (ACEI/ARB) and to be haemodynamically stable according to the following definitions: (i) systolic blood pressure (BP) ≥ 100 mm Hg, (ii) no increase in intravenous diuretic agents within the last 6 h before randomization, and (iii) no intravenous administration of vasodilators or inotropic agents. The main exclusion criteria were (i) severe renal, hepatic, infectious, or pulmonary disease; (ii) hyperkalaemia (≥5.3 mEq/L); (iii) unstable haemodynamic condition, including cardiogenic shock and the use of mechanical support; (iv) a recent history of acute coronary syndrome, surgical or subcutaneous intervention for any cardiovascular disease, or stroke within 30 days prior to randomization; and (v) planned surgical, subcutaneous, or device-based intervention for any cardiovascular disease during an observation period.

### Procedures

Participants were randomized within 7 days of an index hospitalization to receive either the switched Sac/Val or continued ACEI/ARB (control) therapy, and the protocol treatment was initiated within 48 h following of enrolment and allocation (see Supplementary data online, *Study design*). Randomization was performed via an interactive web-based program with dynamic allocation using a minimization method balanced by sites, age (<70, ≥70 years), sex (female, male), LVEF (<40, ≥40%), atrial fibrillation (yes, no), and estimated glomerular filtration rate (eGFR; <60, ≥60 mL/min/1.73 m^2^).

After randomization, ACEI/ARB-exposed patients were switched to Sac/Val (an initial dose of 24/26 mg twice daily) treatment or continued with ACEI/ARB treatment with dose adjustment in addition to other GDMTs for HF. The Sac/Val doses were adjusted and up-titrated to a maximum of 97/103 mg twice daily, in principle, according to the domestic drug package insert information and pre-specified up-titration criteria based on patient medical conditions, such as systolic BP, potassium level, and renal function (see Supplementary data online, *Dose adjustment protocol*).

After protocol treatment initiation, study participants were allowed to undergo outpatient care, depending on their condition, with planned follow-up visits at Weeks 4 and 8. Heart failure-related care other than the use of study drugs was performed in accordance with the Japanese guidelines for HF management.^12,13^ The local investigators were asked, in principle, to neither initiate mineralocorticoid receptor antagonists (MRAs) and sodium-glucose co-transporter 2 (SGLT2) inhibitors nor change the doses of non-study drugs during the follow-up period to avoid the influence of those HF medications on study endpoints. However, the above-mentioned changes were allowed at the discretion of the local investigators, depending on participant medical conditions.

### Study outcomes

The key efficacy endpoint of the study was the proportional change in the geometric means of NT-proBNP levels from baseline to Weeks 4 (a secondary endpoint) and 8 (primary endpoint). N-terminal pro-B-type natriuretic peptide level was measured centrally at a commercial laboratory (SRL, Inc., Tokyo, Japan) via electrochemiluminescence immunoassay (Roche, Basel, Switzerland) to ensure allocation concealment. Other secondary efficacy endpoints included (i) percentages of patients with at least a 50% and 30% NT-proBNP level reduction from baseline to Weeks 8 and 4, respectively, as well as a 40% time-averaged reduction through Weeks 4 and 8; (ii) change in the NYHA functional class at Weeks 4 and 8; and (iii) change in the Kansas City Cardiomyopathy Questionnaire (KCCQ)-12 scores and percentage of patients with at least a 5-point increase in the score over 8 weeks. In addition, physician-reported clinical outcome incidences, including pre-specified worsening HF-related events and death (see Supplementary data online, *Prespecified clinical outcomes*); adverse events (AEs) of special interest, including symptomatic hypotension; hyperkalaemia (serum potassium level ≥ 5.5 mEq/L); worsening renal function (at least a 50% increase in serum creatinine level or a 30% decline in eGFR); angioedema; and other serious AEs during the follow-up period were analysed.

### Statistical analysis

During sample size calculation, a 19% reduction in the ratio of the proportional changes in the geometric means of NT-proBNP level in the Sac/Val group relative to those in the control group from baseline to 8 weeks was hypothesized. Sacubitril/valsartan treatment effect was conservatively assumed to be smaller than that observed in the PIONEER-HF trial for inpatients with acute decompensated HF with reduced ejection fraction (HFrEF) (29% time-averaged reduction through 4 and 8 weeks relative to enalapril) and the PARAMOUNT trial for patients with chronic HF with preserved LVEF (23% reduction over 12 weeks relative to valsartan).^6,14^ However, in a Phase III PARALLEL-HF study in Japanese patients with chronic HFrEF,^15^ Sac/Val therapy reduced NT-proBNP level by 15% relative to enalapril treatment. In that study, the target dose of enalapril (10 mg twice daily) was higher than the standard dose (5–10 mg daily) for HF therapy approved in Japan, indicating a possibility of underestimating the Sac/Val treatment effect. These previous data led us to the aforementioned treatment effect on NT-proBNP level, resulting in a sample size of 200 participants per arm to detect a 19% (with a log-scaled SD of 0.70) difference between study arms at a two-sided significance level of 5% with 80% power and a possible drop-out or missing rate of 13%.

The statistical analysis plan developed before database lock is presented in Supplementary data online, *Statistical analysis plan*. All analyses were performed according to the intention-to-treat principle. The patient background and medication usage were described as median (interquartile range) or as *n* (%). Efficacy endpoints were analysed in the pre-specified full analysis set (FAS) of patients with available efficacy data, while clinical outcome and safety analyses were performed using data of all patients who initiated the protocol treatment. For the primary endpoint, a mixed-effects model for repeated measurements (MMRM) analysis was performed for the change in logarithmic NT-proBNP values at Weeks 4 and 8 with adjustment for the baseline value. The analysis was also performed in the pre-specified subgroups based on the background demographic and clinical features, including LVEF category. For the secondary endpoints, the achievements of the pre-specified criteria of NT-proBNP level reduction were analysed using logistic regression analyses adjusted for the baseline NT-proBNP levels. Changes in NYHA class from the baseline to Weeks 4 and 8 were analysed via ordinal logistic regression-based generalized estimating equations that account for correlations in the longitudinal measurement data. The KCCQ-12 score changes and score improvements (>5 points) were analysed with the baseline score adjustments using linear and logistic regression models. Vital signs and laboratory values were analysed using the MMRMs with adjustment for the baseline value. Clinical outcome incidence was described using the cumulative incidence or mean cumulative function estimated for each group and analysed using Cox proportional hazards or Fine–Gray models considering death as a competing risk. The incidence of AEs of special interest was analysed using Poisson regression models with robust variance. All statistical tests were examined at a two-sided significance level of 0.05. Analyses were performed using R software version 4.1.0 or higher (R Foundation for Statistical Computing, Vienna, Austria) and SAS software version 9.4 (SAS Institute, Cary, NC, USA).

## Results

### Study population, treatment, and follow-up

**Figure 1:**
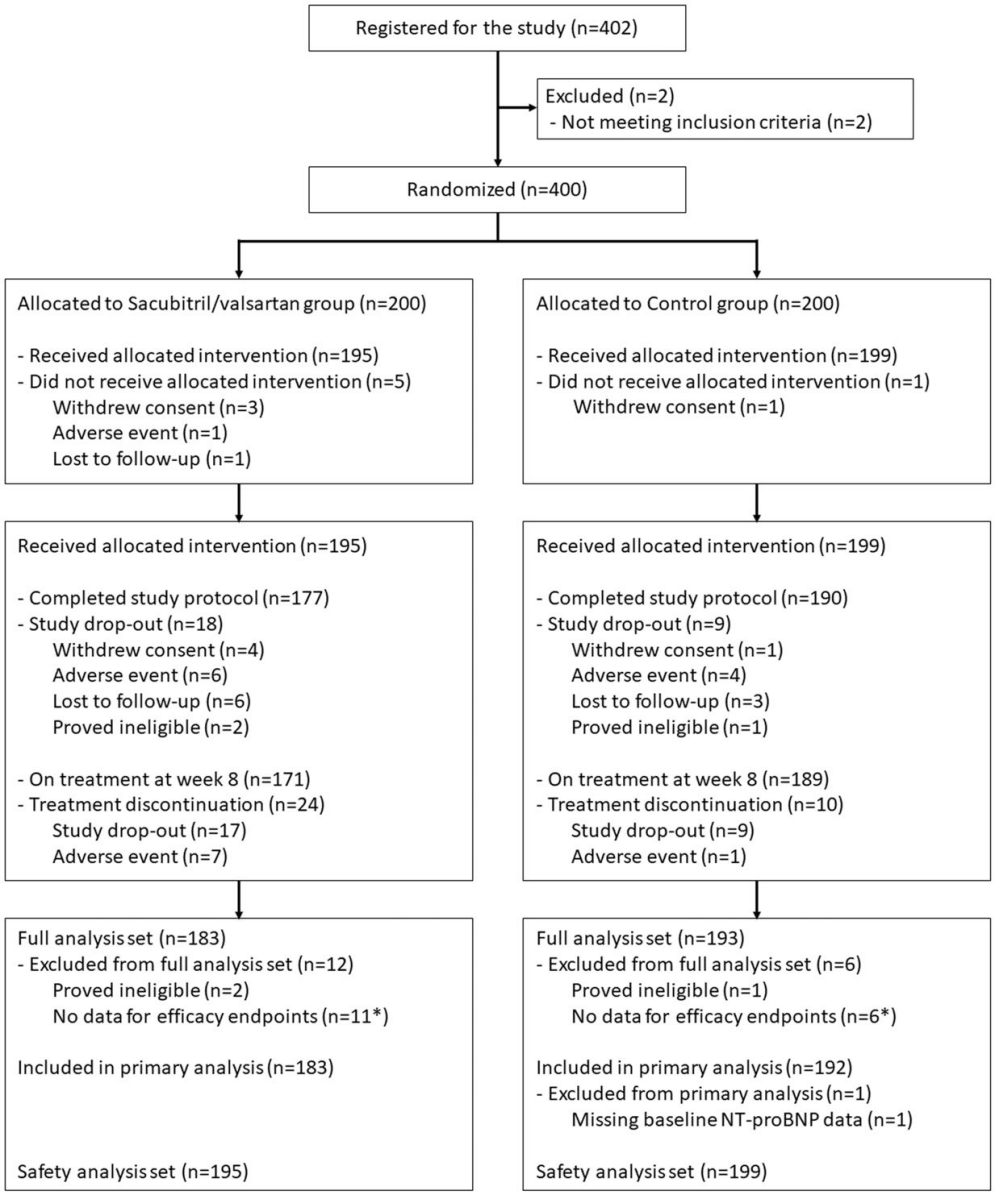
CONSORT diagram * One patient in each group was a duplicate of an ineligible case. NT-proBNP, N-terminal pro-B-type natriuretic peptide

A total of 400 patients were equally assigned to the Sac/Val or control group (*Figure 1*). End-of-study visits occurred on August 2023, and database lock for the primary analysis occurred on February 2024. Among randomized patients, 394 (98.5%) patients received at least one allocated intervention. A total of 367 [177 (88.5％) for the Sac/Val group and 190 (95.0％) for the control group] and 360 [171 (85.5％) for the Sac/Val group and 189 (94.5％) for the control group] patients completed the study protocol and were taking study drugs at Week 8, respectively. A total of 183 patients (91.5%) in the Sac/Val group and 193 patients (96.5%) in the control group were included in the FAS, and one patient in the control group was excluded from the primary efficacy analysis owing to missing baseline (at Week 0) NT-proBNP data.

Patient background characteristics are shown in *Table 1*. Overall, the mean (±SD) age was 73 ± 13 years, and 120 patients (31.9%) were women. Moreover, 167 (44.4%), 72 (19.1%), and 77 (20.5%) patients had a previous HF history, prior HF hospitalization history, and an ischaemic HF aetiology, respectively. The NYHA Classes II–IV were observed in 26.1%, 51.3%, and 22.6% of patients, respectively. The mean (±SD) LVEF was 39.7 ± 15.3%, and 55.1% of patients had LVEF < 40% at randomization. The ACEI/ARB use rate at the initial presentation to the hospital was 59.0%, and all patients were ACEI/ARB users at the time of randomization. Seventy-three per cent and 61% of patients were on MRA and SGLT2 inhibitor therapy, respectively. Randomizations were performed at a median of 4 days after index hospitalization, and the study intervention was initiated at a median of 6 days of hospitalization.

**Table 1:** Patient background characteristics

Variable	Sac/Val (*n* = 183)	Control (*n* = 193)
Age, year	75 (68, 82)	77 (66, 84)
Female sex	60 (32.8)	60 (31.1)
Clinical features of HF		
Previous HF	81 (44.3)	86 (44.6)
Prior hospitalization for HF	30 (16.4)	42 (21.8)
Ischaemic aetiology	39 (21.3)	38 (19.7)
NYHA class^a^		
II	57 (31.1)	41 (21.2)
III	77 (42.1)	116 (60.1)
IV	49 (26.8)	36 (18.7)
Medical history		
Hypertension	139 (76.0)	137 (71.0)
Diabetes	64 (35.0)	63 (32.6)
Dyslipidaemia	89 (48.6)	101 (52.3)
Atrial fibrillation	77 (42.1)	85 (44.0)
Ongoing atrial fibrillation	79 (43.2)	79 (40.9)
Chronic obstructive pulmonary disease	16 (8.7)	20 (10.4)
Ischaemic heart disease	39 (21.3)	44 (22.8)
Ischaemic stroke	13 (7.1)	13 (6.7)
Clinical and laboratory data^a^		
Body mass index, kg/m^2^	23.4 (21.1, 26.0)^b^	23.5 (21.3, 26.2)
Systolic blood pressure, mm Hg	129 (118, 141)	126 (114, 138)
Heart rate, b.p.m.	84 (71, 95)	77 (69, 95)
eGFR, mL/min/1.73 m^2^	51.5 (41.4, 63.4)	51.8 (42.5, 61.1)
LVEF, %	37 (28, 49)	37 (28, 51)
<40%	102 (55.7)	105 (54.4)
≥40%	81 (44.3)	88 (45.6)
NT-proBNP level at baseline, pg/mL	1760 (1030, 3195)	1850 (948, 3310)^b^
Prior medications^a^		
ACEI/ARB	183 (100.0)	193 (100.0)
ACEI/ARB at presentation to hospital	112 (61.2)	110 (57.2)
β-blocker	133 (72.7)	146 (75.6)
MRA	136 (74.3)	140 (72.5)
SGLT2 inhibitor	112 (61.2)	119 (61.7)
Diuretics	139 (76.0)	145 (75.1)
Ivabradine	5 (2.7)	0 (0.0)
Calcium channel blocker	50 (27.3)	40 (20.7)
Randomization after admission, days	4 (3, 5)	4 (3, 5)
Study intervention after admission, days	6 (4, 7)	6 (5, 7)

Values are expressed as median (interquartile range) or *n* (%).

ACEI/ARB, angiotensin-converting enzyme inhibitor or angiotensin receptor blocker; eGFR, estimated glomerular filtration rate; HF, heart failure; LVEF, left ventricular ejection fraction; MRA, mineralocorticoid receptor antagonist; NT-proBNP, N-terminal pro-B-type natriuretic peptide; NYHA, New York Heart Association; Sac/Val, sacubitril/valsartan; SGLT2, sodium-glucose co-transporter 2.

^a^At randomization.

^b^One missing.

Detailed information regarding the administration of study drugs during the follow-up period in the FAS are presented in Supplementary data online, *Tables S1* and *S2*. In the Sac/Val group (*n* = 183), Sac/Val was initiated after switching from ACEI [27 (14.8%) patients] and ARB [156 (85.2%) patients]; subsequently, the doses were adjusted over the 8-week period. Among patients who completed the study protocol (*n* = 176) at Week 8, the percentages of patients who received Sac/Val 49/51 and 97/103 mg twice daily were 35.8% and 13.6%, respectively. In the control group, 58 (30.1%) and 135 (69.9%) patients were initially on ACEI (mostly enalapril) and ARB (mostly candesartan), respectively. The median dose equivalent in those medications at Week 8 was 50% (interquartile range; 50%, 100%) of each maximum dose on their package insert, and the change in ACEI/ARB use over 8 weeks was relatively small. Among the FAS patients who completed the study protocol over 8 weeks, seven (4.0%) patients in the Sac/Val group and one (0.5%) patient in the control group prematurely discontinued the study drugs owing to AEs. The change in the use of other HF medications during the follow-up period was generally infrequent in both groups at Week 8 (see Supplementary data online, *Table S3*). The median furosemide-equivalent dose of loop diuretics was 20 mg daily in both groups at baseline and Week 8. There were no significant between-group differences in changes of body mass index and systolic and diastolic BP at Weeks 4 and 8, while eGFR decline and potassium level rise in Sac/Val-treated patients were significantly smaller than those in ACEI/ARB-treated patients at Week 8 (see Supplementary data online, *Table S4*).

### N-terminal pro-B-type natriuretic peptide levels

**Figure 2:**
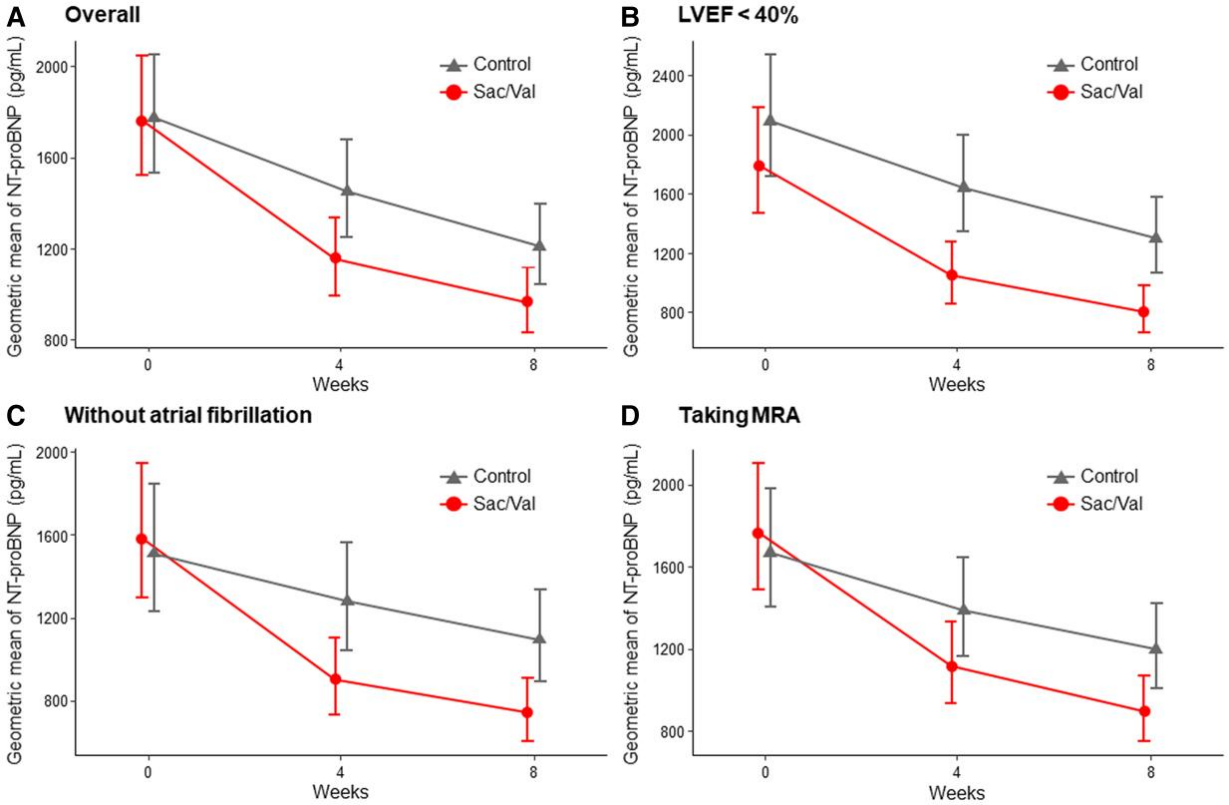
Change in N-terminal pro-B-type natriuretic peptide levels. Overall, the N-terminal pro-B-type natriuretic peptide level decreased in both treatment groups over 8 weeks (*A*). Reduction from baseline in the geometric means of N-terminal pro-B-type natriuretic peptide level at Weeks 4 and 8 was −35% (95% confidence interval −42% to −26%) vs. −45% (95% confidence interval −52% to −37%) for the sacubitril/valsartan group and −18% (95% confidence interval −28% to −8%) vs. −32% (95% confidence interval −40% to −23%) for the control group, and a group ratio (sacubitril/valsartan vs. control) of the proportional changes was 0.80 (95% confidence interval 0.68–0.94; *P* = .008) at Week 4 and 0.81 (95% confidence interval 0.68–0.95; *P* = .012) at Week 8, respectively. Group ratios of the proportional changes at Week 8 in subgroups with left ventricular ejection fraction < 40% (*B*), without atrial fibrillation (*C*), and taking mineralocorticoid receptor antagonist (*D*) were 0.69 (95% confidence interval 0.55–0.86), 0.66 (95% confidence interval 0.53–0.82), and 0.73 (95% confidence interval 0.60–0.88), respectively. Bars indicate 95% confidence interval. CI, confidence interval; LVEF, left ventricular ejection fraction; MRA, mineralocorticoid receptor antagonist; NT-proBNP, N-terminal pro-B-type natriuretic peptide; Sac/Val, sacubitril/valsartan

The changes and distributions of NT-proBNP levels in the overall cohort and subgroups divided by the history of atrial fibrillation are shown in *Table 2*. The NT-proBNP level decreased in both treatment groups over 8 weeks (*Figure 2A*). The per cent changes in the geometric means of NT-proBNP level at Weeks 4 and 8, compared with the baseline values, were −35% [95% confidence interval (CI) −42% to −26%] and −45% (95% CI −52% to −37%) for the Sac/Val group and −18% (95% CI −28% to −8%) and −32% (95% CI −40% to −23%) for the control group; furthermore, their group ratios (Sac/Val vs. control) were 0.80 (95% CI 0.68–0.94; *P* = .008) at Week 4 and 0.81 (95% CI 0.68–0.95; *P* = .012) at Week 8. The odds ratios for the achievement of 50%, 30%, and 40% NT-proBNP level reductions from baseline to Weeks 8 and 4, as well as 40% time-averaged reductions through Weeks 4 and 8 were 2.09 (95% CI 1.34–3.27; *P* = .001), 2.08 (95% CI 1.34–3.21; *P* = .001), and 1.79 (95% CI 1.15–2.79; *P* = .010), respectively (see Supplementary data online, *Table S5*).

**Table 2:** Change and distribution of N-terminal pro-B-type natriuretic peptide levels by visits and atrial fibrillation history

NT-proBNP (pg/mL)	Overall		With atrial fibrillation		Without atrial fibrillation	
	Sac/Val	Control	Sac/Val	Control	Sac/Val	Control
Baseline	(*n* = 183)	(*n* = 192)	(*n* = 77)	(*n* = 85)	(*n* = 106)	(*n* = 107)
	1760 (1030–3195)	1850 (948–3310)	1890 (1190–3240)	2270 (1320–3870)	1540 (914–3162)	1790 (756–2790)
<500	19 (10.4)	23 (12.0)	4 (5.2)	5 (5.9)	15 (14.2)	18 (16.8)
500 to <1000	24 (13.1)	27 (14.1)	9 (11.7)	10 (11.8)	15 (14.2)	17 (15.9)
1000 to <2000	59 (32.2)	50 (26.0)	28 (36.4)	26 (30.6)	31 (29.2)	24 (22.4)
≥2000	81 (44.3)	92 (47.9)	36 (46.8)	44 (51.8)	45 (42.5)	48 (44.9)
Week 4	(*n* = 181)	(*n* = 188)	(*n* = 75)	(*n* = 84)	(*n* = 106)	(*n* = 104)
	1260 (633–2110)	1540 (818–2650)	1520 (1090–2400)	1720 (1028–2672)	1017 (461–1758)	1335 (682–2552)
<500	33 (18.2)	21 (11.2)	5 (6.7)	5 (6.0)	28 (26.4)	16 (15.4)
500 to <1000	35 (19.3)	41 (21.8)	10 (13.3)	15 (17.9)	25 (23.6)	26 (25.0)
1000 to <2000	63 (34.8)	57 (30.3)	35 (46.7)	29 (34.5)	28 (26.4)	28 (26.9)
≥2000	50 (27.6)	69 (36.7)	25 (33.3)	35 (41.7)	25 (23.6)	34 (32.7)
Week 8	(*n* = 176)	(*n* = 190)	(*n* = 71)	(*n* = 82)	(*n* = 105)	(*n* = 108)
	1030 (432–1845)	1310 (635–2292)	1520 (886–2380)	1420 (851–2322)	866 (368–1570)	1095 (583–2152)
<500	48 (27.3)	34 (17.9)	8 (11.3)	10 (12.2)	40 (38.1)	24 (22.2)
500 to <1000	37 (21.0)	46 (24.2)	15 (21.1)	19 (23.2)	22 (21.0)	27 (25.0)
1000 to <2000	50 (28.4)	52 (27.4)	23 (32.4)	25 (30.5)	27 (25.7)	27 (25.0)
≥2000	41 (23.3)	58 (30.5)	25 (35.2)	28 (34.1)	16 (15.2)	30 (27.8)

Values are expressed as median (interquartile range) or *n* (%).

NT-proBNP, N-terminal pro-B-type natriuretic peptide; Sac/Val, sacubitril/valsartan.

**Figure 3:**
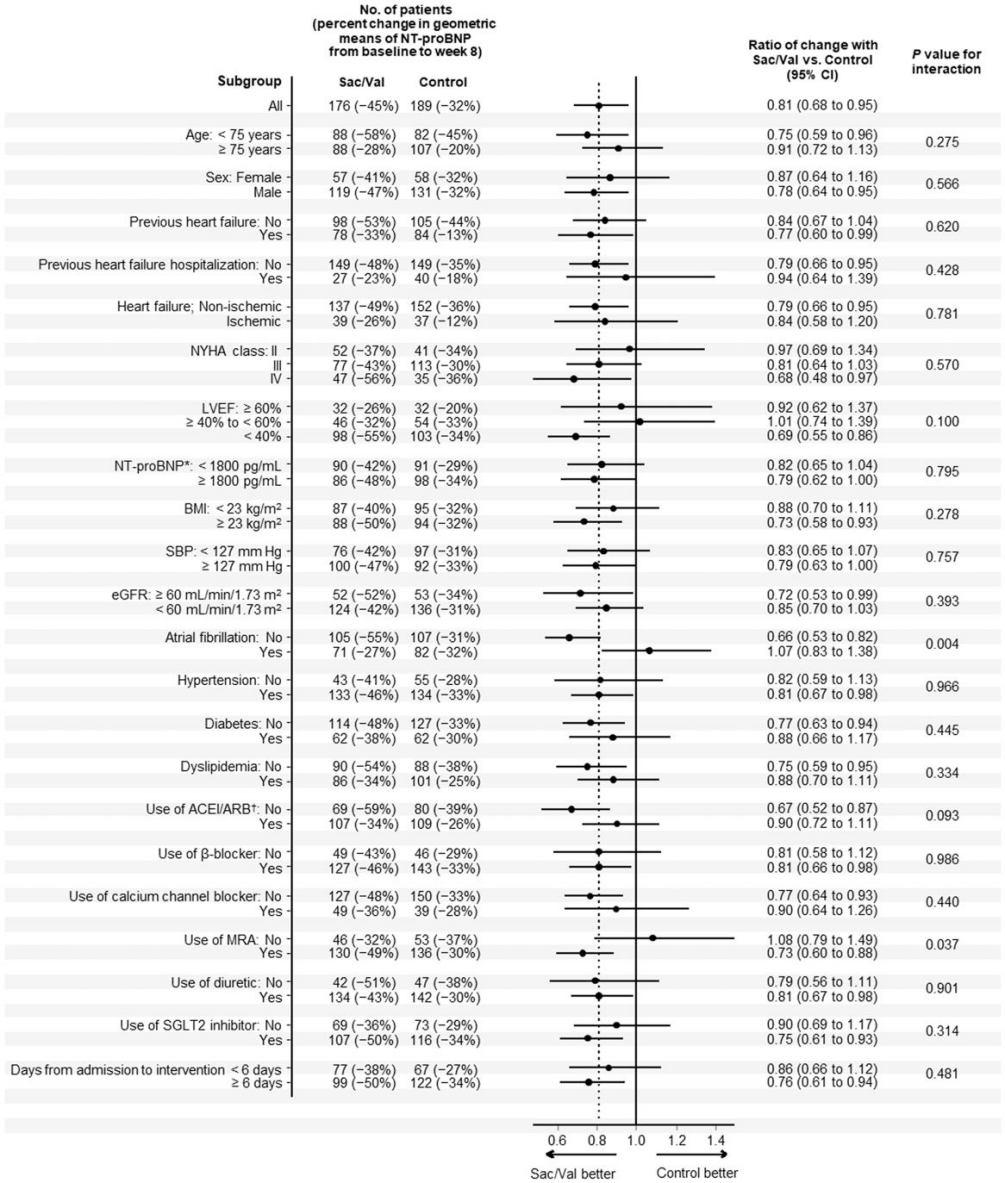
N-terminal pro-B-type natriuretic peptide response in pre-specified subgroups. Analysis of N-terminal pro-B-type natriuretic peptide level changes from baseline to Week 8 in pre-specified subgroups according to the background demographics and clinical characteristics. *At baseline. ^†^At presentation to hospital; others are based on data at randomization. ACEI, angiotensin-converting enzyme inhibitor; ARB, angiotensin receptor blocker; BMI, body mass index; CI, confidence interval; eGFR, estimated glomerular filtration rate; HF, heart failure; LVEF, left ventricular ejection fraction; MRA, mineralocorticoid receptor antagonist; NT-proBNP, N-terminal pro-B-type natriuretic peptide; NYHA, New York Heart Association; Sac/Val, sacubitril/valsartan; SBP, systolic blood pressure; SGLT2, sodium-glucose co-transporter 2

The Sac/Val treatment effect on NT-proBNP levels at Week 8 was generally consistent across pre-specified subgroups (*Figure 3*). Among the LVEF categories, the treatment effect was confined to the subgroup with LVEF < 40% (0.69: 95% CI 0.55–0.86), although there was no statistical difference (*P* for interaction = .100). The treatment effects were greater in the subgroups without atrial fibrillation (*P* for interaction = .004) and with MRA use at randomization (*P* for interaction = .037). The NT-proBNP responses in those subgroups are shown in *Figure 2B–D* and Supplementary data online, *Figure S1*.

### New York Heart Association class and Kansas City Cardiomyopathy Questionnaire-12 score

Responses of NYHA functional class over 8 weeks are shown in Supplementary data online, *Figure S2*. The common odds ratio, a measure of the likelihood that Sac/Val would lead to better shifts, was not statistically significant (see Supplementary data online, *Table S6*).

In the FAS, 286 (76.1%) patients, who completed the KCCQ-12 assessment at baseline and Week 8, were included in the KCCQ-12 analysis. At Week 8, there was no significant between-group difference in the mean change in the KCCQ-12 summary score and subdomains, and the percentage of patients who experienced ≥ 5-point score increase also did not differ between the treatment group (see Supplementary data online, *Tables S7* and *S8*).

### Clinical events and safety

In the intention-to-treat-based safety analysis set (*n* = 394; 195 in the Sac/Val group and 199 in the control group), the incidences of pre-specified clinical events and AEs of special interest were relatively low and did not differ between the treatment groups (*Table 3* and Supplementary data online, *Figure S3*). Of 59 serious AEs (29 in the Sac/Val group and 30 in the control group) developed after the initiation of study interventions, three (one case of dehydration and two cases of worsening renal function; all cases in the Sac/Val group) were considered by local investigators to be related to the study implementation (see Supplementary data online, *Table S9*). No angioedema was reported in either group.

**Table 3:** Clinical events and adverse events of special interest

Outcomes	Sac/Val (*n* = 195)	Control (*n* = 199)	HR (95% CI)	*P*-value
Clinical events				
Composite of first WHF^a^ or all-cause death	12 (6.2%)	17 (8.5%)	0.73 (0.35–1.52)	.400
Composite of first and recurrent WHF^a^ or all-cause death	14 events	18 events	0.82 (0.41–1.67)	.592
First WHF^a^	11 (5.6%)	15 (7.5%)	0.76 (0.35–1.65)	.485
First and recurrent WHF^a^	12 events	16 events	0.80 (0.38–1.69)	.551
All-cause death	2 (1.0%)	2 (1.0%)	1.06 (0.15–7.52)	.955
Cardiovascular death	1 (0.5%)	2 (1.0%)	0.52 (0.05–5.64)	.591
Adverse events of special interest				
Worsening renal function^b^	19 events	15 events	1.33 (0.70–2.55)^c^	.386
Hyperkalaemia^d^	12 events	14 events	0.90 (0.41–2.00)^c^	.799
Symptomatic hypotension	4 events	9 events	0.47 (0.15–1.49)^c^	.200
Hypotension	15 events	19 events	0.83 (0.43–1.62)^c^	.586

Values are expressed as *n* (%) or total number of events.

CI, confidence interval; eGFR, estimated glomerular filtration rate; HR, hazard ratio; Sac/Val, sacubitril/valsartan; WHF, worsening heart failure.

^a^Defined as (i) unplanned re-hospitalization; (ii) initiation of intravenous treatment (vasodilator or inotropic agent) for heart failure during index hospitalization, excluding at re-hospitalization; (iii) urgent visit due to heart failure requiring intravenous treatment (vasodilator, inotropic agent, or diuretic); or (iv) initiation of oral diuretic (loop diuretic, thiazide-type diuretic, or tolvaptan) or at least a 50% increase in its dose (outpatient).

^b^At least a 50% increase in serum creatinine levels or a 30% decline in eGFR.

^c^Incidence rate ratio.

^d^Serum potassium level of 5.5 mEq/L or more.

## Discussion

In the PREMIER study, there was a greater NT-proBNP level reduction following an early in-hospital Sac/Val therapy initiation, relative to the standard ACEI/ARB therapy, after haemodynamic stabilization during the initial acute care of Japanese patients admitted for AHF (*Structured Graphical Abstract*). Cognizant of similar results from the previous PIONERR-HF and PARAGLIDE-HF trials,^6–8^ this study may expand the emerging evidence on early Sac/Val therapy initiation in the above-mentioned clinical situation outside North America.

Prior studies of Sac/Val in non-AHF demonstrated its clinical value in Japanese patients.^15–17^ However, Japan and North America have generally substantial differences in the healthcare systems and practice patterns in terms of the clinical management of inpatients with AHF.^9,10^ For instance, diuretic use was less frequent and in lower doses in Japan than in the USA.^9^ Additionally, the length of hospital stay in Japan was longer than that in other countries, including the USA, which could allow for more clinical stabilization and the introduction of GDMT, cardiac rehabilitation, and supportive care prior to discharge.^10,18^ This might have influenced the decreasing trend of 30-day readmission rate in Japanese patients with AHF,^11^ with substantially lower incidence rates than those observed in the USA.^18,19^ Thus, the clinical management and outcomes of patients who experienced an AHF event potentially differ by healthcare systems and practice patterns, indicating the necessity to validate the available evidence based on individual medical situations.

An intensive and accelerated up-titration of GDMTs is currently recommended to improve post-AHF outcomes.^3–5^ However, up-titration to the max dose of Sac/Val was infrequent (13.6%) in the PREMIER study, compared with previous PIONEER-HF (55.2%) and PARAGLIDE-HF (39.1%) trials.^6,7^ Nevertheless, a favourable NT-proBNP response to Sac/Val therapy was observed in the present study. This suggests that further studies are needed to assess optimal doses of GDMTs, including Sac/Val, in Japanese post-AHF patients.

The treatment effect of Sac/Val therapy was evident across the below-normal LVEF spectrum in previous findings from the PIONEER-HF and PARAGLIDE-HF trials.^6–8^ Previous studies in patients with chronic HF also showed that the clinical benefits were prominent across the same LVEF spectrum.^20,21^ A favourable NT-proBNP response in a subgroup with LVEF < 40% observed in the present study, closely replicated that in the PIONEER-HF trial.^6^ In contrast, the NT-proBNP response in a subgroup with mildly reduced LVEF (≥40% to <60%) was neutral and seemingly differed from the PARAGLIDE-HF trial.^7^ These findings may enhance the Sac/Val therapy in Japanese patients with AHF and reduced LVEF.

The use of SGLT2 inhibitors in patients with recent AHF events proved to be effective in improving outcomes and enhancing clinical benefits.^22–24^ While the PIONEER-HF and PARAGLIDE-HF trials included relatively few patients undergoing SGLT2 inhibitor treatment,^8^ in the PREMIER study, ∼60% of patients were on SGLT2 inhibitor therapy at randomization; Sac/Val therapy reduced NT-proBNP levels irrespective of SGLT2 inhibitor use. In contrast, the proportion of background MRA users in the PREMIER study was much higher than that in previous studies,^8^ and the PREMIER study revealed that Sac/Val therapy provided a greater NT-proBNP level reduction in MRA users than in non-users. Moreover, the study demonstrated a favourable NT-proBNP response in a subgroup of patients without atrial fibrillation, which was comparable with that in the previous studies in the acute and chronic settings of HF.^25,26^ These findings may affect future guidelines for the use of Sac/Val and its combination with other GDMTs in the clinical setting of AHF.

The overall incidence of pre-specified clinical events in this study was relatively low, which was similar to the previously reported incidence in Japanese patients.^11,18^ This might have potentially obscured the Sac/Val therapy effect on clinical events, unlike previous studies showing its clinical benefits.^6–8^ Nevertheless, an acute favourable NT-proBNP response to Sac/Val therapy in inpatients with AHF predicted a reduced risk of post-discharge clinical events.^26^ An early Sac/Val therapy initiation and the consequent favourable NT-proBNP response may be important determinants of post-AHF outcomes.

This study has some limitations. First, compared with previous studies with LVEF-based inclusion,^6,7^ the sample size was small. Furthermore, the inclusion of patients across the LVEF spectrum complicates the interpretation of the study findings according to the LVEF categories. Second, an open-label pragmatic design in line with clinical practice in Japan might have affected the local investigators’ motivation for implementing dose adjustments of study drugs. This is partly associated with the known under-dose prescription patterns of recommended HF medications among Asian countries, including Japan.^27,28^ Additionally, the study drugs in both groups were available through standard care prescriptions, but the affordability and accessibility of these medications to the study participants were not investigated.^29^ Those factors might have affected the continuation and up-titration of study drugs. Third, the achieved NT-proBNP levels may be more prognostic than their changes,^30–32^ although previous studies also assessed the change in NT-proBNP levels following Sac/Val therapy as a study endpoint.^6,7,14^ Fourth, the study was not primarily designed to detect a reduction in clinical event occurrence, and the events were not adjudicated by a third party. Finally, this study was performed within Japan; hence, the applicability of the present findings to other ethnicities, regions, and countries remains uncertain.

In conclusion, early in-hospital Sac/Val therapy initiation, in addition to contemporary recommended HF therapy, triggered a greater NT-proBNP level reduction, without increasing AE risk, in stabilized Japanese patients after an AHF event. These findings may expand the evidence on Sac/Val therapy in this clinical situation outside North America.

## Supplementary Material

ehae561_Supplementary_Data

## Declarations

### Data Availability

The data underlying this work are available upon reasonable request from researchers who submit a detailed proposal outlining their intended use of the data and after approval by the principal investigator of the PREMIER study. Inquiries are to be addressed to the corresponding authors.

### Pre-registered Clinical Trial Number

The pre-registered clinical trial numbers are ClinicalTrial.gov (NCT05164653) and the Japan Registry of Clinical Trials (jRCTs021210046).
