# Supplementary material for: In-hospital initiation of angiotensin receptor–neprilysin inhibition in acute heart failure: the PREMIER trial
Source: Eur Heart J. 2024 Aug 31;45(42):4482–93. doi: 10.1093/eurheartj/ehae561 (PMC11544311; doi:10.1093/eurheartj/ehae561)
Supplement: ehae561_Supplementary_Data [file ehae561_supplementary_data.docx]

**Supplementary data online**

**In-hospital initiation of angiotensin receptor-neprilysin inhibition in acute heart failure: the PREMIER trial**

Atsushi Tanaka, MD, PhD,^a^ Keisuke Kida, MD, PhD,^b^ Yuya Matsue, MD, PhD,^c^ Takumi Imai, PhD,^d,e^ Satoru Suwa, MD,^f^ Isao Taguchi, MD, PhD,^g^ Itaru Hisauchi, MD, PhD,^g^ Hiroki Teragawa, MD, PhD,^h^ Yoshiyuki Yazaki, MD,^i^ Masao Moroi, MD, PhD,^i^ Koichi Ohashi, MD, PhD,^j^ Daisuke Nagatomo, MD,^k^ Toru Kubota, MD, PhD,^k^ Takeshi Ijichi, MD, PhD,^l^ Yuji Ikari, MD, PhD,^l^ Keisuke Yonezu, MD, PhD,^m^ Naohiko Takahashi, MD, PhD,^m^ Shigeru Toyoda, MD, PhD,^n^ Tsutomu Toshida, MD, PhD,^o^ Hiroshi Suzuki, MD, PhD,^o^ Tohru Minamino, MD, PhD,^c^ Kazutaka Nogi, MD, PhD,^p^ Kazuki Shiina, MD, PhD,^q^ Yu Horiuchi, MD,^r^ Kengo Tanabe, MD, PhD,^r^ Daisuke Hachinohe, MD,^s^ Shunsuke Kiuchi, MD, PhD,^t^ Kenya Kusunose, MD, PhD,^u^ Michio Shimabukuro, MD, PhD,^v^ Koichi Node, MD, PhD,^a^ on behalf of the PREMIER Study Investigators.

^a^ Department of Cardiovascular Medicine, Saga University, Saga, Japan.

^b^Department of Pharmacology, St. Marianna University School of Medicine, Kawasaki, Japan

^c^Department of Cardiovascular Biology and Medicine, Juntendo University Graduate School of Medicine, Tokyo, Japan

^d^Clinical Research Division, Organization for Clinical Medicine Promotion, Tokyo, Japan

^e^ Clinical and Translational Research Center, Kobe University Hospital, Kobe, Japan

^f^Department of Cardiology, Juntendo University Shizuoka Hospital, Shizuoka, Japan

^g^Department of Cardiology, Dokkyo Medical University Saitama Medical Center, Koshigaya, Japan

^h^Department of Cardiovascular Medicine, JR Hiroshima Hospital, Hiroshima, Japan

^i^Division of Cardiovascular Medicine, Toho University Ohashi Medical Center, Tokyo, Japan

^j^Department of Cardiology, Tokyo Metropolitan Bokutoh Hospital, Tokyo, Japan

^k^Division of Cardiology, Cardiovascular and Aortic Center, Saiseikai Fukuoka General Hospital, Fukuoka, Japan

^l^Department of Cardiology, Tokai University, Isehara, Japan

^m^Department of Cardiology and Clinical Examination, Faculty of Medicine, Oita University, Yufu, Japan

^n^Department of Cardiovascular Medicine, Dokkyo Medical University, Mibu, Japan

^o^Division of Cardiology, Department of Internal Medicine, Showa University Fujigaoka Hospital, Yokohama, Japan

^p^Department of Cardiovascular Medicine, Nara Medical University, Kashihara, Japan

^q^Department of Cardiology, Tokyo Medical University, Tokyo, Japan

^r^Division of Cardiology, Mitsui Memorial Hospital, Tokyo, Japan

^s^Department of Cardiology, Sapporo Heart Center, Sapporo Cardio Vascular Clinic, Sapporo, Japan

^t^Department of Cardiovascular Medicine, Toho University Faculty of Medicine, Tokyo, Japan

^u^Department of Cardiovascular Medicine, Nephrology, and Neurology, Graduate School of Medicine, University of the Ryukyus, Okinawa, Japan

^v^Department of Diabetes, Endocrinology, and Metabolism Fukushima Medical University School of Medicine, Fukushima, Japan

**Address for correspondence:** Dr. Atsushi Tanaka and Dr. Koichi Node, Department of Cardiovascular Medicine, Saga University, 5-1-1 Nabeshima, Saga 849-8501, Japan.

TEL: +81-952-34-2364

FAX: +81-952-34-2089

Email: tanakaa2@cc.saga-u.ac.jp (Dr. Tanaka) and node@cc.saga-u.ac.jp (Dr. Node)

**Table of contents**

P3. Table of contents

P4-5. Study sites list

P6-77. Study protocol

P78-79. Inclusion and exclusion criteria

P80. Study design

P81. Dose adjustment protocol

P82. Prespecified clinical outcomes

P83-103 . Statistical analysis plan

P104. Administration of Sac/Val (Table S1)

P105. Administration of study drug in the control group (Table S2)

P106. Use of other heart failure medications at baseline and week 8

(Table S3)

P107-108. Changes in clinical and laboratory data (Table S4)

P109. Achievement of prespecified NT-proBNP level reduction (Table S5)

P110. Category change from baseline in NYHA functional class (Table S6)

P111. Changes in KCCQ-12 scores (Table S7)

P112. Percentage of patients who experienced improvement of KCCQ-12 scores (≥ 5 points) at week 8 (Table S8)

P113. Serious adverse events developed and reported (Table S9)

P114-115. Individual plotting of changes in NT-proBNP level (Figure S1)

P116. Change in NYHA functional class over 8 weeks (Figure S2)

P117-118. Effect of Sac/Val therapy on prespecified clinical events (Figure S3)

**Study sites list**

Site, City (representative investigator)

1. Saga University Hospital, Saga (Dr. Koichi Node)
2. Imari-Arita Kyoritsu Hospital, Matsuura-gun (Dr. Kazuo Matsunaga)
3. Iwate Medical University Hospital, Shiwa-gun (Dr. Yoshihiro Morino)
4. Urasoe General Hospital, Urasoe (Dr. Hiroki Uehara)
5. National Hospital Organization Ureshino Medical Center, Ureshino (Dr. Mitsuhiro Shimomura)
6. Osaka Medical and Pharmaceutical University Hospital, Takatsuki (Dr. Yumiko Kanzaki)
7. Gifu Heart Center, Gifu (Dr. Masayasu Nakagawa)
8. Gunma University Hospital, Maebashi (Dr. Masaru Obokata)
9. Kobe City Medical Center General Hospital, Kobe (Dr. Yutaka Furukawa)
10. Tosei General Hospital, Seto (Dr. Masayoshi Ajioka)
11. National Cerebral and Cardiovascular Center, Suita (Dr. Chisato Izumi)
12. Saga-Ken Medical Centre Koseikan, Saga (Dr. Yutaka Hikichi)
13. Sasebo Chuo Hospital, Sasebo (Dr. Yoshihisa Kizaki)
14. Sapporo Cardio Vascular Clinic, Sapporo (Dr. Daisuke Hachinohe)
15. Juntendo University School of Medicine Juntendo Clinic, Tokyo (Dr. Tohru Minamino)
16. JR Hiroshima Hospital, Hiroshima (Dr. Hiroki Teragawa)
17. Showa University Fujigaoka Hospital, Yokohama (Dr. Hiroshi Suzuki)
18. St. Marianna University Hospital, Kawasaki (Dr. Keisuke Kida)
19. St. Marianna University Yokohama Seibu Hospital, Yokahama (Dr. Hisao Matsuda)
20. Teine Keijinkai Hospital, Sapporo (Dr. Satoshi Yuda)
21. Tokai University Hospital, Isehara (Dr. Yuji Ikari)
22. Tokyo Medical University Hospital, Tokyo (Dr. Kazuki Shiina)
23. Tokyo Medical University Hachioji Medical Center, Hachioji (Dr. Nobuhiro Tanaka)
24. Tokyo Metropolitan Bokutoh Hospital, Tokyo (Dr. Koichi Ohashi)
25. Toho University Ohashi Medical Center, Tokyo (Dr. Masao Moroi)
26. Toho University Omori Medical Center, Tokyo (Dr. Shunsuke Kiuchi)
27. Tokushima University Hospital, Tokushima (Dr. Masataka Sata)
28. Dokkyo Medical University Saitama Medical Center, Koshigaya (Dr. Isao Taguchi)
29. Dokkyo Medical University Hospital, Shimotsuga-gun (Dr. Shigeru Toyoda)
30. Nagoya Memorial Hospital, Nagoya (Dr. Kenji Shiino)
31. Nagoya University Hospital, Nagoya (Dr. Toyoaki Murohara)
32. Nara Medical University Hospital, Kashihara (Dr. Kazutaka Nogi)
33. Hamamatsu University Hospital, Hamamatsu (Dr. Yuichiro Maekawa)
34. Hirosaki University Hospital, Hirosaki (Dr. Hirofumi Tomita)
35. Hiroshima University Hospital, Hiroshima (Dr. Yukiko Nakano)
36. Saiseikai Fukuoka General Hospital, Fukuoka (Dr. Toru Kubota)
37. Saiseikai Futsukaichi Hospital, Chikushino (Dr. Toshiaki Kadokami)
38. Fujita Health University Hospital, Toyoake (Dr. Hideo Izawa)
39. Hokkaido University Hospital, Sapporo (Dr. Toshihisa Anzai)
40. Mitsui Memorial Hospital, Tokyo (Dr. Kengo Tanabe)
41. Oita University Hospital, Yufu (Dr. Naohiko Takahashi)
42. Juntendo University Shizuoka Hospital, Izunokuni (Dr. Satoru Suwa)
43. Nagoya City University Hospital, Nagoya (Dr. Shuichi Kitada)
44. Osaka Metropolitan University Hospital, Osaka (Dr. Daiju Fukuda)

|  |
| --- |
| **Pr**ogram of Angiot**e**nsin-Neprilysin Inhibition in Ad**m**itted Pat**ie**nts  with Wo**r**sening Heart Failure  PREMIER study |
| Study protocol |
|  |
| Principal research physician: Koichi Node  Affiliation: Department of Cardiovascular Medicine, Saga University Hospital  Address: 5-1-1 Nabeshima, Saga-city, Saga, 849-8501  Contact: TEL +81-952-34-2364 |
| Creation Date: Sep. 9, 2021 |
| Version number: Version 1.1 |

Re: Confidentiality

The information contained in this study plan shall not be disclosed to any third parties other than those directly involved in this research, members of the Certified Review Board and review organizations of medical institutions implementing this research, and government officials including the Minister of Health, Labor and Welfare. Furthermore, the information contained in this study plan may not be used for any purpose other than conducting and evaluating this study without the prior written consent of the principal investigator.

Revision History

| Version number | Creation/Revision date | Reason for revision |
| --- | --- | --- |
| Version 1.0 (first version) | 2021/6/30 | Newly created |
| Ver 1.1 | Sept 9, 2021 | For corrections pertaining to CRB deliberation |

Contents

[**Study overview** 15](#_Toc151130312)

[**1.** **Background and purpose of the study** 19](#_Toc151130313)

[1.1. Study background 19](#_Toc151130314)

[1.2. Study purpose 20](#_Toc151130315)

[**2.** **Study contents** 20](#_Toc151130316)

[2.1. Study endpoints 20](#_Toc151130317)

[2.2. Study method (type/design of clinical research) 22](#_Toc151130318)

[2.3. Study method (clinical research procedures) 22](#_Toc151130319)

[2.4. Administration method 23](#_Toc151130320)

[2.4.1. Administration method for the sacubitril/valsartan group 23](#_Toc151130321)

[2.4.2. Criteria for discontinuation of sacubitril/valsartan 24](#_Toc151130322)

[2.4.3. Administration method for the control group (sacubitril/valsartan non-administration group) 24](#_Toc151130323)

[2.5. Discontinuation criteria for the entire study 25](#_Toc151130324)

[2.5.1 Cancellation/termination procedures 25](#_Toc151130325)

[2.6. Randomization procedure 26](#_Toc151130326)

[2.7. Original source materials 26](#_Toc151130327)

[2.8. Handling of case report forms 26](#_Toc151130328)

[2.9. CRF content confirmation and inquiries 27](#_Toc151130329)

[**3.** **Inclusion criteria** 27](#_Toc151130330)

[3.1. Subjects 27](#_Toc151130331)

[3.2. Selection criteria 27](#_Toc151130332)

[3.3. Exclusion criteria 28](#_Toc151130333)

[3.4. Discontinuation criteria for each study participant 29](#_Toc151130334)

[3.5. Handling of study participants who discontinue protocol treatment 30](#_Toc151130335)

[**4.** **Treatment for study subjects** 30](#_Toc151130336)

[4.1. Observation/examination schedule 30](#_Toc151130337)

[4.2. Patient registration 32](#_Toc151130338)

[4.2.1. Investigation items at the time of registration/allocation 32](#_Toc151130339)

[4.2.2. Patient background information 33](#_Toc151130340)

[4.3. Observation/examination items for prescribed visits 33](#_Toc151130341)

[4.4. Occasional observation/examination items 35](#_Toc151130342)

[4.5. Central measurement examination 35](#_Toc151130343)

[4.6. Study drug overview 36](#_Toc151130344)

[4.7. Regulation of concomitant drugs and concomitant therapies 45](#_Toc151130345)

[4.7.1. Sacubitril/valsartan group 45](#_Toc151130346)

[4.7.2. Control group 45](#_Toc151130347)

[**5.** **Efficacy and safety endpoint** 45](#_Toc151130348)

[5.1 Efficacy endpoints 45](#_Toc151130349)

[5.2. Safety endpoints 47](#_Toc151130350)

[5.2.1. Evaluation of adverse events 47](#_Toc151130351)

[5.2.2. Severity 47](#_Toc151130352)

[5.2.3. Seriousness 47](#_Toc151130353)

[5.2.4. Causal relationship with implementation of the study 47](#_Toc151130354)

[5.2.5. Causal relationship with the study drug (sacubitril/valsartan) 47](#_Toc151130355)

[5.2.6. Predictability 48](#_Toc151130356)

[5.2.7. Outcome and outcome date 48](#_Toc151130357)

[5.3. Handling when an adverse event occurs 48](#_Toc151130358)

[5.3.1. Definition of adverse events, diseases, etc. and side effects 48](#_Toc151130359)

[5.4. Measures to be taken if an adverse event occurs 49](#_Toc151130360)

[5.4.1. Measures taken for the study participants 49](#_Toc151130361)

[5.4.2. Reporting of adverse events 49](#_Toc151130366)

[5.4.3. Reporting of illness etc. 49](#_Toc151130367)

[**6.** **Compliance with the study plan and management of nonconformities** 50](#_Toc151130368)

[6.1. Compliance with the study plan 50](#_Toc151130371)

[6.2. Reporting and recording of nonconformities 50](#_Toc151130372)

[6.3. Obtaining permission for implementation at the medical institution 51](#_Toc151130373)

[**7.** **Statistical analysis** 51](#_Toc151130374)

[7.1. Target number of cases 51](#_Toc151130375)

[7.2. Analysis set 52](#_Toc151130376)

[7.2.1. Efficacy analysis set 52](#_Toc151130380)

[7.2.1.1. Full analysis set (FAS) 52](#_Toc151130381)

[7.2.1.2. Per-protocol set (PPS) 53](#_Toc151130382)

[7.2.2. Safety set (SS) 53](#_Toc151130383)

[7.3. Analysis method 53](#_Toc151130384)

[7.3.1. Case composition 53](#_Toc151130386)

[7.3.2. Analysis target description 53](#_Toc151130387)

[7.3.3. Patient characteristics and baseline values 54](#_Toc151130388)

[7.4. Analysis of efficacy 54](#_Toc151130389)

[7.4.1. Analysis of efficacy 54](#_Toc151130391)

[7.4.2. Safety analysis 56](#_Toc151130392)

[**8.** **Interim analysis** 57](#_Toc151130393)

[**9.** **Viewing of original source materials, etc.** 57](#_Toc151130394)

[**10.** **Quality control and quality assurance** 57](#_Toc151130395)

[10.1. Monitoring methods 57](#_Toc151130396)

[**11.** **Matters related to ethical considerations** 57](#_Toc151130397)

[11.1. Benefits, burdens, and anticipated disadvantages to study participants 58](#_Toc151130398)

[11.2. Handling of personal information 58](#_Toc151130399)

[**12.** **Matters regarding handling and storage of records (including data)** 59](#_Toc151130400)

[**13.** **Matters regarding the payment of money and compensation** 59](#_Toc151130401)

[13.1. Regarding financial burden associated with the study 59](#_Toc151130402)

[13.2. Compensation and indemnity for health damage caused by the study 60](#_Toc151130403)

[13.2.1.Clinical research insurance coverage 60](#_Toc151130404)

[**14.** **Matters regarding publication of information concerning the study** 61](#_Toc151130412)

[14.1. Registration of study plan 61](#_Toc151130413)

[14.2. Publication of results 61](#_Toc151130414)

[**15.** **Study implementation period** 62](#_Toc151130415)

[15.1. Study implementation period 62](#_Toc151130416)

[15.2. Completion of the study 62](#_Toc151130417)

[**16.** **Explanation and consent for study participants** 62](#_Toc151130418)

[16.1. Procedures for obtaining informed consent 62](#_Toc151130419)

[16.2. Instructions for study participants 63](#_Toc151130420)

[16.3. Revision of explanatory and consent documents 64](#_Toc151130421)

[16.4. Withdrawal of consent 64](#_Toc151130422)

[16.5. Possibility of samples and information obtained from study participants being used for future studies 64](#_Toc151130423)

[**17.** **Research funding and conflicts of interest** 65](#_Toc151130424)

[17.1. Sources of funding and financial relationships 65](#_Toc151130425)

[17.2. Attribution of results 66](#_Toc151130426)

[17.3. Managing conflicts of interest 66](#_Toc151130427)

[17.4. Conflict of interest status 66](#_Toc151130428)

[**18.** **Certified Review Board** 66](#_Toc151130429)

[18.1. Certified Review Board 66](#_Toc151130430)

[18.2. Report to the CRB 67](#_Toc151130431)

[18.2.1.Regular report 67](#_Toc151130432)

[18.2.2.Other reports 68](#_Toc151130439)

[**19.** **Changes to study plans, etc.** 68](#_Toc151130440)

[**20.** **Contents and methods of reporting to the administrator of the medical institution** 69](#_Toc151130441)

[**21.** **Responses regarding the provision of medical care to study participants after study implementation** 70](#_Toc151130442)

[**22.** **References and literature list** 70](#_Toc151130443)

[**23.** **Amendment** 73](#_Toc151130445)

Abbreviation definitions

| Abbreviation | Full expression (English) | Full expression (Japanese) |
| --- | --- | --- |
| ACE | angiotensin converting enzyme | Angiotensin converting enzyme |
| ALP | alkaline phosphatase | alkaline phosphatase |
| ALT | alanine aminotransferase | alanine aminotransferase |
| ARB | angiotensin II receptor blocker | Angiotensin receptor blocker |
| AST | aspartate aminotransferase | aspartate aminotransferase |
| BMI | body mass index | Body mass index |
| BUN | blood urea nitrogen | blood urea nitrogen |
| CABG | coronary artery bypass grafting | Coronary artery bypass grafting |
| cGMP | cyclic guanosine monophosphate | cyclic guanosine monophosphate |
| CK | creatine kinase | creatine kinase |
| ClinicalTrials.gov | ― | US clinical trial registry database |
| COPD | chronic obstructive pulmonary disease | Chronic obstructive pulmonary disease |
| Cr | creatinine | Creatinine |
| CRB | certified review board | certified Review Board |
| CRF | case report form | case report form |
| CRP | C-reactive protein | C-reactive protein |
| CRT | cardiac resynchronization therapy | Cardiac resynchronization therapy |
| DICOM | Digital Imaging and Communications in Medicine | DICOM (the international standard for medical images and examination information data including videos, and for communicating, printing, storing, and searching related data) |
| E/e’ | ― | Ratio of early diastolic mitral inflow velocity to early diastolic mitral annulus velocity |
| EDC | electric data capture | electric data capture |
| eGFR | estimated glomerular filtration rate | Estimated glomerular filtration rate |
| ePV | estimated plasma volume | estimated plasma volume |
| FAS | full analysis set | Full analysis set |
| FIB-4 index | Fibrosis-4 | liver fibrosis prediction score |
| GDF-15 | growth differentiation factor 15 | Growth differentiation factor 15 |
| GLS | global longitudinal strain | Index of myocardial longitudinal contractile function |
| Hb | hemoglobin | hemoglobin |
| HbA1c | hemoglobin A1c | hemoglobin A1c |
| HDL-C | high-density lipoprotein cholesterol | high-density lipoprotein cholesterol |
| HFpEF | heart failure with preserved ejection fraction | Diastolic heart failure |
| HFrEF | heart failure with reduced ejection fraction | Heart failure with reduced ejection fraction |
| Ht | hematocrit | Hematocrit |
| ICD | implantable cardiac defibrillator | Implantable cardiac defibrillator |
| IVC | inferior vena cava | Inferior vena cava diameter |
| jRCT | Japan Registry of Clinical Trials | Clinical study implementation plan/study summary publication system |
| KCCQ-12 | kansas city cardiomyopathy questionnaire-12 | Kansas City cardiomyopathy questionnaire-12 |
| LAVI | left atrial volume index | Left atrial volume index |
| LDH | lactate dehydrogenase | serum lactate dehydrogenase |
| LLT | lowest level term | Lowest level term |
| LVEDV | left ventricular end-diastolic volume | Left ventricular end-diastolic volume |
| LVEF | left ventricular ejection fraction | Left ventricular ejection fraction |
| LVESV | left ventricular end-systolic volume | Left ventricular end-systolic volume |
| LVMI | left ventricular mass index | Left ventricular mass index |
| LVOT | left ventricular outflow tract | Left ventricular outflow tract diameter |
| LVOT-VTI | left ventricular outflow tract velocity time integral | Left ventricular outflow tract velocity time integral |
| MedDRA/J | Medical Dictionary for Regulatory Activities/Japanese version | Medical Dictionary for Regulatory Activities/Japanese version |
| MMRM | mixed-effects models for repeated measures | Mixed model for repeated measures |
| MRA | mineralocorticoid receptor antagonist | Mineralocorticoid receptor antagonist |
| non-HDL-C | non-high-density lipoprotein cholesterol | values: TC minus HDL-C |
| NT-proBNP | N-terminal fragment of pro-B-type natriuretic peptide | N-terminal fragment of pro-B-type natriuretic peptide |
| NSAIDs | non-steroidal anti-inflammatory drugs | non-steroidal anti-inflammatory drugs |
| NYHA | New York Heart Association | New York Heart Association |
| OATP1B1 | organic anion transporting polypeptides 1B1 | drug transporter 1B1 |
| OATP1B3 | organic anion transporting polypeptides 1B3 | drug transporter 1B3 |
| PCI | percutaneous coronary intervention | Percutaneous coronary intervention |
| PDE5 | phosphodiesterase type 5 | phosphodiesterase 5 |
| PLT | platelet | platelets |
| PPS | per protocol set | Per protocol set |
| PT | preferred term | Basic terminology |
| RBC | red blood cell | red blood cells |
| septal e’、lateral e’ | ― | Septal and lateral mitral annulus movement speed |
| SGLT2 | sodium glucose cotransporter 2 | Sodium glucose cotransporter 2 |
| SOC | system organ class | System organ class |
| SS | safety set | Safety set |
| Soluble ST2 | soluble suppression of tumorigenesis-2 | Soluble interleukin 1 receptor family |
| TC | total cholesterol | total cholesterol |
| TEAE | treatment-emergent adverse event | Adverse events that occurred following protocol treatment initiation |
| TG | triglyceride | triglyceride |
| TR velocity | tricuspid regurgitation velocity | maximum blood flow velocity for tricuspid regurgitation velocity |
| γ-GTP | γ-glutamyl transpeptidase | γ-glutamyl transpeptidase |
| 1.5AG | 1.5-anhydro-D-glucitol | 1.5-anhydro-D-glucitol |

--: Indicates that the appropriate term does not exist.

# **Study overview**

| Study overview | |
| --- | --- |
| Study name | Study on Angiotensin-Neprilysin Inhibitors in Hospitalized Patients with Worsening Heart Failure |
| Abbreviation | PREMIER study |
| Study purpose | To examine the effects of sacubitril/valsartan compared with the standard treatment for hospitalized patients with worsening heart failure (HF), using changes in NT-proBNP as an indicator. |
| Study drug | Sacubitril/valsartan sodium hydrate (brand name: Entresto® Tablets) |
| Study design | Investigator-initiated, multicenter, prospective, parallel-group, randomized controlled trial |
| Study endpoints | Primary endpoint:  Between-group ratio of the proportional change in the geometric mean NT-proBNP at 8 weeks following protocol treatment initiation, compared with baseline  Secondary endpoint:   1. Between-group ratio of the proportional change in geometric mean NT-proBNP at 4 weeks following protocol treatment initiation, compared with baseline. 2. Percentage of cases in which the NT-proBNP value decreased by 50% or more from the baseline, at 8 weeks following protocol treatment initiation. 3. Percentage of cases in which the NT-proBNP value decreased by 30% or more from the baseline, at 4 weeks following protocol treatment initiation. 4. Percentage of cases which mean NT-proBNP value decreased by 40% or more from the baseline at 4 and 8 weeks following protocol treatment initiation. 5. Change from the baseline of cardiac troponin T, CRP, GDF-15, soluble ST2, glycoalbumin, and 1.5AG at 8 weeks after following protocol treatment initiation. 6. Change from the baseline of weight, BMI, blood pressure, pulse rate, laboratory test values, and NYHA class at the 4 and 8 weeks following protocol treatment initiation. 7. Change from the baseline of cardiac function indicators (LVEDV, LVESV, LVEF, septal e', lateral e', mitral orifice blood flow velocity waveform (E), E/e', LVMI, LAVI , LVOT, LVOT-VTI, TR velocity, IVC, GLS, Left atrial strain (2-chamber view and 4-chamber view), at 8 weeks following protocol treatment initiation, along with the percentage of cases with a respiratory variation of 50% or more in IVC. 8. Change from the baseline of KCCQ-12 at 8 weeks following protocol treatment initiation, along with the percentage of cases whose score increased by 5 points or more. 9. Time to composite outcomes of first HF event [the following events due to the exacerbation of HF: i) unplanned rehospitalization ii) initiation of intravenous treatment for HF (vasodilators, inotropes) (during hospitalization: excluding rehospitalization); iii) emergency visit for HF requiring intravenous therapy (vasodilators, inotropes, diuretics); and iv) initiation of oral diuretics (loop diuretics, thiazide diuretics, tolvaptan) or a dose ;increase of ≥50% (outpatient)] and all-cause mortality. 10. Number and frequency of composite outcomes of HF events including recurrence and all-cause mortality. 11. Number and frequency of following individual events: first or recurrent HF events, all-cause mortality, and cardiovascular death. 12. Number of occurrences and time to onset of specific adverse events, such as worsening of renal function (more than 50% increase in serum Cr or more than 30% decrease in eGFR), hyperkalemia (serum potassium 5.5 mEq/L or more), symptomatic hypotension, and angioedema. 13. Number of occurrences of other serious adverse events.   Safety endpoint:  Adverse events that occurred following the protocol treatment initiation |
| Subjects | Patients hospitalized due to worsening HF with HF symptoms and signs of congestion |
| Selection criteria  and exclusion criteria | Selection criteria  Patients who meet all of the following criteria shall be the subjects:   1. Patients who have given their written consent to participate in this study. 2. Patients aged 20 years or older at the time of consent (regardless of gender). 3. Patients hospitalized due to worsening HFe accompanied by HF symptoms (such as dyspnea at rest or with light exertion) and signs of congestion (such as edema, moist rales, and congestion on chest X-ray) (any level of left ventricular ejection fraction). 4. NYHA classification II-IV. 5. Patients receiving ACE inhibitors or ARBs. 6. Patients available for randomization within 7 days of current hospitalization. 7. Patients who meet the following criteria for hemodynamic stability. 8. Has a systolic blood pressure of 100 mm Hg or higher. 9. Have not increased the IV diuretic dose within 6 hours prior to randomization. 10. Have not been administered vasodilators (e.g., carperitide, nitrates) or inotropes intravenously. 11. Patients whose natriuretic peptides from 48 hours prior to current hospitalization to the time of eligibility determination meet the following criteria:   NT-proBNP ≥1200 pg/mL or BNP ≥300 pg/mL  Exclusion criteria:  Patients who meet any of the following criteria will be excluded from this study:   1. Patients who are currently taking sacubitril/valsartan or have taken it within 30 days prior to randomization. 2. Patients with a history of hypersensitivity to sacubitril/valsartan, ACE inhibitors, or ARB components, or who are expected to have contraindications to or intolerance to these drugs. 3. Patients with a history of angioedema. 4. Patients with severe renal dysfunction (eGFR less than 30 mL/min/1.73m), maintenance dialysis patients, patients with known bilateral renal artery stenosis (known renal artery stenosis in the residual kidney in patients with one kidney) 5. Patients with severe liver dysfunction (Child-Pugh classification C). 6. Diabetic patients receiving aliskiren fumar hydrochloride. 7. Patients with serum potassium level of 5.3 mEq/L or higher. 8. Patients with cardiogenic shock. 9. Patients using cardiopulmonary support devices, left ventricular assist devices, or ventilators. 10. Patients who developed acute coronary syndrome or stroke within 30 days prior to randomization. 11. Patients with a history of surgical or percutaneous treatment for cardiovascular disease within 30 days prior to randomization. 12. Patients with pre-planned coronary artery reconstruction, surgical or percutaneous treatment for cardiovascular disease, during the individual observation period. 13. Patients with pre-planned treatment such as electrical cardioversion, cardiac resynchronization therapy, or pacemaker implantation, during the individual observation period. 14. Patients with a history of or who are complicated with obstructive hypertrophic cardiomyopathy or infiltrative cardiomyopathy such as amyloidosis or sarcoidosis. 15. Patients with active pericardial disease. 16. Patients with a history of heart transplantation or those waiting for heart transplantation. 17. Patients with active infectious disease or severe chronic respiratory disease. 18. Patients who are pregnant, may become pregnant, or are breastfeeding. 19. Other patients considered unsuitable for this study by the representative investigator or sub-investigator (such as patients complicated with active malignant tumors). |
| Study period | Case registration period: Clinical study implementation plan/study summary publication system (jRCT) release date  - Mar. 31, 2023  Observation period: 8 weeks  Study implementation period: Clinical study implementation plan/study summary publication system (jRCT) release date  to March 31, 2025 |
| Target number of cases | 400 cases (sacubitril/valsartan treatment group: 200 cases, control group: 200 cases) |
| Study method | Upon confirming the eligibility of patients who have provided their consent, all patients who meet the eligibility criteria will be enrolled. Patients will be randomized into two groups: a sacubitril/valsartan group or a control group (sacubitril/valsartan non-administered group) within 7 days of hospitalization, after protocol treatment initiation during hospitalization, within 48 hours of enrollment and allocation, followed by 8 weeks of observation and testing.  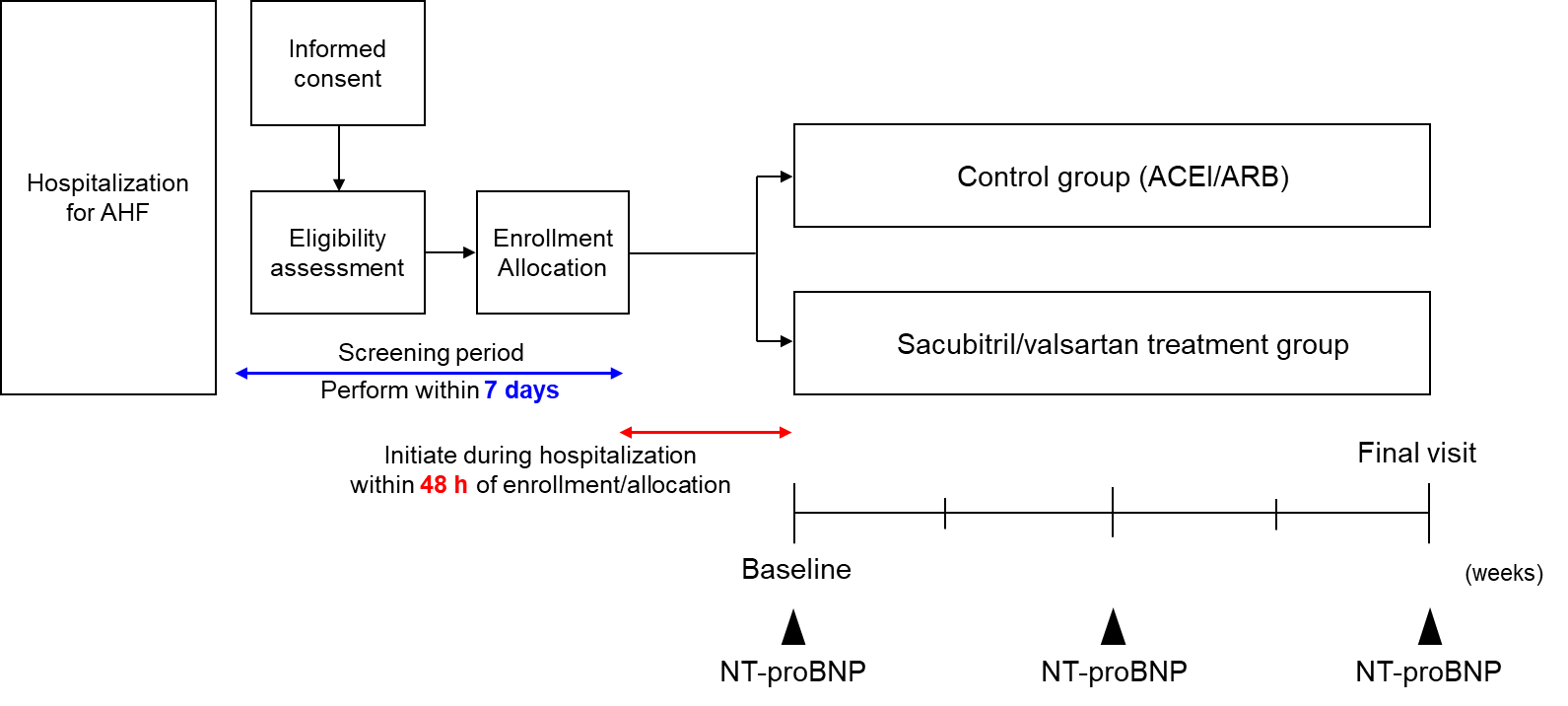 |

# **Background and purpose of the study**

## Study background

The number of patients with HF is increasing worldwide, including Japan, and is a major public health issue known as the 'HF pandemic.' The number of hospitalized patients due to HF at cardiovascular specialized facilities and training-related facilities, in Japan in 2015, was estimated to be approximately 240,000^1)^, with deaths from HF being the most common in the breakdown of heart disease, making it the second leading cause of death after malignant neoplasms among the total mortality count by cause of death^2)^. Over the course of HF, HF patients are characterized by repeated hospitalizations due to acute HF or acute exacerbation of chronic HF after a certain stage of the disease, resulting in a significantly worsened life prognosis^3)^. In particular, it has long been known that rehospitalization and increased mortality rates for HF patients tend to occur relatively early, within several months after discharge^4.5.6)^. However, while treatments for chronic stage HFrEF have been established based on the results of clinical trials to date, treatment for acute HF has focused on empiric symptomatic therapy aimed at reducing congestion and alleviating HF symptoms, so no fundamental treatment has been established. Furthermore, because no drastic treatment methods have been established to bridge the gap between acute HF and chronic HF, preventing rehospitalization before and after discharge from the hospital, and improving life prognosis, there is a strong need for progress in research and the establishment of treatments^7.8.9)^.

Sacubitril/valsartan is a novel drug for HF treatment that is expected to suppress the degradation of natriuretic peptides through inhibition of neprilysin, a type of proteolytic enzyme, while at the same time having cardioprotective effects through a complex pathway by inhibiting angiotensin II type I receptors. This drug has been widely used for HF (HFrEF) in Europe and the United States since 2015, as a result of multiple clinical trials verifying its therapeutic effect on chronic HF^10.11.12.13.14.15)^. In recent years, it has become clear that it has a certain therapeutic effect on HF with preserved ejection fraction (HFpEF) and it is an oral HF treatment drug that was approved in Japan in 2020. To date, clinical trials using this drug have mainly focused on patients with chronic HF. However, the PIONEER-HF trial conducted in the United States among HFrEF patients hospitalized due to worsening HF, reported in 2018, showed significantly reduced risk of HF rehospitalization or cardiovascular death at 8 weeks following administration, as a result of initiating sacubitril/valsartan during that hospitalization, while at the same time, significantly lowered NT-proBNP at 4 and 8 weeks following administration^16)^ compared to enalapril, a control drug^17)^ Because NT-proBNP is a blood biomarker that is widely used in the diagnosis, pathological evaluation, and treatment effect determination of HF patients, the results of said trial suggested that sacubitril/valsartan is highly effective for HFrEF patients after hospitalization due to worsening HF. On the other hand, a clinical trial (NCT03988634) to similarly evaluate the effect on NT-proBNP among HFpEF patients hospitalized due to worsening HF is currently being conducted in the United States as of February 2021. Therefore, although the effects of sacubitril/valsartan are still unknown for the same group,^18)^ a sub-analysis of the PARGON-HF study targeting HFpEF patients with chronic HF showed that sacubitril/valsartan was more effective in reducing HF rehospitalization or cardiovascular death when patients with a HF hospitalization history of less than 30 days initiated sacubitril/valsartan, compared to patients with a HF hospitalization history of more than 30 days or no history of HF hospitalization^19).^ These results indicate that early initiation of sacubitril/valsartan for patients hospitalized due to worsening HF, regardless of left ventricular ejection fraction, is a powerful treatment option to improve the prognosis after discharge.

Most of the clinical trials mentioned above were conducted in Europe and the United States, so there are many unclear points as to whether the results obtained can be immediately applied to clinical practices in Japan. In particular, in these clinical trials, there were many participants who were obese or relatively young, which is partially out of line with the actual state of HF treatment in Japan, where there are many non-obese patients and elderly people. Furthermore, using sacubitril/valsartan requires switching from ACE inhibitors or ARBs in Japan, but only about half of the patients had switched from these drugs in the PIONEER-HF trial^16).^ Furthermore, even if the subjects have the same illness, a HF, there are various differences between Europe, the United States, and Japan, including differences in pathophysiology due to racial differences and differences in the medical treatment system for acute HF. Therefore, independently verifying the safety and effectiveness of sacubitril/valsartan for patients hospitalized due to worsening HF, through conducting clinical trials in line with actual clinical practice in Japan, is expected to lead to the improvement of the quality of medical care in this field.

## Study purpose

To examine the effects of sacubitril/valsartan compared with the standard treatment for hospitalized patients with worsening heart failure (HF), using changes in NT-proBNP as an indicator.

# **Study contents**

## Study endpoints

1) Primary endpoin:

Between-group ratio of the proportional change in the geometric mean NT-proBNP at 8 weeks following protocol treatment initiation, compared with baseline

Setting basis:

NT-proBNP is an established blood biomarker that is widely used to determine the efficacy of treatment for HF, in addition to also having been used as an efficacy evaluation item in similar clinical trials conducted in Europe and the United States using the same drug.

2) Secondary endpoint:

1. Between-group ratio of the proportional change in geometric mean NT-proBNP at 4 weeks following protocol treatment initiation, compared with baseline.
2. Percentage of cases in which the NT-proBNP value decreased by 50% or more from the baseline, at 8 weeks following protocol treatment initiation.
3. Percentage of cases in which the NT-proBNP value decreased by 30% or more from the baseline, at 4 weeks following protocol treatment initiation.
4. Percentage of cases which mean NT-proBNP value decreased by 40% or more from the baseline at 4 and 8 weeks following protocol treatment initiation.
5. Change from the baseline of cardiac troponin T, CRP, GDF-15, soluble ST2, glycoalbumin, and 1.5AG at 8 weeks after following protocol treatment initiation.
6. Change from the baseline of weight, BMI, blood pressure, pulse rate, laboratory test values, and NYHA class at the 4 and 8 weeks following protocol treatment initiation.
7. Change from the baseline of cardiac function indicators (LVEDV, LVESV, LVEF, septal e', lateral e', mitral orifice blood flow velocity waveform (E), E/e', LVMI, LAVI , LVOT, LVOT-VTI, TR velocity, IVC, GLS, Left atrial strain (2-chamber view and 4-chamber view), at 8 weeks following protocol treatment initiation, along with the percentage of cases with a respiratory variation of 50% or more in IVC.
8. Change from the baseline of KCCQ-12 at 8 weeks following protocol treatment initiation, along with the percentage of cases whose score increased by 5 points or more.
9. Time to composite outcomes of first HF event [the following events due to the exacerbation of HF: i) unplanned rehospitalization ii) initiation of intravenous treatment for HF (vasodilators, inotropes) (during hospitalization: excluding rehospitalization); iii) emergency visit for HF requiring intravenous therapy (vasodilators, inotropes, diuretics); and iv) initiation of oral diuretics (loop diuretics, thiazide diuretics, tolvaptan) or a dose ;increase of ≥50% (outpatient)] and all-cause mortality.
10. Number and frequency of composite outcomes of HF events including recurrence and all-cause mortality.
11. Number and frequency of following individual events: first or recurrent HF events, all-cause mortality, and cardiovascular death*.
12. Number of occurrences and time to onset of specific adverse events, such as worsening of renal function (more than 50% increase in serum Cr or more than 30% decrease in eGFR), hyperkalemia (serum potassium 5.5 mEq/L or more), symptomatic hypotension, and angioedema.
13. Number of occurrences of other serious adverse events.

*Cardiovascular death is defined as a case in which the main cause of death is determined to be atherosclerotic cardiovascular disease, HF, or sudden cardiac death (including cases in which death is not clearly caused by non-cardiovascular disease).

Setting basis:

It was set to complement the primary endpoint regarding the effect of sacubitril/valsartan on NT-proBNP (1-4). The aim was to evaluate the clinical effects of sacubitril/valsartan on the relevant markers associated with HF (5-8). The aim was to evaluate the impact on HF-related events and all-cause mortality (9-11). The aim was to evaluate the safety related to sacubitril/valsartan and this clinical study (12-13).

3) Safety endpoint

Adverse events that occurred following the protocol treatment initiation

Setting basis:

It was set to evaluate the safety of sacubitril/valsartan.

## Study method (type/design of clinical research)

Investigator-initiated, multicenter, prospective, parallel-group, randomized controlled trial

## Study method (clinical research procedures)

Upon confirming the eligibility of patients who have provided their consent, all patients who meet the eligibility criteria will be enrolled. Patients will be randomized into two groups: a sacubitril/valsartan group or a control group (sacubitril/valsartan non-administered group) within 7 days of hospitalization, after protocol treatment initiation during hospitalization, within 48 hours of enrollment and allocation, followed by 8 weeks of observation and testing. The protocol treatment period for the sacubitril/valsartan group starts with the administration of sacubitril/valsartan the day after the baseline NT-proBNP is collected and goes until 8 weeks thereafter, while for the control group, the protocol treatment period starts from the day after the baseline NT-proBNP blood collection and goes until 8 weeks thereafter. Following the protocol treatment initiation, participants will be transferred to outpatient treatment depending on their condition, with follow-ups conducted until 8 weeks thereafter.

The HF treatment in this study shall be performed, in principle, within the scope of insurance coverage in accordance with the latest guidelines for the treatment of acute and chronic HF by the Japanese Circulation Society. At the same time, there will be no restrictions on the use of drugs other than the study drug (HF drugs including diuretics and drugs for other comorbid conditions) based on the comprehensive clinical judgment of the doctor in charge, including the medical condition of the study participants. Additionally, during the protocol treatment period, while the usage and dosage of these drugs shall not be changed in principle, changes shall be allowed at the discretion of the doctor in charge depending on the medical condition of the study participants.


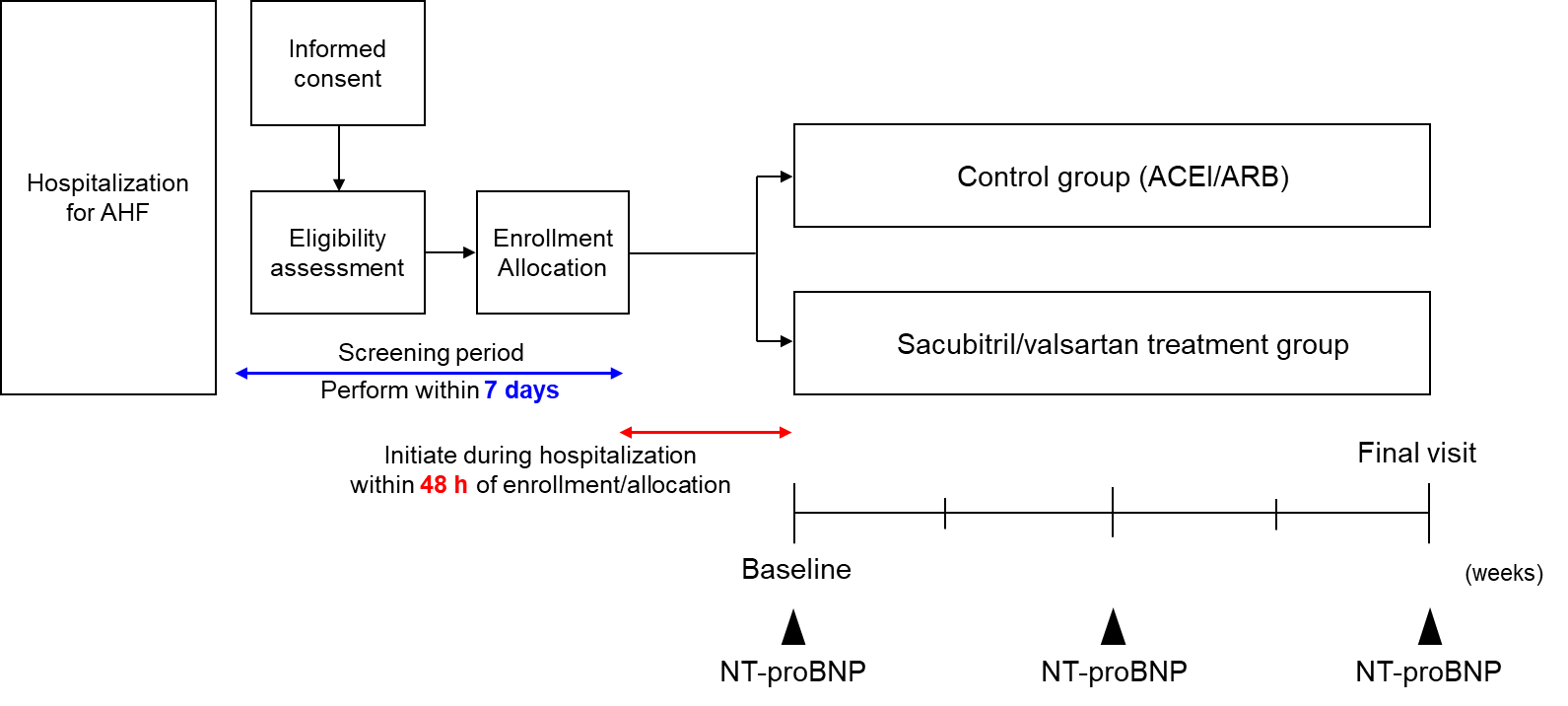


## Administration method

### Administration method for the sacubitril/valsartan group

Switch from the ACE inhibitor or ARB that was taken before the allocation, and start oral administration twice daily with a starting dose of 24/26 mg of sacubitril/valsartan. The duration of administration of the ACE inhibitor or ARB before allocation does not matter, but when switching from the ACE inhibitor, administration of sacubitril/valsartan should be started at least 36 hours after the final administration of the drug.

After the start of administration, the dose of sacubitril/valsartan is gradually increased to 49/51 mg and 97/103 mg twice daily at intervals of 2 to 4 weeks, referring to the following latest package insert and safety and tolerability standards. At that time, if the doctor in charge determines that the dose is not tolerated after the dose is increased, the dose may be reduced to the previous dose or the drug may be suspended depending on the medical situation, the details of which will be recorded in the CRF.

**Dose adjustment of sacubitril/valsartan**


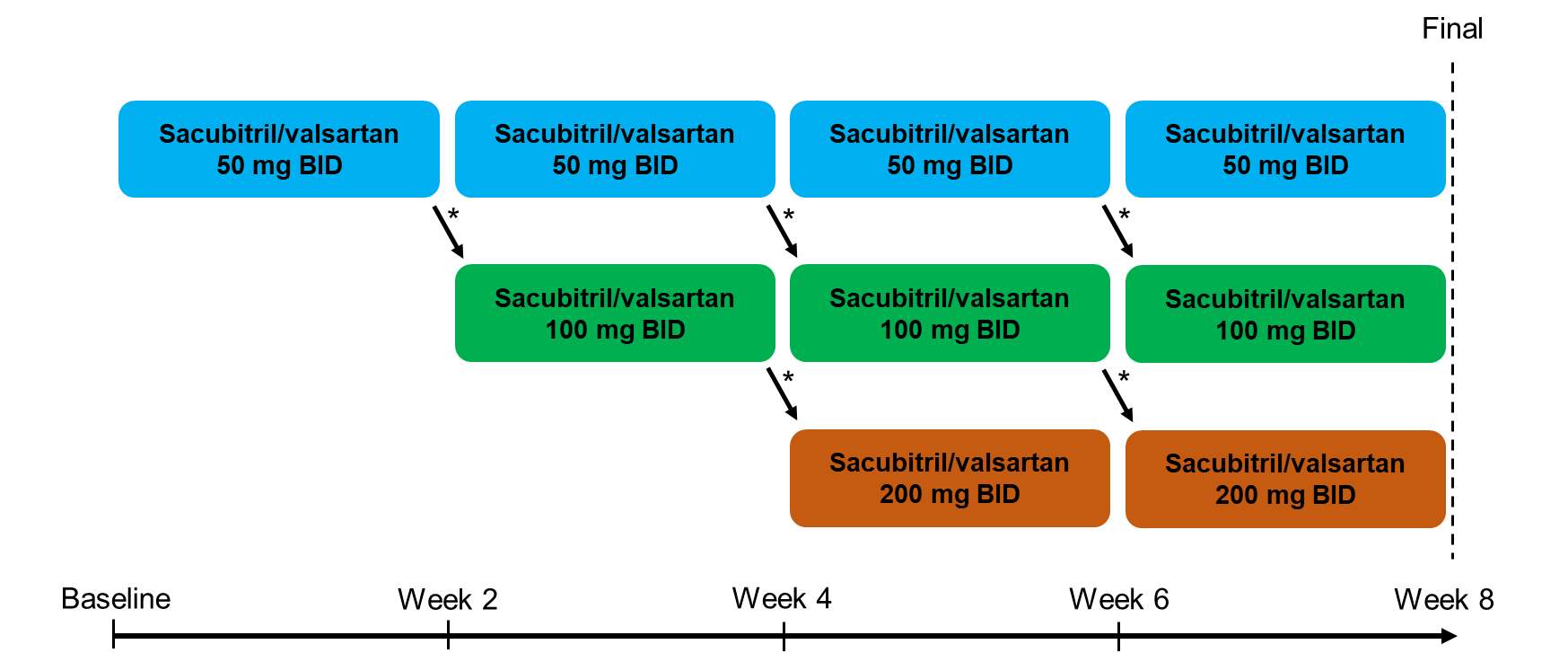


*The safety and tolerability of sacubitril/valsartan will be evaluated based on the following criteria and if no medical problems are observed, the dose of sacubitril/valsartan will be gradually increased to the next dose.

i) Has no symptomatic hypotension and the systolic blood pressure is 95 mmHg or higher

ii) Serum potassium is 5.4 mEq/L or less

iii) eGFR is 30 mL/min/1.73 m^2^ or more

iv) Most recent eGFR decline rate is 35% or less

The timing of increasing the dose of sacubitril/valsartan is not limited to weeks 2, 4 , and 8 time points indicated in the figure.

### Criteria for discontinuation of sacubitril/valsartan

Discontinue administration of sacubitril/valsartan if any of the following applies:

1. When the representative investigator or sub-investigator determines that it is difficult to continue sacubitril/valsartan due to the occurrence of adverse events
2. When the representative investigator or sub-investigator determines that it is appropriate to discontinue administration for other reasons

### Administration method for the control group (sacubitril/valsartan non-administration group)

Standard treatment for HF including ACE inhibitors, ARBs, and drugs other than sacubitril/valsartan that patients were taking prior to allocation will be carried out.

## Discontinuation criteria for the entire study

If information corresponding to the following items is obtained, the principal investigator will consider whether or not to continue conducting the study. If the representative investigator discontinues the study, he/she will promptly notify the study participants of this discontinuation, provide appropriate medical care, and take other necessary measures. If the representative investigator discontinues the study, he/she shall report this discontinuation, the reasons for discontinuation, and a summary of the results in writing to the administrator of the participating medical institution without delay.

1. When the CRB determines that the study should not be continued
2. When any doubt occurrs regarding the safety of the study
3. When facts or information undermining the ethical validity or scientific validity of the study are obtained
4. When information or facts undermining the appropriateness of the study implementation or the credibility of the results are obtained

### 2.5.1 Cancellation/termination procedures

If the principal investigator wishes to discontinue the entire study, he/she shall state this fact and reasons therefore in the "Notice of Discontinuation (Uniform Form 11)" and submit it to the CRB within 10 days from the date of discontinuation. The cancellation letter shall indicate whether there are any study participants who require observation.

If the principal research physician decides to discontinue the study, he/she will report the discontinuation of the study and the reasons therefore in writing to the representative investigator at each medical institution. The representative investigator shall promptly inform the study participants during the study period and take steps such as changing to an appropriate treatment. The principal research physician will, as necessary, seek the opinion from the CRB regarding the timing and method of completing the study, in accordance with the measures taken for the study participants. Additionally, even if a notice of discontinuation is submitted, disease reports and periodic reports shall be made until the study is completed. If changes to the implementation plan are necessary, notification of changes to the implementation plan shall be made and submitted.

When the principal research physician has submitted a notice of discontinuation and has completed the measures for the study participants, a comprehensive report shall be submitted, in principle, within one year from whichever date is later, either the discontinuation date or the end of the period for collecting data related to all study endpoints.

## Randomization procedure

Randomization of study participants will be performed using a central registration method. Upon confirming the eligibility of study participants, the doctor in charge will immediately access the study website (EDC) and enter the information necessary for registration. The information will be immediately confirmed on the EDC website, and if eligible, a case registration number will be assigned and protocol treatment allocated on the EDC.

Allocation to the sacubitril/valsartan group and the control group will be made at a 1:1 ratio using the following allocation adjustment factors.

[Allocation factors]

Age upon obtaining consent (under 70, over70)

Sex (Female, Male)

LVEF (less than 40%, more than 40%)*

Atrial fibrillation (presence or absence)

eGFR (less than 60 mL/min/1.73 m^2^, more than 60 mL/min/1.73 m^2^)

Facility

Priority will be given to data obtained during the current hospitalization; however, if data cannot be obtained, data from within 3 months prior to obtaining consent may also be used.

## Original source materials

Original source materials refer to the original records of drug administration information and clinical findings, observations, and other activities for research participants, obtained through medical treatment, proving that the patient and the data collected from that patient are real. In this study, original source materials include medical records, nursing records, prescription records, consent forms, clinical test data, and the contents of the clinical research agreement concluded between Novartis Pharma Co., Ltd. and Saga University. The original source documents shall be stored at each participating medical institution.

## Handling of case report forms

1. In this study, data will be collected using EDC, with the data collected referred to as a case report form (CRF). Regarding the examination results of blood biomarkers, which are centrally measured, all data will be input separately at the data center. The study office will send the results of the centralized measurement to the data center, where they will be input into an Excel file. The data is separately entered by two individuals, after which, upon checking the numbers to ensure a complete match, the information is sent to the representative investigator at each medical institution for approval.

2) The representative investigator and sub-investigator or research collaborators will enter the data into the EDC. Research collaborators can input their own information into the EDC from the original source materials, as long as the original source materials exist and their objectivity can be guaranteed.

3) After the first input data is saved on the server, all modification history (modification date and time, person who modified it, data before and after modification, reason for modification) is recorded in the system.

4) The representative investigator will confirm the accuracy of the data once all data has been entered into the EDC.

## CRF content confirmation and inquiries

1. The data center will check the following items regarding the entered CRF.
2. Incomplete input
3. Consistency with this study plan
4. Consistency of CRF contents
5. Blood biomarker outliers
6. The data center will summarize the points to be inquired and send a data inquiry form to the representative investigator, etc. by e-mail.
7. The representative investigator, sub-investigator, or research collaborator will promptly enter or modify the CRF on the website. If the inquiry does not require correction, the data center will be notified to that effect.

# **Inclusion criteria**

## Subjects

Patients hospitalized due to worsening HF with HF symptoms and signs of congestion

## Selection criteria

Patients who meet all of the following criteria shall be the subjects:

1. Patients who have given their written consent to participate in this study.
2. Patients aged 20 years or older at the time of consent (regardless of gender).
3. Patients hospitalized due to worsening HFe accompanied by HF symptoms (such as dyspnea at rest or with light exertion) and signs of congestion (such as edema, moist rales, and congestion on chest X-ray) (any level of left ventricular ejection fraction).
4. NYHA classification II-IV.
5. Patients receiving ACE inhibitors or ARBs.
6. Patients available for randomization within 7 days of current hospitalization.
7. Patients who meet the following criteria for hemodynamic stability.
8. Has a systolic blood pressure of 100 mm Hg or higher.
9. Have not increased the IV diuretic dose within 6 hours prior to randomization.
10. Have not been administered vasodilators (e.g., carperitide, nitrates) or inotropes intravenously.
11. Patients whose natriuretic peptides from 48 hours prior to current hospitalization to the time of eligibility determination meet the following criteria:

NT-proBNP ≥1200 pg/mL or BNP ≥300 pg/mL

[Basis for setting the selection criteria]

1. It was set to comply with the Clinical Trials Act.
2. It was set to target adults from whom we can obtain consent.

3)-6), 8) They were set to select cases that meet the purpose of this study.

7) It was set to ensure patient safety.

## Exclusion criteria

Patients who meet any of the following criteria will be excluded from this study:

1. Patients who are currently taking sacubitril/valsartan or have taken it within 30 days prior to randomization
2. Patients with a history of hypersensitivity to sacubitril/valsartan, ACE inhibitors, or ARB components, or who are expected to have contraindications to or intolerance to these drugs.
3. Patients with a history of angioedema
4. Patients with severe renal impairment (eGFR < 30 mL/min/1.73m^2),^ patients on maintenance dialysis, and patients with known bilateral renal artery stenosis (known renal artery stenosis in the remaining kidney in patients with one kidney)
5. Patients with severe liver dysfunction (Child-Pugh classification C)
6. Diabetic patients receiving aliskiren fumar hydrochloride
7. Patients with serum potassium level of 5.3 mEq/L or higher
8. Patients with cardiogenic shock
9. Patients using cardiopulmonary support devices, left ventricular assist devices, or ventilators
10. Patients who developed acute coronary syndrome or stroke within 30 days prior to randomization
11. Patients with a history of surgical or percutaneous treatment for cardiovascular disease within 30 days prior to randomization
12. Patients with pre-planned coronary artery reconstruction, surgical or percutaneous treatment for cardiovascular disease, during the individual observation period
13. Patients with pre-planned treatment such as electrical cardioversion, cardiac resynchronization therapy, or pacemaker implantation, during the individual observation period.
14. Patients with a history of or who are complicated with obstructive hypertrophic cardiomyopathy or infiltrative cardiomyopathy such as amyloidosis or sarcoidosis.
15. Patients with active pericardial disease
16. Patients with a history of heart transplantation or those waiting for heart transplantation
17. Patients with active infectious disease or severe chronic respiratory disease
18. Patients who are pregnant, may become pregnant, or are breastfeeding
19. Other patients considered unsuitable for this study by the representative investigator or sub-investigator (such as patients complicated with active malignant tumors)

[Basis for setting the exclusion criteria]

1), 12) to 15) They were set because they are expected to have an impact on the study endpoints.

2)-10), 17), 18) They were set to ensure patient safety.

11), 16) They were set because they are expected to have an impact on ensuring patient safety and on the study endpoints.

19) From scientific and ethical aspects, they were set assuming that the doctor in charge would decide whether or not the subject is appropriate.

## Discontinuation criteria for each study participant

The study will be discontinued in the following cases. If the study is discontinued, the reason and date of discontinuation will be entered in the CRF, and if the discontinuation date falls within the 4-week and 8-week allowance, an investigation will be conducted to the extent possible.

1. When a study participant requests to decline participation in the study or withdraws their consent.
2. When it becomes difficult to visit the medical institution implementing the study due to relocation or hospital transfer.
3. When it is found that the selection criteria are not met following the initiation of the study or that the exclusion criteria have been violated.
4. When the representative investigator or sub-investigator determines that it is difficult for the study participant to continue the protocol treatment due to the occurrence of an adverse event.
5. When the representative investigator of sub-investigator determines that it is difficult to continue the research due to deterioration of the underlying disease or worsening of complications.
6. When the representative investigator of sub-investigator deems it appropriate to discontinue participation in the study for other reasons.

## Handling of study participants who discontinue protocol treatment

Regarding study participants who discontinue protocol treatment, a test will be conducted at the time of discontinuation to the extent possible and their participation in the study will be discontinued, except for those study participants who fall under 1 to 4 below. Regarding the investigation schedule, refer to the relevant section of "Observation/Examination Items." Even if it is not possible to conduct the observations specified in the study plan due to hospital transfer, etc., obtain outcome information from the transferred hospital to the extent possible and record the obtained information in medical records such as medical charts, along with the method and date of acquisition.

1. In the event the study participant dies
2. In the event the study participant withdraws their consent for post-discontinuation follow-up
3. In the event the study participant is found to be ineligible after protocol treatment initiation
4. In the event tracking is difficult for other reasons

# **Treatment for study subjects**

## Observation/examination schedule

The observation, examination, and investigation items and schedule for this study are shown below. The representative investigator, etc., will conduct observations, examinations, and investigations in accordance with the schedule. Items that can be carried out by research collaborators, such as investigation of patient backgrounds and clinical tests, may be carried out by research collaborators under the supervision of the representative investigator or sub-investigator.

Table 1 Observation/examination schedule

| Timing  Investigation item | When obtaining consent^1)^ | At the time of eligibility determination,  registration/allocation^1)^ | Observation period | | |
| --- | --- | --- | --- | --- | --- |
|  |  |  | Baseline | 4 weeks^2)^ | 8 weeks^2)^ |
|  |  |  | Day 0 | Day 28  [21-35] | Day 56  [42-70] |
| Obtaining consent | ● |  |  |  |  |
| Eligibility determination, registration/allocation^3)^ |  | ● |  |  |  |
| Patient background information^4)^ |  | ● |  |  |  |
| Physical findings^5)^ |  |  | ● | ● | ● |
| NYHA classification |  |  | ● | ● | ● |
| NT-proBNP |  |  | ● | ● | ● |
| Presence of atrial fibrillation, atrial flutter, or other atrial tachycardia^6)^ |  |  | ● | ● | ● |
| Blood biomarkers^7)^ |  |  | ● |  | ● |
| Blood test^8)^ |  |  | ● | ● | ● |
| Cardiac ultrasound examination^9)^ |  |  | ●^10)^ |  | ● |
| KCCQ-12 ^9)^ |  |  | ●^10)^ |  | ● |
| Concomitant drugs/therapy^11)^ |  |  | ● |  | ● |
| Administration status of sacubitril/valsartan^12)^ |  |  |  |  |  |
| Administration status of control group |  |  |  |  |  |
| Adverse events |  |  |  |  |  |
| HF events and  cardiovascular death |  |  |  |  |  |

[ - ]: Tolerance

1) Obtain consent, determine eligibility, and perform registration/allocation within 7 days of admission.

2) If the discontinuation date falls within the 4 and 8 weeks allowance, an investigation will be conducted to the extent possible.

3) Regarding the LVEF allocation factor, priority is given to data obtained during the current hospitalization; however, if data cannot be obtained, data from within 3 months prior to obtaining consent may be used.

4) Regarding height, priority will be given to data obtained during the current hospitalization; however, if data cannot be obtained, data from within 12 months prior to obtaining consent may be used.

5) Weight, BMI, blood pressure, and pulse rate.

6) Confirm the presence of NT-proBNP at the time of blood collection.

7) Cardiac troponin T, CRP, GDF-15, soluble ST2, glycoalbumin, 1.5AG.

8) HbA1c is optional (diabetic cases, etc.).

9) Implement at a medical institution where implementation is available.

10) Data from consent acquisition to baseline will be made available.

11) New administration of SGLT2 inhibitors and MRA, along with changes in the dosage and administration of concomitant drugs containing them, will not be implemented, in principle.

12) Initiate oral administration twice daily within 48 hours of registration/allocation. Oral administration will start twice daily from the day after baseline.

## Patient registration

1. The representative investigator or sub-investigator will obtain written consent from the patient, determine eligibility through tests and interviews necessary to confirm eligibility, and register and allocate the patient within 7 days of admission.
2. Based on the results of eligibility determination, enter eligibility and allocation survey items in EDC and carry out registration and allocation. Registered study participants (including those determined to be ineligible by the EDC) will be given a patient identification number and the group to which they have been allocated.
3. An "anonymization correspondence table" will be created at the implementing medical institution and the numbers will be written down to prevent patient mix-ups, with the representative investigator, sub-investigator, or research collaborator keeping the original copy at the medical institution.

### Investigation items at the time of registration/allocation

After the time of eligibility determination, record the below upon registration/allocation, and enter into the EDC.

・Date of obtaining consent, age when obtaining consent, date of birth, sex

・Presence or absence of atrial fibrillation, LVEF*, eGFR

*Priority will be given to data obtained during the current hospitalization; however, if data cannot be obtained, data obtained within 3 months prior to obtaining consent may be used.

### Patient background information

Date of hospitalization, height*, weight, BMI (automatically calculated), blood pressure (systolic/diastolic), pulse rate, presence of complications/pre-existing conditions (hypertension, diabetes, dyslipidemia, ischemic heart disease, ischemic Stroke, atrial fibrillation, HF [present: <1.5 years or >1.5 years], HF hospitalization, COPD), cause of HF (ischemic, non-ischemic), NYHA classification (II-IV), LVEF (<40%, ≥40%)**, concomitant medications (HF drugs/antihypertensive drugs [ACE inhibitors or ARBs, β-blockers, calcium channel blockers, MRA, diuretics, digitalis preparations], SGLT2 inhibitors, ivabradine], statins), (existing) combination therapy (PCI, CABG, pacemaker, ICD, CRT)

*For height, priority will be given to data obtained during the current hospitalization; however, if data cannot be obtained, data obtained within 12 months prior to obtaining consent may also be used.

**For LVEF, priority will be given to data obtained during the current hospitalization; however, if data cannot be obtained, data from within 3 months prior to obtaining consent may also be used.

## Observation/examination items for prescribed visits

1. Baseline (Day 0: During hospitalization)
2. Physical findings: weight, BMI (automatically calculated), blood pressure (systolic/diastolic), and pulse rate
3. NYHA classification: Ⅰ-IV
4. NT-proBNP
5. Presence or absence of atrial fibrillation, atrial flutter, or other atrial tachycardia
6. Blood biomarkers:

Cardiac troponin T, CRP, GDF-15, soluble ST2, glycoalbumin, 1.5AG

1. Blood test:

RBC, Hb, Ht, PLT, HbA1c*, fasting blood glucose, TC, HDL-C, non-HDL-C (automatically calculated), TG, AST, ALT, γ-GTP, uric acid, serum Cr, eGFR (automatically calculated), sodium, potassium, FIB-4 index (calculated), ePV (calculated by Kaplan-Hakim formula)

*Optional (diabetic cases, etc.)

1. Echocardiography (Optional): (Data from consent acquisition to baseline can be used)

LVEDV, LVESV, LVEF, septal e’, lateral e’, mitral orifice blood flow velocity waveform (E), E/e’, LVMI, LAVI, LVOT, LVOT-VTI, TR velocity, IVC, GLS*, left atrial strain (2-chamber view and 4-chamber view)*

*DICOM video will be recorded and evaluated at the facility in charge of analysis.

For details, refer to Attachment 2) Cardiac Ultrasound Test Measurement Manual.

1. KCCQ-12 (optional): (data from consent acquisition to baseline can be used)
2. Concomitant drugs/combination therapy
3. 4 weeks (Day 28, observation/examination tolerance range: Day 21-35)
4. Physical findings: weight, BMI (automatically calculated), blood pressure (systolic/diastolic), and pulse rate
5. NYHA classification: I-IV
6. NT-proBNP
7. Presence or absence of atrial fibrillation, atrial flutter, or other atrial tachycardia
8. Blood test:

RBC, Hb, Ht, PLT, HbA1c*, fasting blood sugar, AST, ALT, γ-GTP, uric acid, serum Cr, eGFR (automatically calculated), sodium, potassium, FIB-4 index (calculated), ePV (calculated using the Strauss formula)

*Optional (diabetic cases, etc.)

1. 8 weeks (Day 56, observation/examination tolerance range: Day 42-70)
2. Physical findings: weight, BMI (automatically calculated), blood pressure (systolic/diastolic), and pulse rate
3. NYHA classification: I-IV
4. NT-proBNP
5. Presence or absence of atrial fibrillation, atrial flutter, or other atrial tachycardia
6. Blood biomarkers:

Cardiac troponin T, CRP, GDF-15, soluble ST2, glycoalbumin, 1.5AG

1. Blood test:

RBC, Hb, Ht, PLT, HbA1c*, fasting blood glucose, TC, HDL-C, non-HDL-C (automatically calculated), TG, AST, ALT, γ-GTP, uric acid, serum Cr, eGFR (automatically calculated), sodium, potassium, FIB-4 index (calculated), ePV (calculated using the Strauss formula)

*Optional (diabetic cases, etc.)

1. Echocardiography (optional):

LVEDV, LVESV, LVEF, septal e', lateral e', mitral orifice blood flow velocity waveform (E), E/e', LVMI, LAVI, LVOT, LVOT-VTI, TR velocity, IVC, GLS*, Left atrial strain (2-chamber view and 4-chamber view)*

*Record the DICOM video (optional) and evaluate it at the facility in charge of analysis.

For details, refer to Attachment 2) Cardiac Ultrasound Test Measurement Manual.

1. KCCQ-12 (optional):
2. Concomitant drugs/combination therapy

## Occasional observation/examination items

1. Sacubitril/valsartan medication status (from baseline up to 8 weeks):

Start date of administration, dose (mg/day), reason for discontinuation of administration and end date of administration, date of dose change (increase, decrease), changed dose, and reason therefor

1. Control group administration status (from baseline up to the 8 weeks):

Start date of administration, dose (mg/day), reason for discontinuation of administration and end date of administration, date of dose change (increase, decrease), changed dose, and reason therefor

1. Adverse events (up to 8 weeks from consent):

Event name, date of onset, severity (serious, non-serious), presence or absence of a causal relationship (if "yes", name of drug suspected of being related), outcome (recovered, remitted, not recovered, recovered but with sequelae, death, unknown), and date of outcome confirmation

1. HF events and cardiovascular death (from baseline up to 8 weeks) as defined below: Date and history of onset. Additionally, if the drug was changed due to worsening HF, the details of the change.

・Definition of HF event

i) Unplanned rehospitalization, ii) Start of intravenous treatment (vasodilators, inotropes) for HF (during hospitalization: excluding rehospitalization), iii) Emergency visit for HF requiring intravenous treatment (vasodilators, inotropes, diuretics), iv) Start of oral diuretics (loop diuretics, thiazide diuretics, tolvaptan) or increase in dose by 50% or more (outpatient)

・Definition of cardiovascular death

Cardiovascular death is defined as a case in which the main cause of death is determined to be atherosclerotic cardiovascular disease, HF, or sudden cardiac death (including cases in which death is not clearly caused by non-cardiovascular disease).

## Central measurement examination

NT-proBNP and blood biomarkers will be centrally measured at SRL Inc. SRL Inc. will collect and measure the anonymized samples.

Details of the procedures for sample collection, transportation, measurement, and provision of measurement results will follow the standard procedure separately established by SRL Inc.

Video data of echocardiography (optional item) will be evaluated at the Department of Cardiovascular Medicine, Tokushima University Hospital. For details, refer to Attachment 2) Cardiac Ultrasound Test Measurement Manual.

## Study drug overview

An overview of the study drug (sacubitril/valsartan) in this study (excerpted from the 2nd edition of the attached document (revised August 2020)) is provided below. For details, refer to the latest attached document.

4.6.1 Name and classification

Generic name: Sacubitril/valsartan sodium hydrate

Brand name: Enresto® Tablets

Manufacturer: Novartis Pharma K.K.

Partnership: Otsuka Pharmaceutical Co., Ltd.

Drug class name: Angiotensin receptor neprilysin inhibitor (ARNI)

Approval Number: 30200AMX00504000 (50 mg), 30200AMX00502000 (100 mg),

30200AMX00503000 (200 mg)

4.6.2 Efficacy/effects

Chronic HF

However, this is limited to patients undergoing standard treatment for chronic HF.

<Precautions related to efficacy/effects>

This drug should be administered by switching from an angiotensin-converting enzyme inhibitor or angiotensin II receptor antagonist.

4.6.3 Dosage and administration

The usual initial dose for adults is 50 mg of sacubitril/valsartan orally twice daily. If tolerated, incrementally increase the dose to 200 mg once at 2-4 week intervals. The single dose is 50 mg, 100 mg, or 200 mg, and all doses are administered orally twice daily. The dose should be reduced as appropriate depending on tolerability.

<Precautions related to dosage and administration>

1. For the following patients, carefully monitor the patient's condition and carefully decide whether or not to increase the dose.

・Patients with ‌renal dysfunction (less than eGFR 90 mL/min/1.73 m^2^)

・Patients with moderate liver dysfunction (Child-Pugh classification B)

・Patients with low blood pressure

1. When increasing the dose of this drug, consider the following criteria for blood pressure, serum potassium level, and renal function used in clinical trials.

Standards for dose increase* used in clinical trials

| Blood pressure | No symptomatic hypotension and systolic blood pressure is ≥95 mmHg |
| --- | --- |
| Serum potassium level | ≤ 5.4 mEq/L |
| Renal function | eGFR 30 mL/min/1.73 m^2^ or more and eGFR decline rate less than 35% |

*These are the criteria for increasing the dose from 50 mg at a time to 100 mg at a time, wherein, in clinical trials, patients who met all of the criteria were allowed to increase the dose.

1. Bioequivalence of 50 mg tablets and 100 mg or 200 mg tablets has not been demonstrated; therefore, 50 mg tablets should not be used when administering doses of 100 mg or higher.

4.6.4 Usage Precautions

- 1. Contraindications (Do not administer to the following patients)

1. Patients with a history of hypersensitivity to the components of this drug

2. Patients receiving or is within 36 hours of discontinuing administration of angiotensin-converting enzyme inhibitors (aracepril, imidapril hydrochloride, enalapril maleate, captopril, quinapril hydrochloride, cilazapril hydrate, temocapril hydrochloride, delapril hydrochloride, trandolapril, benazepril hydrochloride, perindopril erbumine, lisinopril hydrate)

3. Patients with a history of angioedema (angioedema caused by angiotensin II receptor antagonists or angiotensin converting enzyme inhibitors, hereditary angioedema, acquired angioedema, idiopathic angioedema, etc.)

4. Diabetic patients receiving aliskiren fumarate

5. Patients with severe liver dysfunction (Child-Pugh classification C)

6. Pregnant women or women who may be pregnant

1. Important basic precautions

1. If an angiotensin-converting enzyme inhibitor is being administered prior to administering this drug, it should be discontinued at least 36 hours prior to initiating administration of this drug, as angioedema may occur. In addition, if an angiotensin-converting enzyme inhibitor is administered following administration of this drug, do not administer it until 36 hours after the final administration of the drug.

2. Because there is a risk of symptomatic hypotension, the patient's condition should be carefully monitored and administered with care, particularly at the start of administration and when increasing the dose.

3. There have been reports of serious liver damage such as hepatitis occurring during administration of angiotensin II receptor antagonists, so patients should be carefully monitored by conducting liver function tests.

4. Because there is a risk of dehydration, patients should be carefully monitored and if any abnormalities are observed, appropriate measures such as reducing the dose of this drug, discontinuing administration, or replacing fluids should be taken.

5. It is preferable not to administer the drug within 24 hours prior to surgery. Hypotension may occur during anesthesia and surgery due to the inhibitory effect of the renin-angiotensin system.

6. Because dizziness and light-headedness may occur due to the hypotensive effect, caution should be taken when working at heights, driving a car, or operating dangerous machinery.

1. Interaction

Sacubitrilat and valsartan are substrates of OATP1B1 and OATP1B3. Note that sacubitril and sacubitrilat inhibit OATP1B1 and OATP1B3.

1. Contraindications for concomitant use (do not use together)

| Drug name etc. | Clinical symptoms/treating methods | Mechanism/risk factors |
| --- | --- | --- |
| Angiotensin converting  Enzyme inhibitor  Alacepril  (Cetapril)  Imidapril hydrochloride  (Tanatril)  Enalapril maleate  (Renivace)  Captopril  (Captril)  Quinapril hydrochloride  (Conan)  Cilazapril hydrate  (Inhibace)  Temocapril Hydrochloride  (Acecol)  Delapril hydrochloride  (Adecut)  Trandolapril  (Odric)  Benazepril  (Cibacen)  Perindopril erbumine  (Coversyl)  Lisinopril hydrate  (Zestril, Longes) | Angioedema may occur. In the event these drugs are being administered, they should be discontinued at least 36 hours prior to initiating administration of this drug. In the event these drugs are administered following administration of this drug, do not administer them until 36 hours after the final administration of the drug. | Concomitant use may additively inhibit bradykinin degradation and increase the risk of angioedema. |
| Aliskiren fumarate (Rasilez)  (When administered to diabetic patients) | Increased risks of nonfatal stroke, renal dysfunction, hyperkalemia, and hypotension have been reported with valsartan. | Concomitant use may enhance the inhibitory effect on the renin-angiotensin-aldosterone system. |

2. Precautions on concomitant use (be careful when using in combination)

| Drug name etc. | Clinical symptoms/treating methods | Mechanism/risk factors |
| --- | --- | --- |
| Angiotensin II receptor antagonist | It should not be used concomitantly with these drugs, as it may cause renal dysfunction, hyperkalemia, and hypotension. | Concomitant use may enhance the inhibitory effect on the renin-angiotensin-aldosterone system. |
| Aliskiren fumarate | It may cause renal dysfunction, hyperkalemia, and hypotension. Concomitant use with aliskiren fumarate in patients suffering from renal impairment with an eGFR of less than 60 mL/min/1.73 m^2^ should be avoided, unless it is considered unavoidable for therapeutic reasons. |  |
| Atorvastatin | Concomitant use may increase the blood concentration of atorvastatin. | This drug may inhibit the uptake of drugs into the liver via OATP1B1 and OATP1B3. |
| PDE5 inhibitor  Sildenafil, etc. | It has been reported that in hypertensive patients, the combination of this drug and sildenafil lowered blood pressure more than the administration of this drug alone. Use caution when initiating sildenafil or other PDE5 inhibitors in patients receiving this drug. | PDE5 inhibitors inhibit cGMP degradation, which increases with administration of this drug. |
| PDE5 inhibitors inhibit cGMP degradation, which increases with administration of this drug. | There is a risk that serum potassium and serum creatinine levels may increase. | The suppression of aldosterone secretion by this drug may enhance the potassium retention effect. Risk factor: Renal dysfunction |
| Drospirenone/Ethinylestradiol | Serum potassium levels may increase. | This is thought to be due to the increase in serum potassium level caused by valsartan and the antimineralocorticoid effects of drospirenone. Risk factors: patients with renal impairment, patients with high serum potassium levels |
| Pharmaceutical preparation containing Trimethoprim, Sulfamethoxazole/Trimethoprim |  | There is a risk that the increase in serum potassium level may be bolstered. |
| Ciclosporin |  | It is thought that the side effects of hyperkalemia are mutually bolstered. |
| Diuretic antihypertensive agents, such as furosemide trichlormethiazide | There is a risk of a sudden drop in blood pressure (accompanied by syncope and loss of consciousness). Additionally, the diuretic effect may be bolstered. | Plasma renin activity increases during diuretic antihypertensive drug administration, so concomitant use with these drugs may bolster the renin-angiotensin-aldosterone system inhibitory effect. Rarely, symptomatic hypotension may occur in patients with severe sodium or body fluid volume depletion. |
| Nonsteroidal anti-inflammatory drugs such as (NSAIDs) indomethacin | There is a risk of worsening renal function. | This is thought to be due to a decrease in renal blood flow, caused by the glandin synthesis inhibitory effect. Risk factors: elderly individuals, body fluid volume-depleted patients (including those using diuretics), and patients with impaired renal function |
| Lithium | Renin-angiotensin-aldosterone system inhibitors have been reported to cause lithium toxicity. The risk of lithium toxicity may further increase in the event diuretics are used. | It is thought that lithium accumulation occurs due to the sodium excretion effect of this drug. |
| Ciclosporin  Clarithromycin  Erythromycin | There is a risk that exposure to Sacubitrilat or Valsartan may increase and side effects may be exacerbated. | Inhibiting OATP1B1 or OATP1B3 may increase the blood concentrations of sacubitrilat and valsartan. |
| Bixalomer | It has been reported that the blood concentration of valsartan decreased by approximately 30-40%. There is a risk that the effect of this drug may be weakened. | Phosphate-binding polymers can delay or reduce the absorption of valsartan when taken together. |

4.6.5 Side effects

Because the following side effects may occur, carefully monitor the patient, and if any abnormalities are observed, take appropriate measures such as discontinuing administration.

In a domestic phase III study (B1301 study, PARALLEL-HF) and an overseas phase III study (B2314 study, PARADIGM-HF) targeting patients with chronic HF, 966 (22.4%) out of 4,314 cases that were investigated revealed the occurrence of side effects, etc. The main side effects included blood and lymphatic system disorders in 7 cases (0.2%), heart disorders in 70 cases (1.6%), ear and labyrinth disorders in 10 cases (0.2%), and gastrointestinal disorders in 40 cases (0.9%) (From Pharmaceutical Interview Home).

4.6.6 Serious side effects

1) Angioedema (0.2%)

Angioedema, which can lead to airway obstruction, may appear as symptoms such as swelling of the tongue, glottis, and larynx. In such cases, administration should be immediately discontinued and appropriate measures such as epinephrine injection and airway maintenance should be taken. Do not re-administer even if angioedema has resolved.

2) Renal dysfunction (2.9%), renal failure (0.8%)

3) Hypotension (10.4%)

4) Hyperkalemia (4.7%)

5) When shock (< 0.1%), syncope (0.2%), loss of consciousness (< 0.1%), cold sensation, vomiting, loss of consciousness, etc. occur, take appropriate measures immediately.

6) Agranulocytosis, leukopenia, thrombocytopenia (incidence unknown)

7) Interstitial pneumonia (< 0.1%)

Interstitial pneumonia accompanied by fever, cough, difficulty breathing, abnormal chest X-rays, etc. may occur. In such cases, administration should be discontinued and appropriate measures such as administration of adrenocortical hormones should be taken.

8) Hypoglycemia (incidence unknown)

If any symptoms such as weakness, hunger, cold sweats, hand tremors, decreased concentration, convulsions, or impaired consciousness occur, administration should be discontinued and appropriate measures should be taken. It is more likely to occur in patients undergoing diabetes treatment.

9) Rhabdomyolysis (incidence unknown)

Rhabdomyolysis, which is characterized by muscle pain, weakness, increased CK, and increased myoglobin in blood and urine, may occur. In such cases, administration should be immediately discontinued and appropriate measures should be taken.

10) Toxic epidermal necrolysis (TEN),

mucocutaneous ocular syndrome (Stevens-Johnson syndrome), and erythema multiforme (incidence unknown for all)

11) In the event pemphigus, pemphigoid (incidence unknown), blisters, erosion, etc. occur, consult a dermatologist.

12) Hepatitis (incidence unknown)

4.6.7 Other side effects

|  | ≥ 0.3% | < 0.3% | Incidence unknown |
| --- | --- | --- | --- |
| Infectious diseases and  parasitic disease | ― | ― | Pharyngitis |
| Blood and  lymphatic system disorder | ― | Anemia | Eosinophilia |
| Metabolism and  malnutrition | ― | Hypokalemia, decreased appetite, hyponatremia | ― |
| Nervous system disorders | Dizziness | Postural dizziness, vertigo, headache, insomnia, taste abnormality, drowsiness, numbness | ― |
| Ear and labyrinth disorders | ― | ― | Tinnitus |
| Heart disorders | ― | Palpitations, atrial fibrillation | Tachycardia |
| Vascular disorders | Orthostatic hypotension | ― | Hot flashes |
| Respiratory, thoracic and  mediastinal disorders | Cough | ― | ― |
| Gastrointestinal disorders | ― | Diarrhea, nausea, abdominal pain, constipation | Vomiting |
| Skin and  subcutaneous tissue disorders | ― | Hives | Erythema, photosensitivity |
| Musculoskeletal system and  connective tissue disorders | ― | Joint pain, lower back pain | Muscle pain |
| General/systemic disorders and administration site conditions | ― | Fatigue, asthenia,  malaise | Dry mouth, edema,  chest pain, fever |
| Immune system disorders | ― | Hypersensitivity (including rash, pruritus, and anaphylactic reactions) | ― |
| Clinical examination | ― | Increased AST, increased ALT, increased blood uric acid level, increased BUN, increased serum creatinine, increased serum potassium level, increased blood sugar level | Increased bilirubin level, increased LDH, increased CK, increased serum cholesterol, decreased serum total protein, increased ALP |

## Regulation of concomitant drugs and concomitant therapies

### Sacubitril/valsartan group

Concomitant use with ACE inhibitors (because angioedema can develop), ARBs (because they can cause renal dysfunction, hyperkalemia, and hypotension), and aliskiren fumarate (for non-fatal stroke [in diabetic patients] because it may cause renal dysfunction, hyperkalemia, hypotension, etc.) are prohibited.

### Control group

Use of sacubitril/valsartan is prohibited (because it is expected to have an effect on study endpoints).

Although new administration of SGLT2 inhibitors and MRA will not be conducted, in principle, in both groups, there will be no restrictions on the use of other drugs (HF drugs including diuretics and drugs for other comorbidities) and guidance on therapy. However, during the protocol treatment period, in principle, the usage and dosage of these drugs will not be changed; however, changes are allowed at the discretion of the doctor in charge, depending on the medical state of the study participant.

# **Efficacy and safety endpoint**

## Efficacy endpoints

Primary endpoint

Between-group ratio of the proportional change in the geometric mean NT-proBNP at 8 weeks following protocol treatment initiation, compared with baseline

Secondary endpoints

1. Between-group ratio of the proportional change in geometric mean NT-proBNP at 4 weeks following protocol treatment initiation, compared with baseline.
2. Percentage of cases in which the NT-proBNP value decreased by 50% or more from the baseline, at 8 weeks following protocol treatment initiation.
3. Percentage of patients with NT-proBNP levels reduced by 30% or more from baseline at 4 weeks following protocol treatment initiation, Cardiac troponin.
4. Percentage of cases which mean NT-proBNP value decreased by 40% or more from the baseline at 4 and 8 weeks following protocol treatment initiation.
5. Change from the baseline of cardiac troponin T, CRP, GDF-15, soluble ST2, glycoalbumin, and 1.5AG at 8 weeks after following protocol treatment initiation.
6. Change from the baseline of weight, BMI, blood pressure, pulse rate, laboratory test values, and NYHA class at the 4 and 8 weeks following protocol treatment initiation.
7. Change from the baseline of cardiac function indicators (LVEDV, LVESV, LVEF, septal e', lateral e', mitral orifice blood flow velocity waveform (E), E/e', LVMI, LAVI, LVOT, LVOT-VTI, TR velocity, IVC, GLS, left atrial strain (2-chamber view and 4-chamber view)), and percentage of cases with a respiratory fluctuation of 50% or more in IVC.
8. Change from the baseline of KCCQ-12 at 8 weeks following protocol treatment initiation, along with the percentage of cases whose score increased by 5 points or more.
9. Time to composite outcomes of first HF event [the following events due to the exacerbation of HF: i) unplanned rehospitalization ii) initiation of intravenous treatment for HF (vasodilators, inotropes) (during hospitalization: excluding rehospitalization); iii) emergency visit for HF requiring intravenous therapy (vasodilators, inotropes, diuretics); and iv) initiation of oral diuretics (loop diuretics, thiazide diuretics, tolvaptan) or a dose ;increase of ≥50% (outpatient)] and all-cause mortality.
10. Number and frequency of composite outcomes of HF events including recurrence and all-cause mortality.
11. Number and frequency of following individual events: first or recurrent HF events, all-cause mortality, and cardiovascular death*.
12. Number of occurrences and time to onset of specific adverse events, such as worsening of renal function (more than 50% increase in serum Cr or more than 30% decrease in eGFR), hyperkalemia (serum potassium 5.5 mEq/L or more), symptomatic hypotension, and angioedema.
13. Number of occurrences of other serious adverse events

*Cardiovascular death is defined as a case in which the main cause of death is determined to be atherosclerotic cardiovascular disease, HF, or sudden cardiac death (including cases in which death is not clearly caused by non-cardiovascular disease).

## Safety endpoint

Adverse events that occurred following the protocol treatment initiation

### Evaluation of adverse events

The severity, seriousness, predictability, causal relationship with the implementation of the study, and causal relationship with the study drug shall be evaluated as follows.

### Severity

The severity of adverse events will be graded as mild, moderate, or severe. The criteria for each shall be as follows:

- 1. Mild: Does not interfere with daily activities
  2. Moderate: Interferes with daily activities
  3. Severe: Unable to perform daily activities

### Seriousness

Seriousness is determined as either serious or non-serious. Being serious means it applies to any of the following from 1) to 7):

- 1. Death
  2. Diseases that may lead to death
  3. Diseases, etc. that require hospitalization at a medical institution, or an extension of the hospitalization period for treatment
  4. Disability
  5. Diseases that may lead to disability
  6. 3) to 5), and diseases that are serious in accordance with death and diseases that may lead to death
  7. Congenital diseases or abnormalities in later generations

### Causal relationship with implementation of the study

Determine the causal relationship between the adverse event and the implementation of the study treatment, as either related or unrelated. "Related" means that the relationship between the adverse event and implementation of the study cannot be ruled out.

### Causal relationship with the study drug (sacubitril/valsartan)

The causal relationship of the adverse event to sacubitril/valsartan will be determined as either related or unrelated.

"Related" means that the relationship between the adverse event and the administration of sacubitril/valsartan cannot be ruled out. If it cannot be determined as related, it is determined as not-related.

### Predictability

Predictability is determined as either a predictable adverse event (known) or an unpredictable adverse event (unknown). An adverse event that is not described in the following documents and cannot be predicted is considered an unpredictable adverse event.

Latest attached documents for sacubitril/valsartan

The side effect information listed in the second edition of the attached documents for sacubitril/valsartan (revised in August 2020) is as described in "4.6.5 Side effects."

### Outcome and outcome date

The outcome of adverse events will be determined as recovery, remission, non-recovery, sequelae, death, or unknown.

The outcome date shall be the date on which the representative investigator or sub-investigator confirms the outcome. If the outcome is not remission, recovery, sequelae, or death and is not resolved or unknown, follow-ups will continue until the end of the study participant's observation period, after which the outcome and outcome date will be entered in the EDC.

## Handling when an adverse event occurs

### Definition of adverse events, diseases, etc. and side effects

An adverse event is any unwanted or unintended sign (including laboratory abnormalities), symptom, or illness occurring to a study participant, whether or not causally related to the study.

Diseases, etc. include diseases, disabilities, death, or infectious diseases that are suspected to be caused by the study implementation, as well as abnormal laboratory test values and various symptoms.

Side effects are defined as adverse events for which the causal relationship with sacubitril/valsartan is determined as "related.”

In this study, we will collect all adverse events that occur to study participants from the date of obtaining consent until the end of the observation period.

## Measures to be taken if an adverse event occurs

### Measures taken for the study participants

If an adverse event occurs, the representative investigator or sub-investigator will take appropriate measures to ensure the safety of the study participants, such as discontinuing treatment and the administration of sacubitril/valsartan, as necessary. If treatment, etc. is required, the study participants shall be notified accordingly.


### Reporting of adverse events

If an adverse event becomes known, the representative investigator or sub-investigator will promptly assess the severity, causal relationship with the implementation of the study, causal relationship with sacubitril/valsartan, predictability, and outcome. If it falls under the category of illness, etc., the representative investigator will report it in accordance with "5.4.3 Reporting of Illness, etc." The principal investigator will report any serious adverse events related to sacubitril/valsartan to Novartis Pharma Co., Ltd. within 15 days after the sub-investigator becomes aware thereof. Additionally, the principal investigator will report all adverse events after the baseline to Novartis Pharma Co., Ltd., regularly and following the end of the study period.

The representative investigator or sub-investigator will enter the following information regarding the adverse event into the EDC:

- 1. Adverse event name
  2. Date of occurrence
  3. Severity
  4. Seriousness (severe/non-severe)
  5. Causal relationship with the implementation of this study
  6. Causal relationship with sacubitril/valsartan
  7. Predictability (known/unknown)
  8. Outcome and outcome date

### Reporting of illness etc.

Because this study falls under the category of specific clinical research, aside from specific clinical research using unapproved or off-label drugs, the principal investigator must report the illness, etc. upon its occurrence to the CRB in accordance with the provisions of Ministry of Health, Labor and Welfare Ordinance No. 17 of 2018).

Should the principal investigator learn of the occurrence of a serious disease, the representative investigator will immediately report to the administrator of the medical institution and the principal investigator, who will report to the CRB. This does not mean that report must be made to the administrator of the medical institution before reporting to the CRB, with the order of reporting capable of being changed depending on the situation.

If the principal investigator becomes aware of the occurrence of a disease other than a serious disease, the principal investigator will report to the administrator of the medical institution conducting the study once a year, and periodically report to the CRB. In addition, the representative investigator will comply with the reporting requirements of each medical institution.

Regarding serious adverse events that do not fall under the above diseases, the representative investigator will promptly convey information to the principal investigator, in accordance with the procedure manual of the institution to which the representative investigator belongs, and when the representative investigator receives information on serious adverse events that do not fall under the above categories, he/she will promptly report them to the Hospital Director and the Saga University Clinical Research Review Committee.

Details of the procedures and reporting deadlines are separately stipulated in the "Procedure Manual for Emergency Reporting of Diseases, etc."

# **Compliance with the study plan and management of nonconformities**


## Compliance with the study plan

The representative investigator and sub-investigator will conduct this study in compliance with the study plan.

Nonconformity refers to a state in which this study does not comply with the Clinical Research Act and Ministerial Ordinance or the study plan (deviation). Specifically, it refers to non-compliance with enforcement regulations, implementation plans, study plans, procedure manuals, etc., and falsification or fabrication of research data. A major nonconformity is a case that affects the human rights and safety of the study participants, the progress of study results, and the reliability of results; for example, non-compliance with inclusion/exclusion criteria, discontinuation criteria, prohibited concomitant therapies, etc. This does not include cases in which the study plan was not followed for medically unavoidable reasons, such as to avoid immediate danger to the study participants.

The principal investigator and sub-investigator will not make any deviations without the prior approval of the CRB. However, if it is medically unavoidable, such as to avoid immediate danger to the study participants, the principal investigator and sub-investigators may deviate from the study plan without prior approval from the CRB.

## Reporting and recording of nonconformities

When the sub-investigator learns that the clinical research is inappropriate, he/she shall promptly report it to the representative investigator. When the principal investigator becomes aware of non-compliance, he/she will promptly report this to the administrator of the medical institution and notify the principal investigator thereof. In addition, if a particularly serious nonconformity is discovered, the principal investigator will promptly seek the opinion of the CRB. Furthermore, when the principal investigator becomes aware of non-compliance, he/she will promptly provide information to that effect to other representative investigators.

The representative investigator and sub-investigators will record all actions deviating from the research protocol, regardless of the reason.

## Obtaining permission for implementation at the medical institution

The principal investigator will submit the implementation plan to the Minister of Health, Labor and Welfare, after hearing the opinion of the CRB and upon obtaining approval from the administrator of each medical institution.

# **Statistical analysis**

## Target number of cases

Overall target number of cases: 400 cases (sacubitril/valsartan administration group: 200 cases, control group: 200 cases)

[Basis for setting]

Regarding the basis for setting the target number of cases in this study, we will first describe examples from previous studies, then describe the process for setting the target number of cases in this study.

1. Examples of previous study
   1. In the PIONEER-HF study conducted in the United States, which examined the effects of sacubitril/valsartan on NT-proBNP in HFrEF patients hospitalized with acute HF, the reduction rate of the mean geometric mean NT-proBNP at 4 and 8 weeks following the initiation of administration was 46.7% in the sacubitril/valsartan group (ratio of geometric mean value to the baseline value: 0.53) and 25.3% in the control group (enalapril) (said ratio: 0.75), with an absolute difference between the groups of 21.4%, while the ratio of change between treatment groups (sacubitril/valsartan vs. enalapril) was 0.71 (95% CI 0.63 to 0.81, P<0.001).
   2. Similarly, the TRANSITION study of HFrEF patients hospitalized with acute HF, indicated that initiating sacubitril/valsartan during hospitalization reduced NT-proBNP by 25.1% at 4 weeks and 38.0% at 10 weeks.
   3. The PARAMOUNT study in HFpEF patients (chronic HF) indicated that the rate of change in NT-proBNP from the baseline to 12 weeks following administration was 22.7% in the sacubitril/valsartan group and 3.1% in the control group (valsartan), with an absolute difference between the groups of 19.4%, while the ratio of percent change between treatment groups (sacubitril/valsartan vs. valsartan) was 0.77 (95% CI 0.64 to 0.9, P=0.005).
   4. Furthermore, the PARALLEL-HF targeting Japanese HFrEF patients (chronic HF) indicated that the rate of decrease in NT-proBNP from the baseline to 4 weeks following the initiation of treatment was 23.3% in the valsartan group and 11.4% in the control group (enalapril), with an absolute difference of 11.9%, while the ratio of change between treatment groups (sacubitril/valsartan vs. enalapril) of 0.87 (P=0.033). The decrease rate of NT-proBNP from the baseline to 8 weeks following the initiation of treatment was 22.0% in the sacubitril/valsartan group and 9.7% in the control group, with an absolute difference of 12.3% between the groups, while the ratio of change rate (same) was 0.85 (P=0.016).

Based on the results of the above previous studies, sacubitril/valsartan for HF patients (regardless of HFrEF or HFpEF) has a significant effect on reducing NT-proBNP compared to the control group (standard HF treatment). It is inferred that the effect is equivalent or higher even when targeting acute HF patients compared to chronic HF patients. On the other hand, as seen in the PARALLEL-HF trial conducted in Japan, it is possible that the therapeutic effect for Japanese patients may be slightly smaller compared to the results of studies conducted in the United States. However, the dose of enalapril used in the control group of the same study was higher than the approved dose in Japan, leading to a potential underestimation of the difference in treatment effect between the groups compared to the previous standard treatment.

Judging from the above, in this study, we assumed that the absolute difference in the geometric mean NT-proBNP reduction rate between the treatment groups would be 19% and so set the required minimum number of cases at 400 cases (200 cases in each group, to detect a significant difference between the two groups, under the conditions in which the standard deviation for both groups was 0.70 (logarithmic scale), the significance level was 5% on both sides, and the detection power of 80% with a 13% dropout rate.

## Analysis set


### Efficacy analysis set

### Full analysis set (FAS)

In accordance with the intention-to-treat principle, the full analysis set (FAS) excluding the following cases, will be the efficacy set:

- - Cases in which consent was withdrawn after registration
  - Cases found to be ineligible after registration
  - Cases that have not received any protocol treatment after allocation
  - Cases without data regarding efficacy after protocol treatment initiation

### Per-protocol set (PPS)

Among FAS, the patient population without major protocol deviations will be the per-protocol set (PPS) and will be the set for supplementary efficacy analysis.

The data center will evaluate protocol deviations as the number of cases accumulates, hold case review meetings between the principal research physician, the person in charge of statistical analysis, and the data center before locking in the research data, and establish how to handle any major protocol deviations and cases.

### Safety set (SS)

Randomly allocated patients who have received at least one protocol treatment will be considered as the safety set (SS).

## Analysis method

Regarding efficacy, FAS will be the main analysis set, with a supplementary analysis targeting PPS also being conducted for sensitivity analysis. The significance level for all statistical tests is two-sided 0.05. Regarding the details of analysis not described in this study plan and abnormal data including outliers, we will consider how to handle them before conducting the statistical analysis and create and define a statistical analysis plan before data is fixed. If the statistical analysis plan is changed due to a change in the study plan, the study plan and statistical analysis plan will be revised and the details of the revision will be explained in the study summary report.


### Case composition

A flow chart will be created for cases in which consent was obtained, cases not registered, cases registered, cases with protocol treatment initiation, cases discontinued, cases completed, and cases in which primary endpoint analysis was possible. Cases of discontinuation prior to the protocol treatment initiation and cases of discontinuation during the course of treatment will be tabulated by reason and a list will be created.

### Analysis target description

The number of cases is indicated for each FAS, PPS, and SS. Excluded cases will be tabulated by reason for exclusion and a list of excluded cases will be created.

### Patient characteristics and baseline values

Regarding FAS patient demographics, quantitative variables were summarized statistics (number of cases, mean value, standard deviation, minimum value, 1st quartile [Q1:25 percentile], median value, 3rd quartile [Q3: 75 percentile], maximum value), and the number of cases and percentage (%) will be described for qualitative variables for each treatment group. In addition, data such as the number of cases and percentage (%) of complications and use of prior treatment drugs will be described for each treatment group.

## Analysis of efficacy


### Analysis of efficacy

1. Analysis of primary endpoint

Regarding the proportional change in geometric mean NT-proBNP from the baseline to 8 weeks following the protocol treatment initiation, an analysis will be performed using a linear mixed model (MMRM) with the allocation as fixed effects, the baseline value, evaluation time and interaction between allocation and time as covariates, and the patient as a random effect, along with the treatment effect estimated by the ratio of the geometric mean based on the least squares mean at 8 weeks, the two-sided 95% confidence interval, and the between-group ratio will be calculated.

1. Analysis of secondary endpoints
2. Between-group ratio of proportional change in geometric mean NT-proBNP at 4 weeks following the protocol treatment initiation compared with baseline (key secondary endpoint):

Regarding the proportional change in geometric mean NT-proBNP from the baseline to 4 weeks following the protocol treatment initiation, an analysis will be performed using a linear mixed model (MMRM) with the allocation as fixed effects, the baseline value, evaluation time and interaction between allocation and time as covariates, and the patient as a random effect, along with the treatment effect estimated by the ratio of the geometric mean based on the least squares mean at 4 weeks, the two-sided 95% confidence interval, and the between-group ratio will be calculated.

1. Percentage of cases whose NT-proBNP value decreased by 50%/30%/40% or more from the baseline, at 8 weeks/4 weeks/average of 4 and 8 weeks, respectively, after protocol treatment initiation:

Regarding the percentage of patients who achieved the defined rate of decline in NT-proBNP at each point, a logistic regression analysis adjusted for the baseline NT-proBNP values was used to calculate the adjusted odds ratio (sacubitril/valsartan group vs. control group) and a two-sided 95% confidence interval.

1. Change from the baseline of blood biomarkers at 4 and 8 weeks following protocol treatment initiation (cardiac troponin T, CRP, GDF-15, soluble ST2, glycoalbumin, 1.5AG) and all other laboratory test values (8 weeks only for blood biomarkers and cardiac ultrasound):

Calculate summary statistics (number of cases, mean value, standard deviation, minimum value, median value, maximum value, first quartile, third quartile) of the measured value and change at each time point. Regarding the change in each outcome (8 weeks only for blood biomarkers and cardiac function tests) from the baseline at 4 and 8 weeks following the protocol treatment initiation, an analysis will be performed using MMRM with the allocation as fixed effects, the baseline value, evaluation time and interaction between allocation and time as covariates, and the patient as a random effect, along with the treatment effect estimated by the change or proportional change based on the least squares mean at 4 and 8 weeks, and the two-sided 95% confidence interval will be calculated. If the distribution of measured values deviates significantly from a normal distribution, logarithmic transformation is performed. No redundancy adjustment will be performed.

1. Change from the baseline in KCCQ-12 at 8 weeks following protocol treatment initiation and percentage of cases whose score increased by 5 points or more:

Calculate the summary statistics (number of cases, mean value, standard deviation, minimum value, median value, maximum value, first quartile, third quartile) of the measured value and amount of change at each time point. Regarding the changes in the KCCQ-12 overall summary score from the baseline to 8 weeks following protocol treatment initiation, an analysis will be performed using MMRM with the allocation as fixed effects, the baseline value, evaluation time and interaction between allocation and time as covariates, and the patient as a random effect, along with the treatment effect estimated by the change based on the least squares mean at 8 weeks, and the two-sided 95% confidence interval will be calculated. Regarding the percentage of patients achieving an increase of 5 points or more in the KCCQ-12 overall summary score at 8 weeks, a logistic regression analysis adjusted for the baseline KCCQ-12 overall summary score was used in order to determine the adjusted odds ratio (sacubitril/valsartan group vs. control group) and the two-sided 95% confidence interval.

1. Number, frequency and time to composite outcomes of first HF event [the following events due to exacerbation of HF: i) unplanned rehospitalization; ii) initiation of intravenous treatment for HF (vasodilators, inotropes) (during hospitalization: excluding rehospitalization); iii) emergency visit for HF requiring intravenous therapy (vasodilators, inotropes, diuretics); and iv) initiation of oral diuretics (loop diuretics, thiazide diuretics, tolvaptan) or dose increase of 50% or more (outpatient)], and all-cause mortality and each of these individual events:

Using the treatment group as a fixed effect factor, we will describe the survival curve using the Kaplan-Meier method, then calculate the hazard ratio (sacubitril/valsartan group vs. control group) and two-sided 95% confidence interval, using the Cox proportional hazards model. When evaluating individual events, the occurrence of cardiovascular death should also be treated in the same manner.

1. Number and frequency of composite outcomes of HF events including recurrence [the following events due to exacerbation of HF: i) unplanned rehospitalization; ii) initiation of intravenous treatment for HF (vasodilators, inotropes) (during hospitalization: excluding rehospitalization); iii) emergency visit for HF requiring intravenous therapy (vasodilators, inotropes, diuretics); and iv) initiation of oral diuretics (loop diuretics, thiazide diuretics, tolvaptan) or dose increase of 50% or more (outpatient)], and all-cause death, and each of these individual events:

Using a numerical data analysis method that takes into account competing risks, with the treatment group as a fixed effect factor, calculate the hazard ratio of event occurrence (sacubitril/valsartan group vs. control group) and two-sided 95% confidence interval, including recurrence. When evaluating individual events, the occurrence of cardiovascular death should also be treated in the same manner.

Regarding the primary and secondary endpoints related to NT-proBNP and events such as worsening HF and all-cause mortality, we will examine the presence or absence of interactions between pre-specified variables and perform a subgroup analyses. The details of the variables to be specified in advance will be determined before the data is fixed and the details will be recorded in the statistical analysis plan.

### Safety analysis

Reported adverse events will be assigned a Low Level Language (LLT) code using the MedDRA/J dictionary. The version of MedDRA/J used for analysis shall be the latest version at the time the database is locked in.

Adverse events (TEAEs) that occurred since protocol treatment initiation will be tabulated. Adverse events for which a causal relationship with sacubitril/valsartan is determined as "related" are considered side effects.

If an adverse event with the same preferred term (PT) occurs multiple times in one example, the number of occurrences is counted as the number of cases, while the number of examples is counted separately as one case.

For all adverse events and side effects, calculate the number of occurrences, number of cases, incidence rate, and the 95% confidence interval for each treatment group.

The number of occurrences, the number of cases that occurred, and the incidence rate will be calculated for each treatment group according to the System Organ Class (SOC) and PT classification.

# **Interim analysis**

This will not be implemented.

# **Viewing of original source materials, etc.**

In this study, the representative investigator and the medical institution conducting the study shall make all related records, including original source materials, available for direct viewing during monitoring related to this study, as well as for investigations by the CRB and regulatory authorities.

# **Quality control and quality assurance**

## Monitoring methods

In this study, monitoring will be conducted in accordance with a separately prepared "Procedure Manual for Monitoring." Persons engaged in monitoring (monitors) shall confirm the following items throughout the study period, in accordance with the monitoring procedure manual. Monitors must not leak the information obtained in the course of their work.

1) The human rights and safety of the study participants are protected and ensured.

2) The study has been conducted in compliance with the latest study plan, implementation plan, and regulatory requirements.

3) The accuracy of the CRF can be verified against original source materials, etc.

# **Matters related to ethical considerations**

This study is conducted in accordance with ethical principles based on the Declaration of Helsinki, the Clinical Research Act (Act No. 16 of 2017), the Enforcement Regulations of the Act (Ministry of Health, Labor and Welfare Ordinance No. 17 of February 28, 2018), and in compliance with notices related to these matters.

## Benefits, burdens, and anticipated disadvantages to study participants

The results of this study confirm the safety and effectiveness of sacubitril/valsartan for patients hospitalized due to worsening HF, so if it is confirmed that it may be a useful treatment, it may contribute to the advancement of medical care in the future.

Because this study will be carried out, in principle, within the scope of normal insurance medical treatment, the health insurance of the study participants will cover observations, tests, medications, etc. during the study period. However, some tests (blood biomarkers) conducted at baseline and at 8 weeks are not covered by health insurance; therefore, the test costs will be covered by research funds. Additionally, participating in this study requires additional blood collection for blood biomarker measurement, in addition to blood collection performed during regular medical treatment, which will require approximately 9 mL at baseline, once every 8 weeks, and approximately 1.5 mL at 4 weeks (a total of approximately 19.5 mL).

Side effects that may occur when participating in this study and taking sacubitril/valsartan are listed in "4.6.5 Side effects."

The representative investigator or sub-investigator shall promptly conduct appropriate examinations and treatment in the event of an adverse event (including side effects). In order to minimize the disadvantages caused by adverse events, we will collect the information necessary to safely conduct this study and take appropriate measures such as changing the study plan as necessary.

The observation period was set at 8 weeks from the viewpoint of patient protection, in order to avoid restricting patient treatment and eliminating the possibility of long-term administration of sacubitril/valsartan in the control group.

## Handling of personal information

The representative investigator and sub-investigator will give due consideration to the protection of each study participant's personal information when creating and handling the CRF. Each study participant will be identified by a patient identification number. The representative investigator and sub-investigator will create an anonymization table in order to identify individual study participants, store and manage it appropriately within the medical institution conducting the study, and not provide it to anyone outside the medical institution conducting the study.

When publishing the results of this study, sufficient care will be taken to handle personal information so that individual study participants cannot be identified.

If a study participant requests disclosure of his/her personal information that can be identified as him/her among the stored personal information, the representative investigator shall promptly provide the relevant personal information to the person who made the request. The study plan and content of the study will also be disclosed.

# **Matters regarding handling and storage of records (including data)**

The principal investigator must appropriately preserve the following information and related records concerning this study until the day on which at least 5 years have elapsed from the completion of the study. The samples collected and measured at each medical institution will be appropriately disposed of at each medical institution.

1. Materials that identify study participants (anonymization table)
2. Medical and laboratory records of study participants
3. Study participant consent form (signed original)
4. Paper forms or data regarding central measurement results sent to the principal investigator from SRL Co., Ltd. and the Department of Cardiovascular Medicine, Tokushima University Hospital
5. Study plan, explanatory document, and consent form for this study
6. Documents submitted to the CRB, such as implementation plans
7. CRB result notification
8. Documents concerning monitoring
9. Contracts concluded regarding the implementation of this study (excluding contracts concluded with funders)
10. Other documents and records related to this study

Data used in the study will be provided to the data center from medical institutions conducting the study through EDC. The research office, monitoring staff, and statistical analysis staff will be permitted to access the collected data stored at the data center, based on specific access authorization such as IDs and passwords. The data center will appropriately store and manage the provided information until the data has been delivered to the representative investigator.

Once the storage period ends, paper media will be shredded and destroyed. Other information will be anonymized and disposed of in an appropriate manner.

# **Matters regarding the payment of money and compensation**

## Regarding financial burden associated with the study

The costs of the research treatment and tests and surveys conducted in this study will be borne by the study participants, just like regular medical care. Because this study will be carried out, in principle, within the scope of normal insurance medical treatment, the health insurance of the study participants will cover observations, tests, medications, etc. during the study period. However, some tests (blood biomarkers) conducted at baseline, at 4 weeks, 8 weeks, and at the time of discontinuation are not covered by health insurance, therefore, the test costs will be covered by research funds. Study participants will be provided with a burden reduction fee of 5,000 yen for the baseline, 5,000 yen for 8 weeks, and a total of 10,000 yen in the form of a QUO card, etc.

## Compensation and indemnity for health damage caused by the study

In the event that health damage occurs as a result of participating in this study, the representative investigator and sub-investigator will provide appropriate treatment and measures to the study participant. Treatment, etc. in this case will be provided as covered by insurance, with study participants paying their own medical expenses. Furthermore, because the treatment in this study is subject to the Relief System for Sufferers from Adverse Drug Reactions, the system may apply if health damage occurs. However, in preparation for liability for serious health damages (class 1 disability, class 2 disability, death), the principal investigator will enroll in liability insurance under Clinical Research Insurance. Other treatments will be covered by the study participants' health insurance.

## Clinical research insurance coverage


1. Compensation (death/residual disability compensation money)

(1) If the study participant is a patient (per study participant)

(The grade of residual disability shall be in accordance with the grade specified by the Japan Pharmaceutical Adverse Reaction Relief and Research Promotion Agency.)

| Insurance claim classification | Payment category | 1 study participant  Compensation limit | | 1 accident/1 clinical research compensation limit |
| --- | --- | --- | --- | --- |
| Death compensation insurance money | If the study participant is a provider of living expenses | ¥20M | | ¥100M |
|  | If the study participant is not a provider of living expenses | ¥7M | |  |
| Residual disability compensation insurance money | If the study participant is a provider of living expenses | Residual disability level 1 | ¥30M |  |
|  |  | Residual disability level 2 | ¥24M |  |
|  | If the study participant is not a provider of living expenses | Residual disability level 1 | ¥20M |  |
|  |  | Residual disability level 2 | ¥16M |  |

# **Matters regarding publication of information concerning the study**

## Registration of study plan

The principal investigator will register the outline of the study on jRCT and ClinicalTrials.gov prior to implementation and will update it as appropriate in response to changes in the implementation plan and progress of the study. When the study is completed, the results of the study will be registered without delay.

## Publication of results

The results will be published after taking necessary measures to protect the human rights of research participants and their associates, as well as the rights and interests of those involved in this study and their associates.

The principal investigator will prepare a main evaluation item report, a comprehensive report, and a summary thereof within the following period. Once created, the principal investigator will listen to the opinions of the CRB written in the implementation plan, then promptly submit it to the administrator of the implementing medical institution and register the summary in jRCT. If the main evaluation item report and the comprehensive report are created at the same time, it will be assumed that the main evaluation item report has also been created, due to the creation of the comprehensive report.

1) Main evaluation item report

In principle, within one year after the end of the period for collecting data related to the evaluation items

2) Comprehensive report and a summary thereof

In principle, within one year after the end of the period for collecting data related to all evaluation items

# **Study implementation period**

## Study implementation period

Case registration period: Clinical study implementation plan/study summary publication system (jRCT) release date to:

Friday, March 31, 2023

Observation period: 8 weeks

Study implementation period: Clinical study implementation plan/study summary publication system (jRCT) release date to:

Monday, March 31, 2025

## Completion of the study

This study will be completed when the results are published on jRCT, a database maintained by the Ministry of Health, Labor and Welfare. The principal investigator will provide the information on the contents of the published information to the representative investigator at each implementing medical institution. The representative investigator will report the information provided by the principal investigator to the administrator of the implementing medical institution.

# **Explanation and consent for study participants**

## Procedures for obtaining informed consent

- 1. Prior to a patient's participation in the study, the representative investigator or sub-investigator shall hand deliver the patient an explanatory document and consent form that have been approved by the CRB and verbally sufficiently explain the contents listed in "16.2 Explanation to Study Participants.” Upon giving the patient sufficient time to decide whether or not to participate in this study and confirming that the patient fully understands the content, obtain the patient's voluntary written consent to participate in the study.
  2. The representative investigator or sub-investigator who provided the explanation to the patient, should affix his/her name and a seal or signature in the consent form, and record the date. If a research collaborator provides supplementary explanations, the research collaborator should also affix his/her name and a seal or signature, and record the date.
  3. The original consent form with name and seal or signature and date will be stored by the representative investigator or sub-investigator, in accordance with the regulations of the medical institution implementing the study. Give the patient a copy of the consent form.
  4. If information that may affect the consent of study participants is obtained, or if changes are made to the study plan that may affect the consent of study participants, the representative investigator or sub-investigator shall convey the information promptly to the study participants. If there is a method specified by the medical institution implementing the study, records will be kept in accordance with that method. In addition, we will revise the explanatory documents and consent forms with prior approval from the CRB and obtain written consent again from the study participants.

## Instructions for study participants

The explanatory document shall include the following items:

1. The name of the specific clinical research to be implemented, the fact that the implementation of this specific clinical research has been approved by the administrator of the implementing medical institution, and the fact that the implementation plan has been submitted to the Minister of Health, Labor and Welfare.
2. The name of the implementing research institution and the name and title of the representative investigator (in the event that specific clinical research is conducted as a multi-center joint research, include the name and title of the principal investigator, the names of other implementing medical institutions, and the name and title of the representative investigator of the relevant medical institution).
3. Reasons for being selected as a subject for specific clinical research.
4. Anticipated benefits and disadvantages of implementing specific clinical research.
5. The fact that refusing to participate in a specific clinical research is voluntary.
6. Matters regarding withdrawal of consent.
7. The fact that refusing to participate in a specific clinical research or withdrawing consent shall not give participants any disadvantageous treatment.
8. Method of disclosing information regarding a specific clinical research.
9. The fact that study plans and other materials related to the implementation of specific clinical research can be obtained or viewed at the request of subjects of a specific clinical research or their legal representatives, along with the method of obtaining or viewing them.
10. Matters regarding the protection of personal information of subjects of a specific clinical research.
11. Methods for storing and disposing of samples, etc.
12. Status regarding the provision of research funds, etc. and other involvement in a specific clinical research.
13. System for responding to complaints and inquiries.
14. Matters related to costs in association with implementation of a specific clinical research.
15. Comparison with the existence and contents of other treatments and the expected benefits and disadvantages of other treatments.
16. Matters regarding compensation for health damages and provision of medical care due to the implementation of a specific clinical research.
17. Examination matters by the CRB that provide review opinions for a specific clinical research, along with other matters related to the CRB concerning this specific clinical research.
18. Other necessary matters regarding the implementation of a specific clinical research.

## Revision of explanatory and consent documents

In the event information is obtained that makes it necessary to revise the explanatory document or consent form, the principal investigator will promptly revise the explanatory document or consent form based on the information. In the event the principal investigator makes any revisions to the explanatory document or consent form, the principal investigator will submit them to the CRB specified in the implementation plan for approval. Each representative investigator will then receive approval from the administrator of the medical institution implementing the study.

## Withdrawal of consent

If a research participant requests to withdraw their consent, the representative investigator or sub-investigator will use a consent withdrawal form in order to confirm the details of the study participant's withdrawal of consent. Study participants will check the appropriate items regarding the content of withdrawing their consent on the consent withdrawal form, enter the date of withdrawal of consent, and sign it. The representative investigator or sub-investigator will check the contents of the consent withdrawal form, enter the date of confirmation, and sign it. The representative investigator or sub-investigator will issue a copy of the signed consent withdrawal form to the study participant, while the original will be kept at the relevant medical institution.

## Possibility of samples and information obtained from study participants being used for future studies

Samples and information obtained from study participants in this study may be used for future studies that will not be specified at the time consent is obtained. The remaining samples following the completion of the measurements at SRL Co., Ltd. (baseline, 8 weeks) will be appropriately stored at the Department of Cardiovascular Medicine, Saga University School of Medicine, until at least 5 years have passed following the discontinuation or completion of the research, then be disposed of properly in a state such that individuals cannot be identified. However, the remaining samples will be used only when new information related to this study is obtained and it is considered that additional analysis is necessary. Consent from study participants shall be obtained in advance, regarding the purpose of use and storage period of remaining samples. The samples remaining at 4 weeks will be appropriately stored and managed by SRL Co., Ltd. during the study period and promptly disposed of in accordance with SRL Co., Ltd.'s procedures once the study is completed.

If the donor withdraws their consent after obtaining consent for the storage and use of samples and information, after implementing the study, the samples and information associated with the study will be immediately disposed of in accordance with the wishes of the donor.

Additionally, if the samples and information obtained through this study are to be used in new studies planned in the future, a new study plan must be formulated and approved by an appropriate ethical review committee, in response to the content of the study. Data will then be provided to the study conductor in a form that does not allow individual study participants to be identified.

# **Research funding and conflicts of interest**

## Sources of funding and financial relationships

This study will be implemented with funding from Novartis Pharma Co., Ltd., based on an investigator-initiated clinical research agreement with Saga University. Furthermore, Otsuka Pharmaceutical Co., Ltd., an affiliated company of Novartis Pharma Co., Ltd., the manufacturer and distributor of sacubitril/valsartan (Enrest® Tablets), is not involved in this study. Responsibility for implementing the study rests with Saga University, which has entered into a contract with the Organization for Clinical Medicine Promotion, and will, in accordance with the contract, provide support related to monitoring, data management, statistical analysis, and coordination management. Novartis Pharma Co., Ltd. provides information about sacubitril/valsartan along with information and opinions on study plans, summary reports, etc., but is not involved in the implementation, analysis, interpretation or reporting of the results of the study. Furthermore, we will not refuse to publish study results, etc., unless there is a justifiable reason.

## Attribution of results

The results obtained in this study, other than the intellectual property related to sacubitril/valsartan, belong to the principal investigator and the medical institution to which the principal investigator belong.

## Managing conflicts of interest

The principal investigator will create conflict of interest management standards for the clinical research to be implemented and notify the representative investigator. The representative investigator will take into account the status of conflicts of interest as determined by the conflict of interest confirmation results (Researcher Conflict of Interest Self-Declaration Form) and the report from the administrator of the medical institution or the head of the affiliated institution (Conflict of Interest Situation Confirmation Report). Based on the content, each institution will create a conflict of interest management plan and submit it to the principal investigator. The principal investigator will listen to the opinions of the CRB regarding the conflict of interest management standards and conflict of interest management plan (including the case of changes). Conflicts of interest will be managed appropriately, in accordance with recognized conflict of interest management standards and the conflict of interest management plan.

## Conflict of interest status

Although the principal investigator receives personal income totaling more than 1 million yen per year from Novartis Pharma Co., Ltd., the results of this study will not be unduly influenced by this company. Although there are some representative investigators and sub-investigators who receive personal income totaling more than 1 million yen per year from Novartis Pharma Co., Ltd., the results of this study will not be unduly influenced by the company, as a result.

Although there are some representative investigators who earn a personal income of 2.5 million yen or more annually, the representative investigators or sub-investigators who earn a personal income of 1 million yen or more annually, and sub-investigators who earn lecture fees, from Otsuka Pharmaceutical Co., Ltd., the company in question is not involved in the study and therefore will not be unduly influenced.

# **Certified Review Board**

## Certified Review Board

This study will be reviewed and implemented by the following CRB.

Name: Fukushima Medical University, Certified Review Board (Certification number: CRB2200002)

Address: 1 Hikariga-oka, Fukushima City, Fukushima

Contact: fmucrb@fmu.ac.jp

TEL: 024-547-1825 (Certified Review Board office)

## Report to the CRB

## Regular report

6. Regular report to the CRB

The principal investigator shall report on the following matters to the CRB, regarding the implementation status of this study, every year starting from the date of submission of the implementation plan to the Minister of Health, Labor and Welfare, and within two months following the expiration of that period.

Additionally, when making regular reports, a report will also be made to the administrator of the medical institution implementing the study.

- 1. Number of study participants involved
  2. Occurrence status of diseases, etc. and subsequent progress
  3. Occurrence status of nonconformity and subsequent response
  4. Evaluation of safety and scientific validity
  5. Matters related to the involvement of drug manufacturers, etc. as stipulated in the Conflict of Interest Management Standards

1. Regular report to the Minister of Health, Labor and Welfare

The principal investigator will report the following matters to the Minister of Health, Labor and Welfare, within one month from the date on which the CRB stated its opinion regarding the implementation status of the study, which is described in the implementation plan.

- 1. Name of the CRB listed in the implementation plan
  2. Appropriateness of continuing the study by the CRB
  3. Number of study participants involved
  4. Occurrence status of diseases, etc. and subsequent progress
  5. Occurrence status of nonconformity and subsequent response
  6. Evaluation of safety and scientific validity
  7. Matters related to the involvement of drug manufacturers, etc. as stipulated in the Conflict of Interest Management Standards

## Other reports

During the implementation of the study, the principal investigator must report to the CRB in the following cases:

1. When he/she learns of the outbreak of a disease, etc.
2. When a major nonconformity is discovered
3. When creating the main evaluation item report or comprehensive report and the summary thereof

# **Changes to study plans, etc.**

When it becomes necessary to revise the study plan following the initiation of this study, the principal investigator will create in writing a revised study plan and a revision history thereof (contents of revisions and reasons therefor), as there is a possibility of changes to the implementation plan, then hear the opinion of the CRB.

Note that this excludes minor changes that fall under either 1) or 2) below:

1) Changes in the name of persons engaged in the clinical research that do not involve a change in the person engaged in clinical research (change in name due to change in marital status, etc.)

2) Changes due to changes in area name or lot number (location remains the same)

Upon receipt of a report on the revision of the study plan from the representative investigator, the principal investigator will submit the report to the review organization of the implementing medical institution as necessary (if the changes are deemed minor by the implementing medical institution, some items may be approved as report items).

In the event it is necessary to suspend the case registration during revision, the principal investigator will notify the representative investigator, sub-investigator, data center, and statistical analysis supervisor to that effect. Following the revision, the principal investigator will send the revised study plan or revised contents to the representative investigator and sub-investigator, the data center, and the person in charge of statistical analysis. The principal Investigator will revise the explanatory document to the study participants, in accordance with the revised study plan.

If the study is no longer to be continued at a specific medical institution during the course of this study, the principal investigator will submit changes made to the implementation plan, once the observation period for the study participants at that medical institution has completed.

# **Contents and methods of reporting to the administrator of the medical institution**

1. If the representative investigator obtains facts or information that impair the ethical validity or scientific rationality of the study, or information that may impair the study, he/she must promptly report to the administrator of the implementing medical institution, and if necessary, stop or discontinue the study, or change the study plan.
2. If the representative investigator obtains facts or information that impair the appropriateness of the implementation of the study or the credibility of the study results, or any information that may impair the credibility of the study, he/she must promptly report to the administrator of the implementing medical institution, and if necessary, stop or discontinue the study, or change the study plan.
3. When the representative investigator learns that the clinical research is not in compliance with ministerial ordinances or the study plan (nonconformity), he/she must promptly report this to the administrator of the medical institution and notify the principal investigator of this matter.
4. The representative investigator must report the progress of the study and the occurrence of diseases, associated with the implementation of the study, to the administrator of the medical institution, as specified in the study plan (once a year in principle).
5. When the representative investigator completes or discontinues the study, he/she must report the fact and provide a summary of the study results in writing to the administrator of the medical institution without delay.
6. When the representative investigator becomes aware of the occurrence of a serious disease or an infectious disease, suspected to be caused by the conduct of this study, but which cannot be predicted based on precautions for the use of the study drug, etc., he/she must report to the administrator of the implementing medical institution and then to the principal investigator. The principal investigator must promptly provide information to the other representative investigators accordingly.
7. When the principal investigator receives an opinion from the CRB, he/she must promptly report the opinion to the administrator of the medical institution and provide information to the other representative investigators accordingly. Other representative investigators who receive information from the principal investigator must promptly report the details of the provided information to the administrator of the medical institution.
8. When the principal investigator has prepared a main evaluation item report or a comprehensive report and a summary thereof, the principal investigator must obtain the opinion of the CRB in advance, and submit it to the administrator of the medical institution without delay and a summary of the main evaluation item report or comprehensive report must be published. When the principal investigator submits the summary of the comprehensive report, the principal investigator must submit the summary of the comprehensive report, the study plan, and the statistical analysis plan to the Minister of Health, Labor and Welfare, and promptly report to the administrator of the implementing medical institution, while simultaneously providing the information to other representative investigators. In this case, the other representative investigators must promptly report the details of the provided information to the administrator of the medical institution.
9. Upon completion of the study, the representative investigator must promptly take necessary measures to protect the human rights of study participants and their related parties, or the rights and interests of researchers and their related persons, after which the results of the study must be made public. Furthermore, when the final results have been published, they must be reported to the administrator of the study facility without delay.

# **Responses regarding the provision of medical care to study participants after study implementation**

There are no regulations regarding treatment following the completion of this study and no restrictions will be placed on continuing the administration of sacubitril/valsartan or the control drug or transitioning to another treatment following the completion of this study. The representative investigator and sub-investigator will strive to ensure that study participants receive the best prevention, diagnosis, and treatment even after the study ends.

# **References and literature list**

1. Japanese Circulation Society. Report on the Actual Condition of Cardiovascular Disease Treatment (conducted and published in 2016): JROAD (The Japanese Registry Of All cardiac and vascular Diseases). Tokyo: Japanese Circulation Society. Available from: <http://www.j-circ.or.jp/jittai_chosa/jittai_chosa2015web.pdf> [Accessed Mar 2 2021].
2. Ministry of Health, Labour and Welfare. Summary of Vital Statistics 2014 (Final Figures). Tokyo: Ministry of Health, Labour and Welfare. Available from <http://www.mhlw.go.jp/toukei/saikin/hw/jinkou/kakutei14/index.html> [Accessed Mar 2 2021].
3. Study Group on the Ideal System for the Provision of Medical Care for Stroke, Heart Disease, and Other Cardiovascular Diseases: The ideal system for the provision of medical care for stroke, heart disease, and other cardiovascular diseases (July 2009). Tokyo: Ministry of Health, Labour and Welfare. Available from: <http://www.mhlw.go.jp/file/05-Shingikai-10901000-Kenkoukyoku-Soumuka/0000173149.pdf> [Accessed Mar 2 2021].
4. Krumholz HM, Lin Z, Keenan PS, Chen J, Ross JS, Drye EE, et al. Relationship between hospital readmission and mortality rates for patients hospitalized with acute myocardial infarction, heart failure, or pneumonia. JAMA. 2013;309(6):587-93.
5. Dharmarajan K, Hsieh AF, Lin Z, Bueno H, Ross JS, Horwitz LI, et al. Diagnoses and timing of 30-day readmissions after hospitalization for heart failure, acute myocardial infarction, or pneumonia. JAMA. 2013;309(4):355-63.
6. Ross JS, Chen J, Lin Z, Bueno H, Curtis JP, Keenan PS, et al. Recent national trends in readmission rates after heart failure hospitalization. Circ Heart Fail. 2010;3(1):97-103.
7. Tanaka A, Node K. Who Will Be Rehospitalized next?: Targeting heart failure patients with brittle dimensions. JACC Heart Fail. 2017;5(10):760-1.
8. Greene SJ, Fonarow GC, Vaduganathan M, Khan SS, Butler J, Gheorghiade M. The vulnerable phase after hospitalization for heart failure. Nat Rev Cardiol. 2015;12(4):220-9.
9. Bhagat AA, Greene SJ, Vaduganathan M, Fonarow GC, Butler J. Initiation, continuation, switching, and withdrawal of heart failure medical therapies during hospitalization. JACC Heart Fail. 2019;7(1):1-12.
10. Solomon SD, Zile M, Pieske B, Voors A, Shah A, Kraigher-Krainer E, et al. The angiotensin receptor neprilysin inhibitor LCZ696 in heart failure with preserved ejection fraction: a phase 2 double-blind randomised controlled trial. Lancet. 2012;380(9851):1387-95.
11. McMurray JJ, Packer M, Desai AS, Gong J, Lefkowitz MP, Rizkala AR, et al. Angiotensin-neprilysin inhibition versus enalapril in heart failure. N Engl J Med. 2014;371(11):993-1004
12. Solomon SD, McMurray JJV, Anand IS, Ge J, Lam CSP, Maggioni AP, et al. Angiotensin-neprilysin inhibition in heart failure with preserved ejection fraction. N Engl J Med. 2019;381(17):1609-20.
13. Tsutsui H, Momomura S, Saito Y, Ito H, Yamamoto K, Ohishi T, et al. Efficacy and safety of sacubitril/valsartan (LCZ696) in Japanese patients with chronic heart failure and reduced ejection fraction: Rationale for and design of the randomized, double-blind PARALLEL-HF study. J Cardiol. 2017;70(3):225-31.
14. Januzzi JL, Jr., Prescott MF, Butler J, Felker GM, Maisel AS, McCague K, et al. Association of change in N-terminal pro-b-type natriuretic peptide following initiation of sacubitril-valsartan treatment with cardiac structure and function in patients with heart failure with reduced ejection fraction. JAMA. 2019;322(11):1-11.
15. Solomon SD, Vaduganathan M, B LC, Packer M, Zile M, Swedberg K, et al. Sacubitril/valsartan across the spectrum of ejection fraction in heart failure. Circulation. 2020;141(5):352-61.
16. Velazquez EJ, Morrow DA, DeVore AD, Duffy CI, Ambrosy AP, McCague K, et al. Angiotensin-Neprilysin Inhibition in Acute Decompensated Heart Failure. N Engl J Med. 2019;380(6):539-48.
17. Morrow DA, Velazquez EJ, DeVore AD, Desai AS, Duffy CI, Ambrosy AP, et al. Clinical Outcomes in Patients With Acute Decompensated Heart Failure Randomly Assigned to Sacubitril/Valsartan or Enalapril in the PIONEER-HF Trial. Circulation. 2019;139(19):2285-8.
18. ClinicalTrials.gov [Internet]. Bethesda (MD): National Library of Medicine (US). Identifier NCT03988634, Changes in NT-proBNP and outcomes, safety, and tolerability in hfpef patients with acute decompensated heart failure (ADHF) who have been stabilized during hospitalization and initiated in-hospital or within 30 days post-discharge (PARAGLIDE-HF); 17 Jun 2019. Available from: https://clinicaltrials.gov/ct2/show/NCT03988634 [Accessed Mar 2 2021].
19. Vaduganathan M, Claggett BL, Desai AS, Anker SD, Perrone SV, Janssens S, et al. Prior heart failure hospitalization, clinical outcomes, and response to sacubitril/valsartan compared with valsartan in HFpEF. J Am Coll Cardiol. 2020;75(3):245-54.

# **Amendment**

1. First amendment created on Dec. 1, 2021

| (Before change) Creation date: September 9, 2021 | (After change) Creation date: December 1, 2021 | Reasons for change |
| --- | --- | --- |
| Sep. 9, 2021 ver1.1 | Dec. 1, 2021 ver1.2 | For revision |
| Creation Date: Sep. 1, 2021  Version number: Version 1.1 | Creation Date: Dec. 1, 2021  Version number: Version 1.2 | For revision |
| Revision history  Version number, Creation/Revision date, Reason for revision  Version 1.0 (first version) June 30, 2021 Newly created  Version 1.1 September 9, 2021 Due to corrections related to CRB deliberations | Revision history  Version number, Creation/Revision date, Reason for revision  Version 1.0 (first version) June 30, 2021 Newly created  Version 1.1 September 9, 2021 Due to corrections related to CRB deliberations  Version 1.2 December 1, 2021　For description improvement, etc. | For revision |
| 2. Study contents  2.6 Randomization procedure  Using the central registration method, information will be immediately confirmed on the EDC website, and if eligible, a case registration number will be assigned and protocol treatment allocated on the EDC.  Allocation to the sacubitril/valsartan group and the control group will be made at a 1:1 ratio using the following allocation adjustment factors.  [Allocation factors]  Age upon obtaining consent (under 70, over 70)  Sex (female, male)  LVEF (less than 40%, more than 40%)*  Atrial fibrillation (presence or absence)  eGFR (less than 60 mL/min/1.73 m^2^, more than 60 mL/min/1.73 m^2^)  Facility  Priority will be given to data obtained during the current hospitalization; however, if data cannot be obtained, data from within 3 months prior to obtaining consent may also be used. | 2. Study content  2.6 Randomization procedure  Using the central registration method, information will be immediately confirmed on the EDC website, and if eligible, a case registration number will be assigned and protocol treatment dynamically allocated on the EDC.  Dynamic allocation to the sacubitril/valsartan group and the control group will be made at a 1:1 ratio with the minimization method, using the following allocation adjustment factors.  [Allocation factors]  Age upon obtaining consent (under 70, over 70)  Sex (female, male)  LVEF (less than 40%, more than 40%)**  Atrial fibrillation (presence or absence)*  eGFR (less than 60 mL/min/1.73 m^2^, more than 60 mL/min/1.73 m^2^)*  Facility  * Data obtained during the current hospitalization may be used.  ** Priority will be given to data obtained during the current hospitalization; however, if data cannot be obtained, data from within 3 months prior to obtaining consent may also be used. | For description improvement. |
| 4. Treatment for study subjects  4.1 Observation/examination schedule  Table 1 Observation/Examination schedule  3) For the LVEF of the allocation factors, priority will be given to data obtained during the current hospitalization; however, if data cannot be obtained, data from within 3 months prior to obtaining consent may also be used. | 4. Treatment for study subjects  4.1 Observation/Examination schedule  Table 1 Observation/Examination schedule  3) For the LVEF of the allocation factors, priority will be given to data obtained during the current hospitalization; however, if data cannot be obtained, data from within 3 months prior to obtaining consent may also be used. Furthermore, for the presence or absence of atrial fibrillation as well as the eGFR, data obtained during the current hospitalization may be used. | For description improvement. |
| 4. Treatment for study subjects  4.2.1 Investigation items at the time of registration/allocation  After determining eligibility, record the following upon registration/allocation and enter it into the EDC.  ・Date of obtaining consent, age when obtaining consent, date of birth, sex  ・Presence or absence of atrial fibrillation, LVEF*, eGFR    Priority will be given to data obtained during the current hospitalization; however, if data cannot be obtained, data from within 3 months prior to obtaining consent may also be used. | 4. Treatment for study subjects  4.2.1 Investigation items at the time of registration/allocation  After determining eligibility, record the following upon registration/allocation and enter it into the EDC.  ・Date of obtaining consent, age when obtaining consent, date of birth, sex  ・Presence or absence of atrial fibrillation*, LVEF**, eGFR*    　* Data obtained during the current hospitalization may be used.  ** Priority will be given to data obtained during the current hospitalization; however, if data cannot be obtained, data from within 3 months prior to obtaining consent may also be used. | For description improvement. |
| 5. Efficacy and safety evaluation  5.1 Efficacy evaluation items  Secondary evaluation items  3. Percentage of patients with NT-proBNP levels reduced by 30% or more from the baseline at 4 weeks following protocol treatment initiation, Cardiac troponin | 5. Efficacy and safety evaluation  5.1 Efficacy evaluation items  Secondary evaluation items  3. Proportion of cases in which the NT-proBNP value decreased by 30% or more from the baseline at 4 weeks following protocol treatment initiation | For typo correction. |

1. Second amendment created on Jan. 10, 2023

| (Before change) Creation date: December 1, 2021 | (After change) Creation date: January 10, 2023 | Reasons for change |
| --- | --- | --- |
| Dec. 1, 2021 ver 1.2 | Jan. 10, 2023 ver 1.3 | For revision |
| Creation Date: Dec. 1, 2021 | Creation Date: Jan. 10, 2023  Version number: Version 1.3 | For revision |
| Revision history  Version number, Date of creation/date of revision, Reason for revision  Version 1.0 (first version), June 30, 2021 Newly created  Version 1.1 September 9, 2021 Due to corrections related to CRB deliberations  Version 1.2 December 1, 2021 For description improvement, etc. | Revision History  Version number, Creation/Revision date, Reason for revision  Version 1.0 (first version) June 30, 2021, Newly created  Version 1.0 September 9, 2021 For corrections pertaining to CRB deliberation  Version 1.2 December 1, 2021 For description improvement, etc.  Version 1.3 January 10, 2023 Due to extension of case registration period | For revision |
| **Study overview**  Study period  Case registration period: Clinical study implementation plan/study summary publication system (jRCT) Release date - March 31, 2023 | **Study overview**  Study period  Case registration period: Clinical study implementation plan/study summary publication system (jRCT) Release date - June 30, 2023 | Due to extension of case registration period. |
| **15. Study implementation period**  15.1. Study implementation period  Case registration period: Clinical study implementation plan/study summary publication system (jRCT) Release date - March 31, 2023 | **15. Study implementation period**  15.1. Study implementation period  Case registration period: Clinical study implementation plan/study summary publication system (jRCT) Release date - June 30, 2023 | Due to extension of case registration period. |

**Inclusion and exclusion criteria**

| **Inclusion** | **Exclusion** |
| --- | --- |
| 1. Patients who provided written informed consent to participate in this study 2. Patients who are 20 years of age or older at the time of consent (regardless of sex) 3. Hospitalized due to worsening heart failure, irrespective of left ventricular ejection fraction, with both symptoms of heart failure, such as dyspnea at rest or on mild exertion, and signs of congestion, such as edema, moist rales, and congestion on chest radiography 4. NYHA functional class II to IV 5. Taking an ACEI or an ARB medication 6. Can undergo randomization within 7 days of index hospitalization 7. Patients who meet the criteria of hemodynamic stability defined as follows: 8. Systolic blood pressure of 100 mm Hg or more 9. No increase in intravenous diuretic agent use within the last 6 h before randomization 10. No intravenous administration of vasodilators or inotropic agents 11. Patients who meet the following reference range for natriuretic peptide level from 48 h before index hospitalization to the time of eligibility assessment;   NT-proBNP concentration of 1200 pg/mL or more  or  BNP concentration of 300 pg/mL or more | 1. Currently taking oral Sac/Val medication or have taken it within 30 days prior to randomization 2. History of hypersensitivity to ingredients in Sac/Val, ACEI, or ARB; or expected to be contraindicated for or intolerant to any of these drugs 3. History of angioedema 4. Severe renal dysfunction (eGFR < 30 mL/min/1.73m^2^), on maintenance dialysis, or known bilateral renal artery stenosis (in patients with solitary kidney; known renal artery stenosis in the residual kidney) 5. Severe liver dysfunction (Child-Pugh class C) 6. Patients with diabetes who are currently taking aliskiren fumarate 7. Serum potassium level of 5.3 mEq/L or more 8. Cardiogenic shock 9. On mechanical cardiopulmonary support, left ventricular assist device, or ventilator 10. Onset of acute coronary syndrome or stroke within 30 days prior to randomization 11. History of surgical or percutaneous treatment of cardiovascular diseases within 30 days prior to randomization 12. Patients with planned coronary artery revascularization or surgical or percutaneous treatment of cardiovascular diseases during an individual observation period 13. Patients with planned electrical cardioversion, cardiac resynchronization therapy, or pacemaker implantation during an individual observation period 14. History or comorbidity of hypertrophic obstructive cardiomyopathy or infiltrative cardiomyopathy, such as amyloidosis or sarcoidosis 15. Active pericardial disease 16. History of or awaiting heart transplantation 17. Active infectious disease or severe chronic respiratory disease 18. Patients who are or might become pregnant or who are breastfeeding 19. Patients considered unsuitable for the study by a study investigator, such as patients with comorbid active malignancy |

ACEI, angiotensin-converting enzyme inhibitor; ARB, angiotensin receptor blocker; BNP, B-type natriuretic peptide; eGFR, estimated glomerular filtration rate; NT-proBNP, N-terminal pro-B-type natriuretic peptide; NYHA, New York Heart Association; Sac/Val, sacubitril/valsartan.

**Study design**


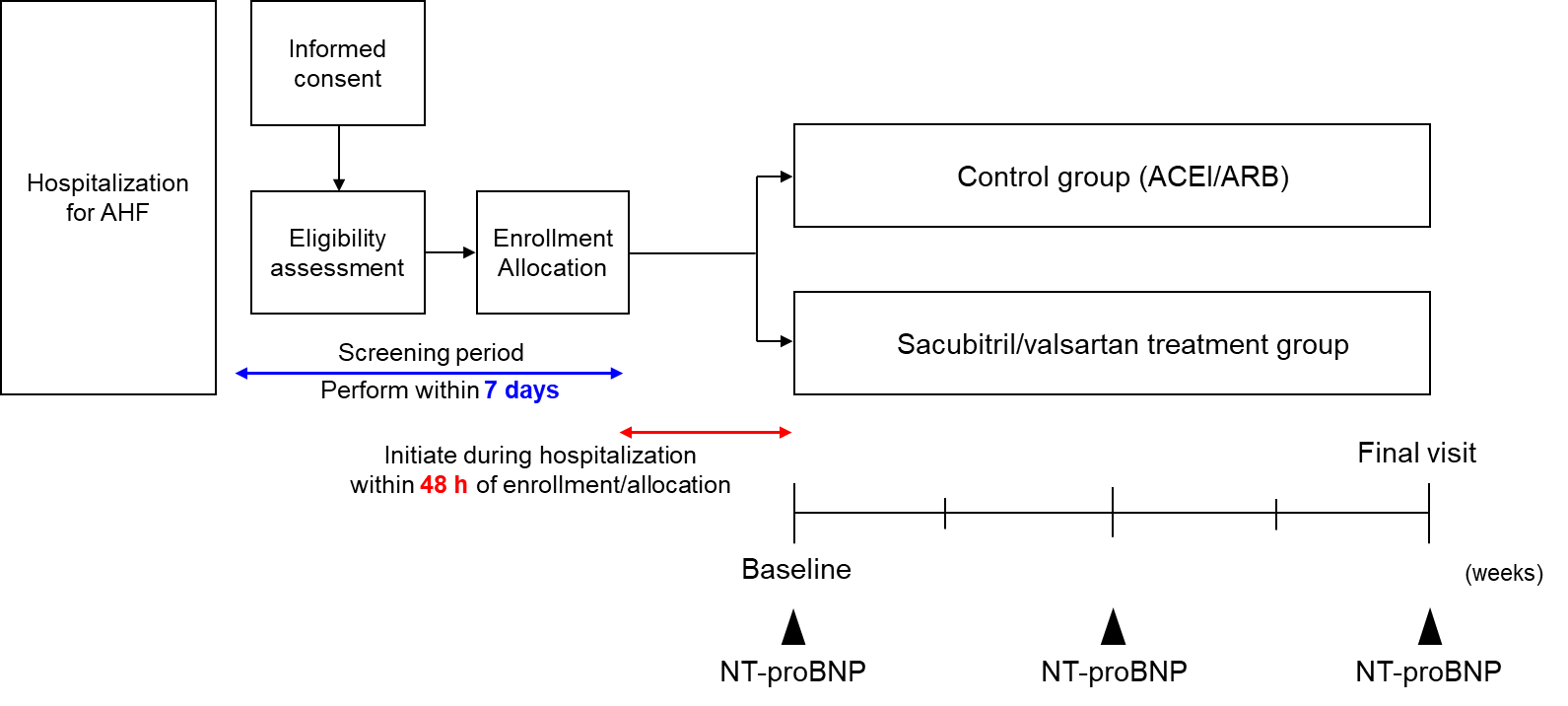


ACEI/ARB, angiotensin-converting enzyme inhibitor or angiotensin receptor blocker; AHF, acute heart failure; NT-proBNP, N-terminal pro-B-type natriuretic peptide.

**Dose adjustment protocol**

**
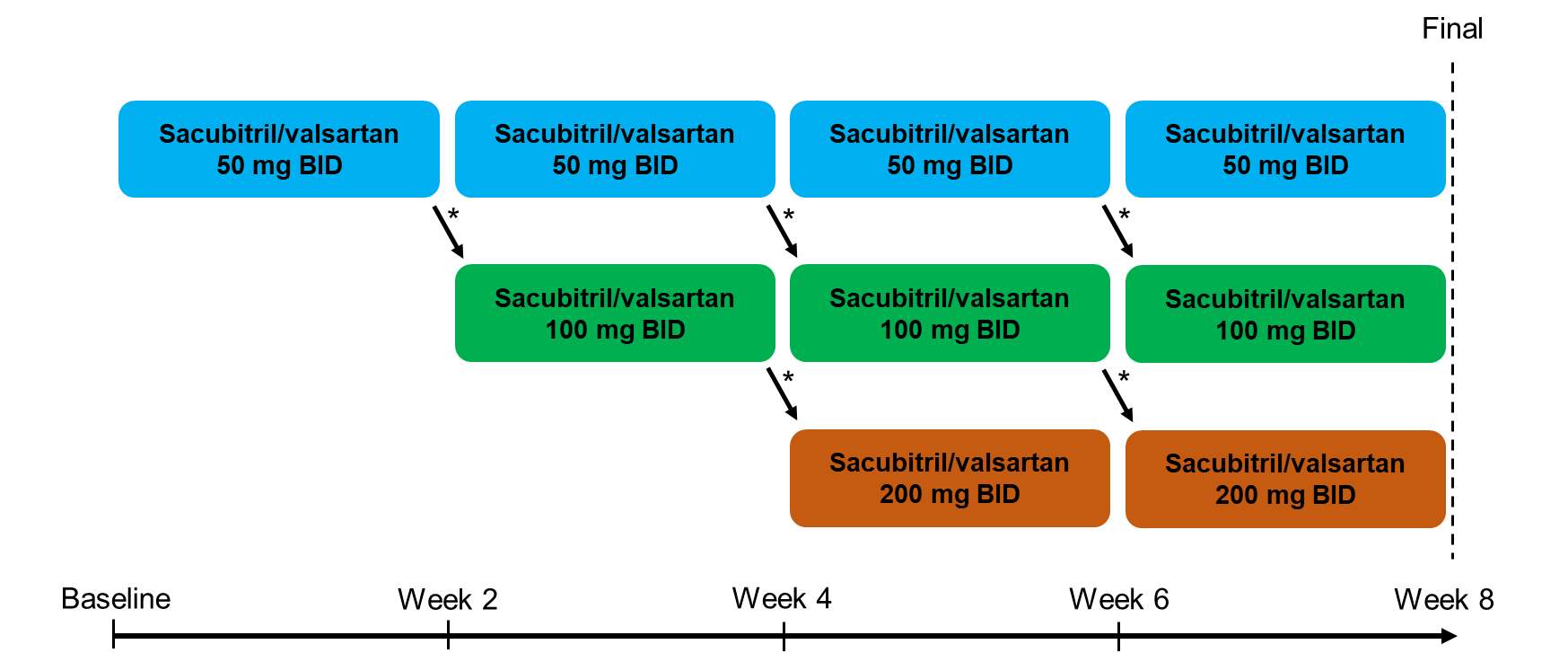
**

*The safety and tolerability of sacubitril/valsartan were evaluated using the following criteria: i) Systolic blood pressure of 95 mm Hg or higher without symptomatic hypotension, ii) Serum potassium level of 5.4 mEq/L or less, iii) eGFR of 30 mL/min/1.73m^2^ or more, and iv) The most recent decline in eGFR of 35% or less. When tolerated, the doses of sacubitril/valsartan were titrated to the next doses of sacubitril/valsartan. Importantly, the timing of dose increase of sacubitril/valsartan was not limited only to weeks 2, 4, and 6 time points shown in the figure.

**Prespecified clinical outcomes**

1. Time to first occurrences of the composite event of worsening heart failure event, defined as (i) unplanned rehospitalization; (ii) initiation of intravenous treatment (vasodilator or inotropic agent) for heart failure during index hospitalization, excluding at rehospitalization; (iii) urgent visit due to heart failure requiring intravenous treatment (vasodilator, inotropic agent, or diuretic); or (iv) initiation of oral diuretic (loop diuretic, thiazide-type diuretic, or tolvaptan), at least a 50% increase in its dose (outpatient), or all-cause death.
2. Occurrences of the composite event of first and recurrent worsening heart failure events or all-cause death.
3. Occurrences of the individual events of first and recurrent worsening heart failure events, all-cause death, and cardiovascular death.

**Pr**ogram of Angiot**e**nsin-Neprilysin Inhibition in Ad**m**itted Pat**ie**nts with Wo**r**sening Heart Failure

PREMIER study

Statistical analysis plan

Principal investigator

From: Department of Cardiovascular Medicine, Saga University Hospital

Name: Kohichi Node

Head of statistical analysis

From: Clinical Research Division, Organization for Clinical Medicine Promotion

Name: Takumi Imai

Creation date: Dec. 1, 2023 (Ver 1.1)

Creation/Revision History

| Version number | Creation/Revision Date | Creation/Revision Contents | Created by |
| --- | --- | --- | --- |
| 1.0 | Jun. 13, 2023 | First version created | Takumi Imai |
| 1.1 | Dec. 1, 2023 | Minor revision | Takumi Imai |
|  |  |  |  |

**Contents**

1. Purpose of this plan 87

1.1 Changes from the study plan 87

1.2 Abbreviation definitions 87

2. Study overview 89

2.1 Study purpose 89

2.2 Study drug 89

2.3 Study design 89

2.4 Study subjects 89

2.5 Target number of cases 89

2.6 Study method 90

3. Analysis set 90

3.1 Efficacy analysis set 90

3.1.1 Randomized set (Randomized) 90

3.1.2 Full analysis set (FAS) 90

3.1.3 Per-protocol set (PPS) 90

3.2 Safety set (SS) 90

4. Endpoints 91

4.1 Primary endpoint 91

4.2 Secondary endpoints 91

4.3 Safety endpoints 92

5. Data handling 92

5.1 Handling of missing values 92

5.2 Handling of test at the time of discontinuation 92

5.3 Definition of calculated variables 92

6. Statistics to be output and significance level for hypothesis testing 93

6.1 Descriptive statistics 93

6.2 Inferential statistics 93

6.3 Analysis using linear models and linear mixed models (MMRM) 94

6.4 Frequency aggregation 94

7. Statistical analysis 94

7.1 Subject classification and breakdown 94

7.2 Demographic and clinical characteristics 95

7.3 Description of medication status 95

7.4 Primary endpoint 96

7.5 Secondary endpoints 96

7.6 Subgroup analysis for primary endpoint 100

7.7 Safety endpoints 101

8. Statistical analysis and tabulation software 102

9. References 102

10. Amendment 103

# **Purpose of this plan**

The purpose of this statistical analysis plan is to clarify and specify the details of the statistical analysis plan specified in the study plan on the implementation method of statistical analysis in the “Study on Angiotensin-Neprilysin Inhibitors in Hospitalized Patients with Worsening Heart Failure (PREMIER study)” (hereinafter, referred to as “the study”).

## Changes from the study plan

None

## Abbreviation definitions

| Abbreviation | Full expression (English) | Full expression (Japanese) |
| --- | --- | --- |
| ACE | angiotensin converting enzyme | Angiotensin converting enzyme |
| ARB | angiotensin II receptor blocker | Angiotensin receptor blocker |
| BMI | body mass index | Body mass index |
| CABG | coronary artery bypass grafting | Coronary artery bypass grafting |
| COPD | chronic obstructive pulmonary disease | Chronic obstructive pulmonary disease |
| Cr | creatinine | Creatinine |
| CRP | C-reactive protein | C-reactive protein |
| CRT | cardiac resynchronization therapy | Cardiac resynchronization therapy |
| E/e’ | ― | Ratio of early diastolic mitral inflow velocity to early diastolic mitral annulus velocity |
| eGFR | estimated glomerular filtration rate | Estimated glomerular filtration rate |
| FAS | full analysis set | Full analysis set |
| GDF-15 | growth differentiation factor 15 | Growth differentiation factor 15 |
| GLS | global longitudinal strain | Index of myocardial longitudinal contractile function |
| HFpEF | heart failure with preserved ejection fraction | Diastolic heart failure |
| HFrEF | heart failure with reduced ejection fraction | Heart failure with reduced ejection fraction |
| Ht | hematocrit | Hematocrit |
| ICD | implantable cardiac defibrillator | Implantable cardiac defibrillator |
| IVC | inferior vena cava | Inferior vena cava diameter |
| KCCQ-12 | kansas city cardiomyopathy questionnaire-12 | Kansas city cardiomyopathy questionnaire-12 |
| LAVI | left atrial volume index | Left atrial volume index |
| LLT | lowest level term | Lowest level term |
| LVEDV | left ventricular end-diastolic volume | Left ventricular end-diastolic volume |
| LVEF | left ventricular ejection fraction | Left ventricular ejection fraction |
| LVESV | left ventricular end-systolic volume | Left ventricular end-systolic volume |
| LVMI | left ventricular mass index | Left ventricular mass index |
| LVOT | left ventricular outflow tract | Left ventricular outflow tract diameter |
| LVOT-VTI | left ventricular outflow tract velocity time integral | Left ventricular outflow tract velocity time integral |
| MedDRA/J | Medical Dictionary for Regulatory Activities/Japanese version | Medical Dictionary for Regulatory Activities/Japanese version |
| MMRM | mixed-effects models for repeated measures | Mixed model for repeated measures |
| MRA | mineralocorticoid receptor antagonist | Mineralocorticoid receptor antagonist |
| NT-proBNP | N-terminal fragment of pro-B-type natriuretic peptide | N-terminal fragment of pro-B-type natriuretic peptide |
| NYHA | New York Heart Association | New York Heart Association |
| PCI | percutaneous coronary intervention | Percutaneous coronary intervention |
| PPS | per protocol set | Per protocol set |
| PT | preferred term | Basic terminology |
| septal e’､lateral e’ | ― | Septal and lateral mitral annulus movement speed |
| SGLT2 | sodium glucose cotransporter 2 | Sodium glucose cotransporter 2 |
| SOC | system organ class | System organ class |
| SS | safety set | Safety set |
| Soluble ST2 | soluble suppression of tumorigenesis-2 | Soluble interleukin 1 receptor family |
| TEAE | treatment-emergent adverse event | Adverse events that occurred following protocol treatment initiation |
| TR velocity | tricuspid regurgitation velocity | maximum blood flow velocity for tricuspid regurgitation velocity |
| 1.5AG | 1.5-anhydro-D-glucitol | 1.5-anhydro-D-glucitol |

# **Study overview**

## Study purpose

To examine the effects of sacubitril/valsartan compared with the standard treatment for hospitalized patients with worsening heart failure (HF), using changes in NT-proBNP as an indicator.

## Study drug

Sacubitril/valsartan sodium hydrate (brand name: Entresto® Tablets).

## Study design

Investigator-initiated, multicenter, prospective, parallel-group, randomized controlled trial.

## Study subjects

Patients hospitalized due to worsening HF with HF symptoms and signs of congestion.

## Target number of cases

400 cases (sacubitril/valsartan group: 200 cases, control group: 200 cases).

## Study method

Upon confirming the eligibility of patients who have provided their consent, all patients who meet the eligibility criteria will be enrolled. Patients will be randomized into two groups: a sacubitril/valsartan group or a control group (sacubitril/valsartan non-treated group) within 7 days of hospitalization, after protocol treatment initiation during hospitalization, within 48 hours of enrollment and allocation, followed by 8 weeks of observation and testing.

# **Analysis set**

## Efficacy analysis set

The main analysis will be analysis using a full analysis set (FAS). Analysis using a per-protocol set (PPS) will be conducted as a complementary method in order to confirm the robustness of the results.

### Randomized set (Randomized)

This is the group to which allocation was implemented after registration.

### Full analysis set (FAS)

In accordance with the intention-to-treat principle, the full analysis set (FAS) excluding the following cases, will be the efficacy set:

- Cases in which consent was withdrawn after registration.
- Cases found to be ineligible after registration.
- Cases that have not received any protocol treatment after allocation.
- Cases without data regarding efficacy after protocol treatment initiation.

### Per-protocol set (PPS)

Among FAS, the patient population without major protocol deviations will be the per-protocol set (PPS) and will be the set for supplementary efficacy analysis.

The data center will evaluate protocol deviations as the number of cases accumulates, hold case review meetings between the principal research physician, the person in charge of statistical analysis, and the data center before locking in the research data, and establish how to handle any major protocol deviations and cases.

## Safety set (SS)

Randomly allocated patients who have received at least one protocol treatment will be considered as the safety set (SS).

# **Endpoints**

## Primary endpoint

Between-group ratio of the proportional change in the geometric mean NT-proBNP at 8 weeks following protocol treatment initiation, compared with baseline.

## Secondary endpoints

1. Between-group ratio of the proportional change in geometric mean NT-proBNP at 4 weeks following protocol treatment initiation, compared with baseline.
2. Percentage of cases in which the NT-proBNP value decreased by 50% or more from the baseline, at 8 weeks following protocol treatment initiation.
3. Percentage of cases in which the NT-proBNP value decreased by 30% or more from the baseline, at 4 weeks following protocol treatment initiation.
4. Percentage of cases which mean NT-proBNP value decreased by 40% or more from the baseline at 4 and 8 weeks following protocol treatment initiation.
5. Changes from the baseline of cardiac troponin T, CRP, GDF-15, soluble ST2, glycoalbumin, and 1.5AG at 8 weeks after following protocol treatment initiation.
6. Changes from the baseline of weight, BMI, blood pressure, pulse rate, laboratory test values, and NYHA class at the 4 and 8 weeks following protocol treatment initiation.
7. Changes from the baseline of cardiac function indicators (LVEDV, LVESV, LVEF, septal e', lateral e', mitral orifice blood flow velocity waveform (E), E/e', LVMI, LAVI , LVOT, LVOT-VTI, TR velocity, IVC, GLS, Left atrial strain (2-chamber view and 4-chamber view), at 8 weeks following protocol treatment initiation, along with the percentage of cases with a respiratory variation of 50% or more in IVC.
8. Changes from the baseline of KCCQ-12 at 8 weeks following protocol treatment initiation, along with the percentage of cases whose score increased by 5 points or more.
9. Time to composite outcomes of first HF event [the following events due to the exacerbation of HF: i) unplanned rehospitalization ii) initiation of intravenous treatment for HF (vasodilators, inotropes) (during hospitalization: excluding rehospitalization); iii) emergency visit for HF requiring intravenous therapy (vasodilators, inotropes, diuretics); and iv) initiation of oral diuretics (loop diuretics, thiazide diuretics, tolvaptan) or a dose ;increase of ≥50% (outpatient)] and all-cause mortality.
10. Number and frequency of composite outcomes of HF events including recurrence and all-cause mortality.
11. Number and frequency of following individual events: first or recurrent HF events, all-cause mortality, and cardiovascular death.
12. Number of occurrences and time to onset of specific adverse events, such as worsening of renal function (more than 50% increase in serum Cr or more than 30% decrease in eGFR), hyperkalemia (serum potassium 5.5 mEq/L or more), symptomatic hypotension, and angioedema.
13. Number of occurrences of other serious adverse events.

## Safety endpoints

Adverse events that occurred following the protocol treatment initiation

# **Data handling**

## Handling of missing values

Unless otherwise specified, in the event of any missing values, they will be treated as missing values without being imputed with other data and will not be included in aggregation or descriptive statistics calculations.

## Handling of test at the time of discontinuation

In the safety analysis, data from the test at the time of discontinuation will be treated as data from the most recent test after the test point specified, before the implementation of the test at the time of discontinuation. If the test at the time of discontinuation is implemented after the specified final test point, the data will not be included in the aggregation and descriptive statistics calculation.

## Definition of calculated variables

The change in variable A at a certain point in time t is defined by the following formula:

Change in variable A = variable A (t) - variable A (baseline)

The proportional change in variable A at a certain point in time t is defined by the following formula:

Proportional change in variable A = variable A (t) / variable A (baseline)

The proportional change in the geometric mean of NT-proBNP is calculated by the following formula:

Proportional change of NT-proBNP geometric mean (t) = NT-proBNP geometric mean (t) / NT-proBNP geometric mean (baseline)

= exp(ln(NT-proBNP) mean (t) - ln(NT-proBNP) mean (baseline))

= exp(ln(NT-proBNP) mean change)

Change in NYHA class at time t (worse, unchanged, improved)

- Worsening: NYHA class (t) > NYHA class (baseline)
- Unchanged: NYHA class (t) = NYHA class (baseline)
- Improvement: NYHA class (t) < NYHA class (baseline)

Respiratory fluctuations of 50% or more in IVC

- Inspiratory capacity size is less than half the expiratory capacity size: Presence of respiratory fluctuation of 50% or more in IVC.
- Inspiratory size is more than half the expiratory capacity size: No respiratory fluctuation of 50% or more in IVC.

The incidence of adverse events is calculated using the following formula:

Adverse event incidence (%) = (number of cases with adverse events / number of cases subject to safety analysis) × 100

# **Statistics to be output and** **significance level for hypothesis testing**

The statistics to be included in the statistical analysis report shall be as follows:

## Descriptive statistics

Output the number of samples, mean value, standard deviation, minimum value, 25% point, median value, 75% point, maximum value, and number of missing values.

## Inferential statistics

Output the mean value and 95% confidence interval based on the t-distribution. Regarding variables in which the normality of the residuals is not considered possible, calculate the mean value on the natural logarithm scale and the 95% confidence interval based on the t distribution, perform antilogarithmic transformation, and output the values back to the original scale.

## Analysis using linear models and linear mixed models (MMRM)

Output the estimation result of the group effect (estimated value, 95% confidence interval) and the P value of the hypothesis test for the null hypothesis of "no group effect". The significance level of the test shall be 5%. Regarding variables in which normality of residuals is not considered possible, analysis is performed on a natural logarithmic scale, with estimated values and 95% confidence intervals output as values returned to the original scale by antilogarithmic transformation.

## Frequency aggregation

Output the number of cases and percentage (%). If necessary, output the 95% confidence interval of the proportion using the Clopper-Pearson method.

# **Statistical analysis**

## Subject classification and breakdown

The following numbers will be counted for cases in which consent was obtained.

- Number of consent-obtained cases
- Number of registered cases
- Number of unregistered cases
- Number of allocated cases
- Number of unallocated cases
- Number of SS cases in each treatment group
- Number of cases excluded from SS in each treatment group
- Number of FAS cases in each treatment group
- Number of cases excluded from FAS in each treatment group
- Number of PPS cases in each treatment group
- Number of cases excluded from PPS in each treatment group
- Number of cases completing the 8-week follow-up in each FAS treatment group
- Number of cases missing the 8-week follow-up in each FAS treatment group

For non-allocated cases, list the reasons why allocation was not implemented. For cases excluded from SS among allocated cases, cases excluded due to FAS among SS, and for cases excluded due to PPS among FAS, a breakdown of the reasons for exclusion will be described in a table. For cases which did not complete the 8-week follow-up in each treatment group, a breakdown of the reasons for not completing the follow-up will be listed in a table.

## Demographic and clinical characteristics

For each analysis set (Randomized, FAS, PPS, SAS), descriptive statistics and frequency aggregation of the following variables will be performed for the entire set and for each treatment group.

- Age
- Sex
- Presence or absence of atrial fibrillation
- LVEF
- LVEF (less than 40%, more than 40%)
- eGFR
- Period from hospitalization to registration/allocation
- Height
- Weight
- BMI
- Blood pressure (systolic/diastolic)
- Pulse rate
- Presence or absence of complications/pre-existing conditions
- Causes of HF (ischemic, non-ischemic)
- NYHA classification (II-IV)
- Concomitant drugs
- Existing combination therapy

The breakdown of complications and pre-existing conditions is as follows:

Hypertension, diabetes, dyslipidemia, ischemic heart disease, ischemic stroke, atrial fibrillation, HF [Yes: less than 1.5 years or more than 1.5 years], hospitalization due to HF, COPD

The breakdown of concomitant drugs is as follows:

ACE inhibitors or ARBs, beta blockers, calcium channel blockers, MRAs, diuretics, digitalis preparations, SGLT2 inhibitors, ivabradine, statins

The breakdown of existing combination therapies is as follows:

PCI, CABG, pacemaker, ICD, CRT

## Description of medication status

Targeting FAS, descriptive statistics and frequency tabulations will be performed on the sacubitril/valsartan dose and other medication status in the sacubitril/valsartan group, along with the medication status in the control group.

## Primary endpoint

As the primary analysis, perform the following analyses targeting FAS: Also perform analyses targeting PPS in order to confirm the robustness of the results.

1. Calculate the descriptive statistics for NT-proBNP and its change for each treatment group and evaluation period. Calculate the inferential statistics for the geometric mean and proportional change in the geometric mean of NT-proBNP for each treatment group and evaluation period.
2. Plot the inferential statistics of the geometric mean and proportional change in the geometric mean of NT-proBNP for each treatment group and evaluation period. The Y axis may be displayed as a common logarithmic scale.
3. Perform an analysis of the mixed-effects model for repeated measurements (MMRM) for the logarithm of NT-proBNP values at 4 and 8 weeks with the allocation, evaluation time, and their interaction as fixed effects, the baseline value as a covariate, and the patient as a random effect. Using the REML (Restricted Maximum Likelihood) method for analysis, specify a compound symmetry for the covariance structure. The Kenward and Roger method is used to calculate the degrees of freedom. Estimate the treatment effect as the geometric mean ratio (ref. control group) based on the least-squared means at 8 weeks. Output the P value of the test against the null hypothesis where the ratio of the geometric means is 1 and determine the presence or absence of the treatment effect based on a significance level of 5%.

## Secondary endpoints

The following analysis will be performed for FAS:

1. Between-group ratio of the proportional change in geometric mean NT-proBNP at 4 weeks following protocol treatment initiation, compared with baseline.

Estimate the ratio of the geometric mean at 4 weeks (ref. control group) in the analysis (3) of primary endpoint.

1. Percentage of cases in which the NT-proBNP value decreased by 50% or more from the baseline, at 8 weeks following protocol treatment initiation.
2. Percentage of cases in which the NT-proBNP value decreased by 30% or more from the baseline, at 4 weeks following protocol treatment initiation.
3. Percentage of cases which mean NT-proBNP value decreased by 40% or more from the baseline at 4 and 8 weeks following protocol treatment initiation.

Frequency calculations will be performed for cases in which the NT-proBNP value decreased by 50%/30%/40% or more from the baseline, at 8 weeks/4 weeks/average of 4 and 8 weeks following the protocol treatment initiation. The percentages of cases that achieved a decrease of 50%/30%/40% or more in NT-proBNP at 8 weeks/ 4 weeks/average of 4 and 8 weeks were analyzed by logistic regression analyses adjusted for the baseline NT-proBNP values. Output the adjusted odds ratio (ref. control group), the two-sided 95% confidence interval using the Wald method, and the P value for testing against the null hypothesis with an odds ratio of 1.

1. Changes from the baseline of cardiac troponin T, CRP, GDF-15, soluble ST2, glycoalbumin, and 1.5AG at 8 weeks after following protocol treatment initiation.

Descriptive statistics will be calculated for each endpoint and the changes of each endpoint thereof for each treatment group and evaluation period. Regarding the mean or geometric mean and the change of means or proportional change of geometric means of each endpoint, the inferential statistics are calculated and plotted for each treatment group and evaluation period. Each endpoint at 8 weeks is analyzed using a linear model with the group as a fixed effect and the baseline value as a covariate, after which the difference or ratio between the treatment groups based on the least-squared means at 8 weeks (ref. control group) is estimated. Output the P value of the test against the null hypothesis with a difference or ratio between the treatment groups of 0 or 1. The necessity of logarithmic transformation of data is determined by evaluating the distribution shape of the data. No redundancy adjustment will be performed.

1. Changes from the baseline of weight, BMI, blood pressure, pulse rate, laboratory test values, and NYHA class at the 4 and 8 weeks following protocol treatment initiation.

Descriptive statistics will be calculated for each treatment group and evaluation period for each endpoint and the changes of each endpoint, other than the NYHA class. Regarding the mean or geometric mean and the change of means or proportional change of geometric means of each endpoint, the inferential statistics are calculated and plotted for each treatment group and evaluation period. For each endpoint at the 4 and 8 weeks, analyses of the mixed-effects model for repeated measurements (MMRM) is performed with the allocation, evaluation time, and their interaction as fixed effects, the baseline value as a covariate, and the patient as a random effect. Using the REML (Restricted Maximum Likelihood) method for analysis, specify a complex symmetric structure as the marginal covariance structure. The Kenward and Roger method is used to calculate the degrees of freedom. Estimate the difference/ratio between treatment groups (ref. control group) based on the least-squared means at 4 and 8 weeks. Output the P value of the test against the null hypothesis with a difference or ratio between the treatment groups of 0 or 1. The necessity of logarithmic transformation of data is determined by evaluating the distribution shape of the data. No redundancy adjustment will be performed.

Regarding NYHA classes, the frequency of each class and the frequency of change from the baseline (worsened, unchanged, improved) will be summarized for each treatment group and evaluation period, then plotted in graphs. The changes from the baseline at 4 and 8 weeks were analyzed by ordinal logistic regression with the fixed effects of allocation, evaluation time, and their interaction. The estimation is based generalized estimating equations that account for correlations in the longitudinal measurement data within patients. Output the common odds ratio for better category at 4 and 8 weeks (ref. control group) and the 95% confidence interval thereof, along with the P value of the test of the null hypothesis with an odds ratio of 1.

1. Changes from the baseline of cardiac function indicators (LVEDV, LVESV, LVEF, septal e', lateral e', mitral orifice blood flow velocity waveform (E), E/e', LVMI, LAVI , LVOT, LVOT-VTI, TR velocity, IVC, GLS, Left atrial strain (2-chamber view and 4-chamber view), at 8 weeks following protocol treatment initiation, along with the percentage of cases with a respiratory variation of 50% or more in IVC.

Descriptive statistics will be calculated for each treatment group and evaluation period for each endpoint and the changes of each endpoint. mean or geometric mean and the change of means or proportional change of geometric means of each endpoint, the inferential statistics are calculated and plotted for each treatment group and evaluation period. Each endpoint at 8 weeks is analyzed using a linear model with the group as a fixed effect and the baseline value as a covariate, after which the difference or ratio between the treatment groups based on the least-squared means at 8 weeks (ref. control group) is estimated. Output the P value of the test against the null hypothesis with a difference or ratio between the treatment groups of 0 or 1. The necessity of logarithmic transformation of data is determined by evaluating the distribution shape of the data. No redundancy adjustment will be performed.

Frequency calculations will be performed for cases with respiratory fluctuations of 50% or more in IVC. Additionally, regarding the percentage of cases that achieved a respiratory fluctuation of 50% or more in IVC, output the adjusted odds ratios (ref. control group) and two-sided 95% confidence intervals using the Wald method, along with the P value of the test against the null hypothesis with an adjusted odds ratio of 1, by logistic regression analysis adjusted using the baseline IVC values.

1. Changes from the baseline of KCCQ-12 at 8 weeks following protocol treatment initiation, along with the percentage of cases whose score increased by 5 points or more.

Descriptive statistics for KCCQ-12 and the changes thereof will be calculated for each treatment group and evaluation period. Inferential statistics will be calculated and plotted for each treatment group along with the evaluation period for the mean and mean change of each endpoint. Regarding KCCQ-12 at 8 weeks, the difference between treatment groups (ref. control group) based on the least-squared means at 8 weeks is calculated by analysis using a linear model with the group as a fixed effect and baseline value as a covariate.

Frequency calculations will be performed for cases in which the score increased by 5 points or more. Regarding the percentage of cases whose score increased by 5 points or more, output the adjusted odds ratios (ref. control group) and two-sided 95% confidence intervals using the Wald method, along with the P value of the test against the null hypothesis with an adjusted odds ratio of 1, by the logistic regression analysis adjusted using the baseline KCCQ-12 values.

1. Time to composite outcomes of first HF event [the following events due to the exacerbation of HF: i) unplanned rehospitalization ii) initiation of intravenous treatment for HF (vasodilators, inotropes) (during hospitalization: excluding rehospitalization); iii) emergency visit for HF requiring intravenous therapy (vasodilators, inotropes, diuretics); and iv) initiation of oral diuretics (loop diuretics, thiazide diuretics, tolvaptan) or a dose ;increase of ≥50% (outpatient)] and all-cause mortality.

Describe the number of occurrences for each treatment group. The cumulative incidence function will be described using the Kaplan-Meier method for each treatment group. Output the hazard ratio according to the Cox proportional hazards model (ref. control group) with the treatment group as a fixed effect, two-sided 95% confidence interval according to the Wald method, and P value of the test against the null hypothesis with a hazard ratio of 1.

1. Number and frequency of composite outcomes of compound events consisting of HF events including recurrence and all-cause mortality.

Describe the number of occurrences for each treatment group. Describe the mean cumulative function over time based on the Nelson-Aalen estimator for each treatment group. Using the recurrence event data analysis method proposed by Andersen-Gill (1982)^1^, output the hazard ratio for compound events consisting of HF events including recurrence and all-cause mortality (ref. control group), a two-sided 95% confidence interval based on robust variance, and a P value for the test against the null hypothesis with a hazard ratio of 1.

1. Number and frequency of following individual events: first and recurrent HF events, all-cause mortality, and cardiovascular death.

For all-cause mortality, the same analysis as in 9 will be performed. Although the same analysis as in 9 will be performed for the first HF event and the individual events thereof, analysis is performed using all-cause mortality as a competing risk according to Fine and Gray (1999)^2^. Although the same analysis as in 9 will be performed for cardiovascular death, analysis is performed using non-cardiovascular death as a competing risk according to Fine and Gray (1999)^2^. Although the same analysis as in 10 will be performed for individual recurrent HF events, analysis is performed using all-cause mortality as a competing risk according to Andersen et al. (2019)^3^. Although the same analysis as in 10 will be performed for compound events consisting of recurrent HF events and cardiovascular death, analysis is performed using death other than cardiovascular death as a competing risk according to Andersen et al. (2019)^3.^ If the number of occurrences of each event is small and a valid analysis cannot be performed, only the number of occurrences should be counted.

1. Number of occurrences and time to onset of specific adverse events, such as worsening of renal function (more than 50% increase in serum Cr or more than 30% decrease in eGFR), hyperkalemia (serum potassium 5.5 mEq/L or more), symptomatic hypotension, and angioedema.

For each treatment group, the number of occurrences of the above specific adverse events and total follow-up time will be tallied and the incidence rate and 95% confidence interval of the above specific adverse events calculated via Poisson regression analysis using robust variance.

1. Number of occurrences of other serious adverse events after the baseline.

Create a list of all adverse events and individual adverse events that occurred after the baseline. The list should include the patient ID, item name, date of onset, severity, presence or absence of causal relationship, outcome, and date of outcome. For individual adverse events, describe the number of occurrences and number of patients for each treatment group and calculate the incidence rate and 95% confidence interval using the Clopper-Pearson method.

## Subgroup analysis for primary endpoint

Regarding the primary endpoint, subgroup analysis will be conducted based on the following variables for FAS. If information related to subgroup definition is missing, the case will be excluded from the subgroup analysis.

- Demographic variables
- Age (at the time of screening period) (Over or under 65)
- Age (at the time of screening period) (Over or under 75)
- Sex (Male/Female)
- HF related
- History of HF (regardless of hospitalization history) (at the time of screening period)
- Have history of HF (more or less than 1.5 years) (regardless of hospitalization history) (at the time of screening period)
- HF hospitalization history (at the time of screening period)
- Causes of HF (ischemic, non-ischemic)
- NYHA (at the time of screening period) II/III/IV
- LVEF (at the time of screening period) (Over or under 60)
- LVEF (at the time of screening period) (less than 40, more than 40 but less than 60, more than 60)
- NT-proBNP (Day 0) (more or less than median)
- Test value
- BMI (at the time of screening period) (more or less than median)
- SBP (at the time of screening period) (more or less than median)
- eGFR (at the time of screening period) (Over or under 60)
- Comorbidities/pre-existing diseases
- Presence or absence of atrial fibrillation (at the time of screening period)
- History of hypertension (at the time of screening period)
- History of diabetes (at the time of screening period)
- History of dyslipidemia (at the time of screening period)
- Concomitant drugs
- Whether ACE inhibitors or ARBs were taken at the time of index hospitalization
- Whether beta-blockers were taken (at the time of screening period)
- Whether calcium channel blockers were taken (at the time of screening period)
- Whether MRA was taken (at the time of screening period)
- Whether diuretics were taken (at the time of screening period)
- Whether SGLT2 inhibitors were taken (at the time of screening period)
- Others
- Number of days from admission to the start of medication (more or less than median)

## Safety endpoints

The following analysis will be performed for SS: Reported adverse events will be assigned a Low Level Language (LLT) code using the MedDRA/J dictionary. The version of MedDRA/J used for analysis shall be the latest version at the time the database is locked in.

Adverse events (TEAEs) that occurred since protocol treatment initiation will be tabulated. Adverse events for which a causal relationship with sacubitril/valsartan is determined as "related" are considered side effects.

If an adverse event with the same preferred term (PT) occurs multiple times in one example, the number of occurrences is counted as the number of cases, while the number of examples is counted separately as one case.

For all adverse events and side effects, calculate the number of occurrences, number of cases that occurred, incidence rate, and 95% confidence interval, using the Clopper-Pearson method for each treatment group.

The number of occurrences, the number of cases that occurred, and the incidence rate will be calculated for each treatment group according to the System Organ Class (SOC) and PT classification.

# **Statistical analysis and tabulation software**

| OS | Microsoft Windows 10 |
| --- | --- |
| Statistical analysis software | SAS 9.4, R4.1.0 |
| Tabulation software | R and Microsoft Excel |

The versions of SAS and R used should also be clearly stated in the statistical analysis report.

# **References**

1. Andersen PK, Gill RD (1982) Cox’s regression model for counting processes: a large sample study. Ann Stat 10:1100–1120
2. Fine JP and Gray RJ (1999) A proportional hazards model for the subdistribution of a competing risk. JASA 94:496-509.
3. Andersen PK, Angst J, Ravn H. Modeling marginal features in studies of recurrent events in the presence of a terminal event. Lifetime Data Anal 2019; 25: 681-95.

# **Amendment**

First amendment created on Dec. 1, 2023

| (Before change) Creation date: Jun. 13, 2023 | (After change) Creation date: December 1, 2023 | Reasons for change |
| --- | --- | --- |
| Jun. 13, 2023 ver1.0 | Dec. 1, 2023 ver1.1 | For revision |
| Head of statistical analysis  From: Department of Medical Science, Graduate School of Medicine, Osaka Metropolitan University, Medical Statistics  Name: Takumi Imai | Head of statistical analysis  From: Clinical Research Division, Organization for Clinical Medicine Promotion  Name: Takumi Imai | For revision |

**Table S1. Administration of Sac/Val**

|  | Baseline | Week 4 | Week 8 |
| --- | --- | --- | --- |
| Study protocol completed | － | 183 | 176 |
| On treatment | 183 | 178 (97.3) | 169 (96.0) |
| Sacubitril/valsartan 12/13 mg twice daily | 1 (0.5) | 3 (1.6) | 4 (2.3) |
| Sacubitril/valsartan 24/26 mg twice daily | 178 (97.3) | 100 (54.6) | 78 (44.3) |
| Sacubitril/valsartan 36/39 mg twice daily | 0 | 1 (0.5) | 0 |
| Sacubitril/valsartan 49/51 mg twice daily | 4 (2.2) | 63 (34.4) | 63 (35.8) |
| Sacubitril/valsartan 97/103 mg twice daily | 0 | 11 (6.0) | 24 (13.6) |
| Stopped prematurely* | － | 5 (2.7) | 7 (4.0) |

Among the full analysis set population

Data are shown as n or n (%).

* Due to adverse events

**Table S2. Administration of study drug in the control group**

|  | Baseline | Week 4 | Week 8 |
| --- | --- | --- | --- |
| Study protocol completed | － | 193 | 190 |
| On treatment | 193 | 192 (99.5) | 189 (99.5) |
| Angiotensin converting enzyme inhibitor | 58 (30.1) | 58 (30.1) | 58 (30.5) |
| Enalapril | 57 (29.5) | 57 (29.5) | 57 (30.0) |
| 1.25 mg daily | 3 (1.6) | 1 (0.5) | 1 (0.5) |
| 2.5 mg daily | 29 (15.0) | 22 (11.4) | 22 (11.6) |
| 5 mg daily | 23 (11.9) | 28 (14.5) | 28 (14.7) |
| 10 mg daily | 2 (1.0) | 6 (3.1) | 6 (3.2) |
| Trandolapril | 1 (0.5) | 1 (0.5) | 1 (0.5) |
| 1 mg daily | 0 | 1 | 1 |
| 2 mg daily | 1 | 0 | 0 |
| Angiotensin receptor blocker | 135 (69.9) | 134 (69.4) | 131 (68.9) |
| Candesartan | 126 (65.3) | 125 (64.8) | 122 (64.2) |
| 2 mg daily | 18 (9.3) | 21 (10.9) | 21 (11.1) |
| 4 mg daily | 68 (35.2) | 45 (23.3) | 43 (22.6) |
| 6 mg daily | 0 | 4 (2.1) | 4 (2.1) |
| 8 mg daily | 38 (19.7) | 50 (25.9) | 49 (25.8) |
| 10 mg daily | 0 | 1 (0.5) | 1 (0.5) |
| 12 mg daily | 2 (1.0) | 4 (2.1) | 4 (2.1) |
| Azilsartan | 2 (1.0) | 2 (1.0) | 2 (1.1) |
| 2.5 mg daily | 1 (0.5) | 1 (0.5) | 1 (0.5) |
| 10 mg daily | 1 (0.5) | 1 (0.5) | 1 (0.5) |
| Olmesartan | 5 (2.6) | 5 (2.6) | 5 (2.6) |
| 20 mg daily | 5 | 5 | 5 |
| Telmisartan | 2 (1.0) | 2 (1.0) | 2 (1.1) |
| 20 mg daily | 1 (0.5) | 1 (0.5) | 1 (0.5) |
| 40 mg daily | 1 (0.5) | 1 (0.5) | 1 (0.5) |
| Stopped prematurely* | － | 1 (0.5) | 1 (0.5) |

Among the full analysis set population

Data are shown as n or n (%).

* Due to adverse events

**Table S3. Use of other heart failure medications at baseline and week 8**

| Medication | Baseline | | Week 8 | |
| --- | --- | --- | --- | --- |
|  | Sac/Val  (n = 183) | Control  (n = 193) | Sac/Val  (n = 176) | Control  (n = 190) |
| β-blocker | 142 (77.6) | 151 (78.2) | 147 (83.5) | 157 (82.6) |
| MRA | 145 (79.2) | 147 (76.2) | 131 (74.4) | 137 (72.1) |
| SGLT2 inhibitor | 114 (62.3) | 126 (65.3) | 104 (59.1) | 126 (66.3) |
| Loop diuretic | 142 (77.6) | 153 (79.3) | 121 (68.8) | 145 (76.3) |
| Thiazide | 2 (1.1) | 1 (0.5) | 2 (1.1) | 2 (1.1) |
| Daily dose, mg* | 20 (20, 20) | 20 (20, 40) | 20 (10, 20) | 20 (20, 30) |
| Tolvaptan | 36 (19.7) | 49 (25.4) | 28 (15.9) | 46 (24.2) |
| Digoxin | 2 (1.1) | 3 (1.6) | 2 (1.1) | 2 (1.1) |
| Ivabradine | 6 (3.3) | 2 (1.0) | 7 (4.0) | 4 (2.1) |
| Vericiguat | 1 (0.5) | 0 (0.0) | 4 (2.3) | 0 (0.0) |

Data are shown as n (%) or median (interquartile range).

* Furosemide-equivalent dose

MRA, mineralocorticoid receptor antagonist; SGLT2, sodium-glucose co-transporter 2.

**Table S4. Changes in clinical and laboratory data**

| Outcome and time | Sac/Val (n = 183*) | Control (n = 193^†^) | Group difference^‡^ | |
| --- | --- | --- | --- | --- |
|  | Mean (95% CI) | Mean (95% CI) | Mean (95% CI) | *P* value |
| BMI, kg/m^2^ |  |  |  |  |
| At baseline | 23.1 (22.5 to 23.7) | 23.5 (22.9 to 24.1) |  |  |
| At week 4 | 22.4 (21.8 to 23.0) | 23.0 (22.4 to 23.6) |  |  |
| At week 8 | 22.5 (21.9 to 23.1) | 23.0 (22.4 to 23.6) |  |  |
| Δ from baseline to week 4 | −0.7 (−0.9 to −0.5) | −0.6 (−0.8 to −0.4) | −0.2 (−0.5 to 0.1) | 0.140 |
| Δ from baseline to week 8 | −0.6 (−0.8 to −0.4) | −0.6 (−0.8 to −0.4) | −0.1 (−0.4 to 0.2) | 0.486 |
| SBP, mm Hg |  |  |  |  |
| At baseline | 120 (118 to 123) | 118 (116 to 121) |  |  |
| At week 4 | 116 (113 to 118) | 117 (115 to 120) |  |  |
| At week 8 | 117 (114 to 119) | 118 (115 to 120) |  |  |
| Δ from baseline to week 4 | −4.6 (−7.5 to −1.8) | −0.9 (−3.8 to 1.9) | −2.6 (−6.1 to 1.0) | 0.160 |
| Δ from baseline to week 8 | −3.4 (−6.3 to −0.5) | −0.3 (−3.2 to 2.5) | −1.9 (−5.5 to 1.7) | 0.310 |
| DBP, mm Hg |  |  |  |  |
| At baseline | 71 (69 to 73) | 71 (69 to 73) |  |  |
| At week 4 | 66 (65 to 68) | 67 (65 to 68) |  |  |
| At week 8 | 68 (66 to 70) | 68 (66 to 69) |  |  |
| Δ from baseline to week 4 | −4.9 (−7.0 to −2.7) | −4.4 (−6.5 to −2.3) | −0.3 (−2.6 to 2.1) | 0.822 |
| Δ from baseline to week 8 | −3.1 (−5.2 to −0.9) | −3.3 (−5.4 to −1.2) | 0.4 (−2.0 to 2.8) | 0.738 |
| Heart rate, beats per min |  |  |  |  |
| At baseline | 75 (73 to 77) | 74 (72 to 76) |  |  |
| At week 4 | 73 (71 to 75) | 74 (72 to 76) |  |  |
| At week 8 | 74 (72 to 76) | 74 (72 to 76) |  |  |
| Δ from baseline to week 4 | −1.7 (−4.4 to 1.0) | −0.6 (−3.2 to 2.0) | −0.5 (−3.5 to 2.4) | 0.716 |
| Δ from baseline to week 8 | −1.0 (−3.7 to 1.7) | −0.1 (−2.7 to 2.5) | −0.4 (−3.4 to 2.5) | 0.783 |
| eGFR, mL/min/1.73 m^2^ |  |  |  |  |
| At baseline | 51.6 (49.4 to 53.7) | 51.1 (49.0 to 53.3) |  |  |
| At week 4 | 49.2 (47.0 to 51.3) | 48.3 (46.2 to 50.4) |  |  |
| At week 8 | 50.9 (48.7 to 53.0) | 48.2 (46.1 to 50.3) |  |  |
| Δ from baseline to week 4 | −2.4 (−4.0 to −0.8) | −2.8 (−4.3 to −1.2) | 0.4 (−1.7 to 2.5) | 0.693 |
| Δ from baseline to week 8 | −0.7 (−2.2 to 0.9) | −2.9 (−4.5 to −1.4) | 2.3 (0.2 to 4.5) | 0.031 |
| Potassium, mEq/L |  |  |  |  |
| At baseline | 4.1 (4.1 to 4.2) | 4.1 (4.1 to 4.2) |  |  |
| At week 4 | 4.4 (4.3 to 4.5) | 4.5 (4.4 to 4.5) |  |  |
| At week 8 | 4.3 (4.2 to 4.4) | 4.4 (4.4 to 4.5) |  |  |
| Δ from baseline to week 4 | 0.2 (0.1 to 0.3) | 0.3 (0.3 to 0.4) | −0.1 (−0.2 to 0.01) | 0.086 |
| Δ from baseline to week 8 | 0.2 (0.1 to 0.3) | 0.3 (0.2 to 0.4) | −0.1 (−0.2 to −0.02) | 0.019 |

Estimated using a mixed-effects model for repeated measures.

* In the full analysis set, five patients were excluded from analysis of BMI owing to missing data.

^†^ In the full analysis set, two patients for BMI, one patient for heart rate, one patient for eGFR, and one patient for potassium levels were excluded from each analysis owing to missing data.

^‡^ Group difference (Sac/Val – Control), adjusted by baseline values.

BMI, body mass index; CI, confidence interval; DBP, diastolic blood pressure; eGFR, estimated glomerular filtration rate; Sac/Val, sacubitril/valsartan; SBP systolic blood pressure.

**Table S5. Achievement of prespecified NT-proBNP level reduction**

| Outcome | Sac/Val | | Control | | Odds ratio* (95% CI) | *P* value |
| --- | --- | --- | --- | --- | --- | --- |
|  | n | Achieved | n | Achieved |  |  |
| 50% reduction at week 8 | 176 | 84 (47.7%) | 189 | 61 (32.3%) | 2.09 (1.34 to 3.27) | 0.001 |
| 30% reduction at week 4 | 181 | 94 (51.9%) | 188 | 69 (36.7%) | 2.08 (1.34 to 3.21) | 0.001 |
| 40% time-averaged reduction through weeks 4 and 8 | 174 | 78 (44.8%) | 185 | 61 (33.0%) | 1.79 (1.15 to 2.79) | 0.010 |

* Adjusted by baseline levels of NT-proBNP concentrations

CI, confidence interval; NT-proBNP, N-terminal pro-B-type natriuretic peptide; Sac/Val, sacubitril/valsartan.

**Table S6. Category change from baseline in NYHA functional class**

| Time | Sac/Val | | | | Control | | | | OR (95% CI)* | *P* value |
| --- | --- | --- | --- | --- | --- | --- | --- | --- | --- | --- |
|  | n | Improved | Unchanged | Worsened | n | Improved | Unchanged | Worsened |  |  |
| Week 4 | 182 | 127 (69.8%) | 52 (28.6%) | 3 (1.6%) | 192 | 118 (61.5%) | 71 (37.0%) | 3 (1.6%) | 1.43 (0.93 to 2.20) | 0.099 |
| Week 8 | 176 | 124 (70.5%) | 50 (28.4%) | 2 (1.1%) | 190 | 117 (61.6%) | 69 (36.3%) | 4 (2.1%) | 1.50 (0.97 to 2.31) | 0.070 |

* Common OR toward a better category

CI, confidence interval; NYHA, New York Heart Association; OR, odds ratio; Sac/Val, sacubitril/valsartan.

**Table S7. Changes in KCCQ-12 scores**

| Domains and time | Sac/Val | | Control | | Group difference* | |
| --- | --- | --- | --- | --- | --- | --- |
|  | n | Mean (95% CI) | n | Mean (95% CI) | Mean (95% CI) | *P* value |
| Summary score |  |  |  |  |  |  |
| At baseline | 151 | 47.6 (43.7 to 51.5) | 161 | 47.6 (43.8 to 51.3) |  |  |
| At week 8 | 141 | 76.2 (72.2 to 80.2) | 152 | 79.2 (75.4 to 83.1) |  |  |
| Δ from baseline to week 8 | 137 | 28.7 (23.9 to 33.5) | 147 | 31.5 (26.9 to 36.1) | −2.4 (−7.5 to 2.6) | 0.347 |
| Physical limitations |  |  |  |  |  |  |
| At baseline | 151 | 59.4 (54.5 to 64.3) | 161 | 59.5 (54.8 to 64.3) |  |  |
| At week 8 | 141 | 82.8 (77.7 to 87.8) | 154 | 83.5 (78.7 to 88.3) |  |  |
| Δ from baseline to week 8 | 137 | 23.7 (17.7 to 29.6) | 149 | 23.4 (17.6 to 29.1) | 0.2 (−5.7 to 6.0) | 0.952 |
| Symptom frequency |  |  |  |  |  |  |
| At baseline | 151 | 41.5 (37.2 to 45.9) | 161 | 43.3 (39.1 to 47.4) |  |  |
| At week 8 | 141 | 79.8 (75.4 to 84.3) | 153 | 83.3 (79.0 to 87.6) |  |  |
| Δ from baseline to week 8 | 137 | 38.2 (32.7 to 43.8) | 148 | 40.2 (34.8 to 45.5) | −2.4 (−7.8 to 3.0) | 0.388 |
| Quality of life |  |  |  |  |  |  |
| At baseline | 151 | 30.2 (26.3 to 34.2) | 161 | 29.3 (25.5 to 33.2) |  |  |
| At week 8 | 141 | 61.2 (57.1 to 65.3) | 154 | 63.6 (59.6 to 67.5) |  |  |
| Δ from baseline to week 8 | 137 | 31.2 (25.9 to 36.5) | 149 | 34.6 (29.6 to 39.7) | −2.7 (−8.7 to 3.4) | 0.390 |
| Social limitations |  |  |  |  |  |  |
| At baseline | 151 | 59.3 (53.6 to 65.0) | 161 | 58.2 (52.6 to 63.7) |  |  |
| At week 8 | 141 | 81.1 (75.2 to 86.9) | 153 | 85.6 (79.9 to 91.2) |  |  |
| Δ from baseline to week 8 | 137 | 21.7 (14.7 to 28.8) | 148 | 26.7 (19.9 to 33.5) | −4.0 (−11.2 to 3.2) | 0.275 |

* Group difference (Sac/Val – Control), adjusted by baseline values

CI, confidence interval; KCCQ, Kansas City Cardiomyopathy Questionnaire; Sac/Val, sacubitril/valsartan.

**Table S8. Percentage of patients who experienced improvement of KCCQ-12 scores (≥ 5 points) at week 8**

| Domains | Sac/Val | | Control | | OR (95% CI)* | *P* value |
| --- | --- | --- | --- | --- | --- | --- |
|  | n | ≥ 5-point increase | n | ≥ 5-point increase |  |  |
| Summary score | 137 | 109 (79.6%) | 147 | 117 (79.6%) | 0.81 (0.54 to 1.22) | 0.317 |
| Physical limitations | 137 | 100 (73.0%) | 149 | 101 (67.8%) | 1.09 (0.73 to 1.62) | 0.685 |
| Symptom frequency | 137 | 114 (83.2%) | 148 | 119 (80.4%) | 0.86 (0.57 to 1.28) | 0.446 |
| Quality of life | 137 | 109 (79.6%) | 149 | 116 (77.9%) | 0.84 (0.56 to 1.28) | 0.423 |
| Social limitations | 137 | 88 (64.2%) | 148 | 100 (67.6%) | 0.81 (0.54 to 1.21) | 0.306 |

* Sac/Val vs. Control, adjusted by baseline scores

CI, confidence interval; KCCQ, Kansas City Cardiomyopathy Questionnaire; OR, odds ratio; Sac/Val, sacubitril/valsartan.

**Table S9. Serious adverse events developed and reported**

| Event | Sac/Val | Control |
| --- | --- | --- |
| Overall | 29 | 30 |
| Angina | 0 | 2 |
| Aortic valve replacement | 1 | 0 |
| Bacteremia | 0 | 1 |
| Cardiac resynchronization therapy defibrillator | 1 | 0 |
| Cardiopulmonary arrest | 1 | 0 |
| Catheter ablation | 1 | 0 |
| Cholecystitis | 1 | 0 |
| Colon polyp | 0 | 1 |
| Coronary revascularization | 4 | 3 |
| Coronavirus disease-2019 infection | 0 | 1 |
| Dehydration | 1 | 1 |
| Diverticulitis | 0 | 1 |
| Electrical defibrillation | 0 | 1 |
| Epididymitis | 1 | 0 |
| Fracture | 0 | 1 |
| Hypokalemia | 0 | 1 |
| Hyponatremia | 0 | 1 |
| Ileus | 1 | 0 |
| Ischemic enteritis | 0 | 1 |
| Myocardial infarction | 2 | 0 |
| Pseudoaneurysm | 0 | 1 |
| Pyogenic spondylitis | 0 | 1 |
| Recurrent lymphoma | 0 | 1 |
| Stroke | 1 | 1 |
| Sudden death | 0 | 1 |
| Urinary tract infarction | 1 | 0 |
| Urine hyperosmolality | 0 | 1 |
| Ventricular fibrillation | 1 | 1 |
| Worsening heart failure | 9 | 6 |
| Worsening renal function | 3 | 2 |

Among an intention-to-treat-based safety analysis set (n = 394; 195 for the Sac/Val group and 199 for the control group).

Data are total number of serious adverse events developed and reported.

Sac/Val, sacubitril/valsartan.

**Figure S1. Individual plotting of changes in NT-proBNP level**


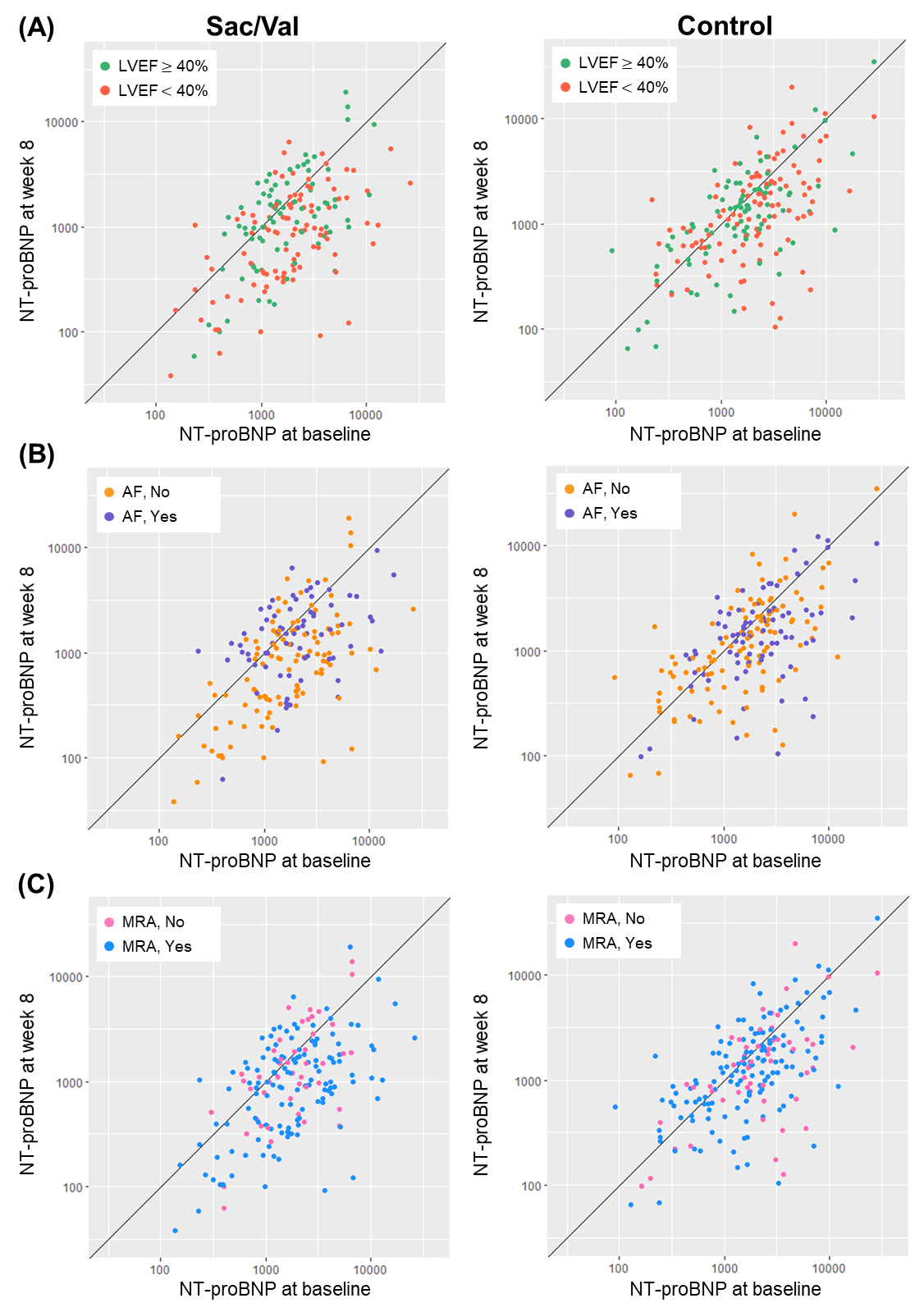


The points on the diagonal indicate that NT-proBNP values did not change over 8 weeks. Points below the diagonal indicate a decrease in NT-proBNP values, while points above the diagonal indicate an increase in NT-proBNP values. The plots are stratified by (A) LVEF status (< 40% or ≥ 40%), (B) AF (Yes or No), and (C) taking MRA (Yes or No) in each treatment group.

AF, atrial fibrillation; LVEF, left ventricular ejection fraction; MRA, mineralocorticoid receptor antagonist; NT-proBNP, N-terminal pro-B-type natriuretic peptide; Sac/Val, sacubitril/valsartan.

**Figure S2. Change in NYHA functional class over 8 weeks**

**
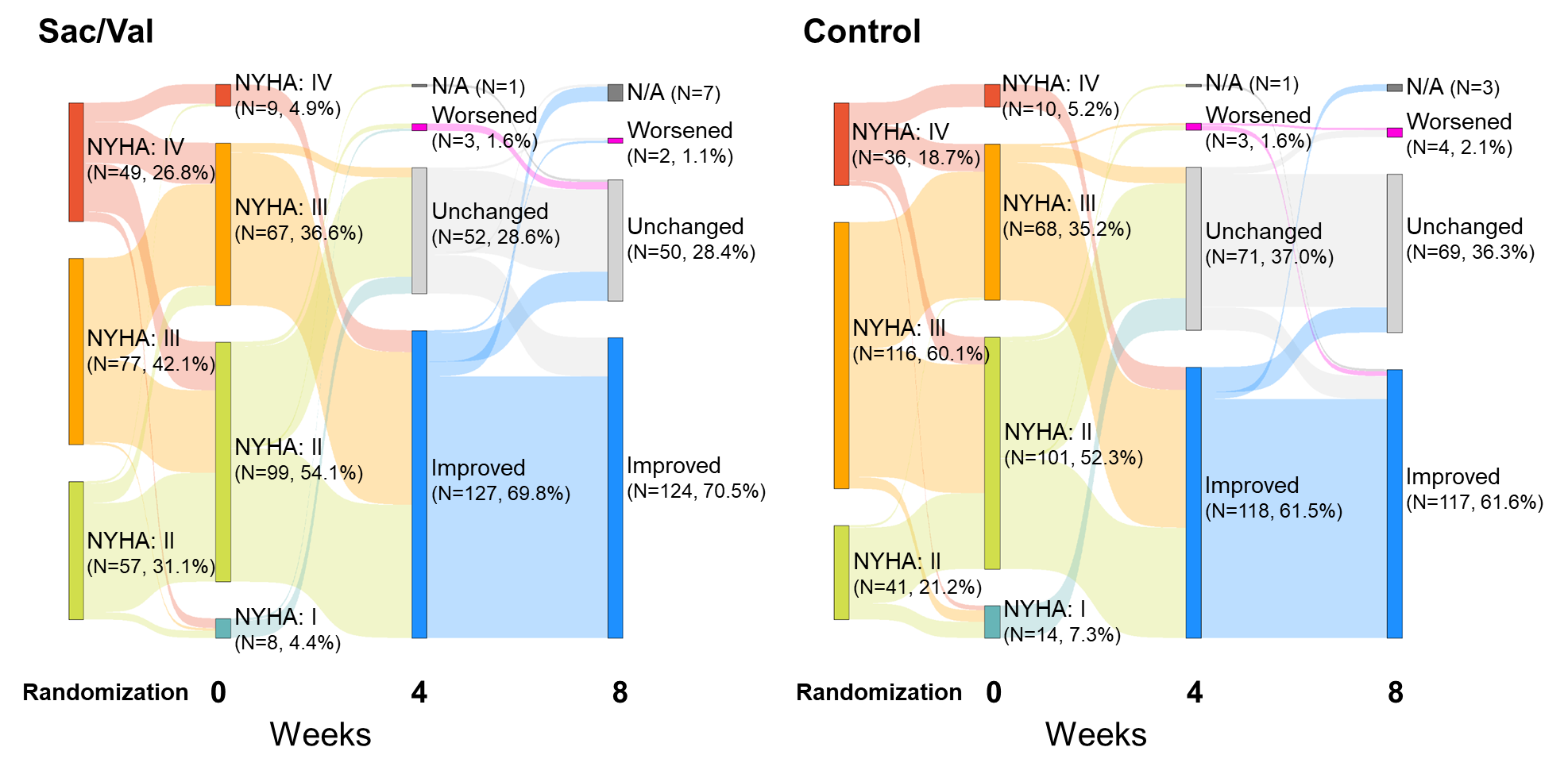
**

The figure shows the proportion of patients whose NYHA classes were improved, unchanged, or worsened at weeks 4 and 8, as well as their trajectories.

Sac/Val, sacubitril/valsartan; NYHA, New York Heart Association.

**Figure S3. Effect of Sac/Val therapy on prespecified clinical events**


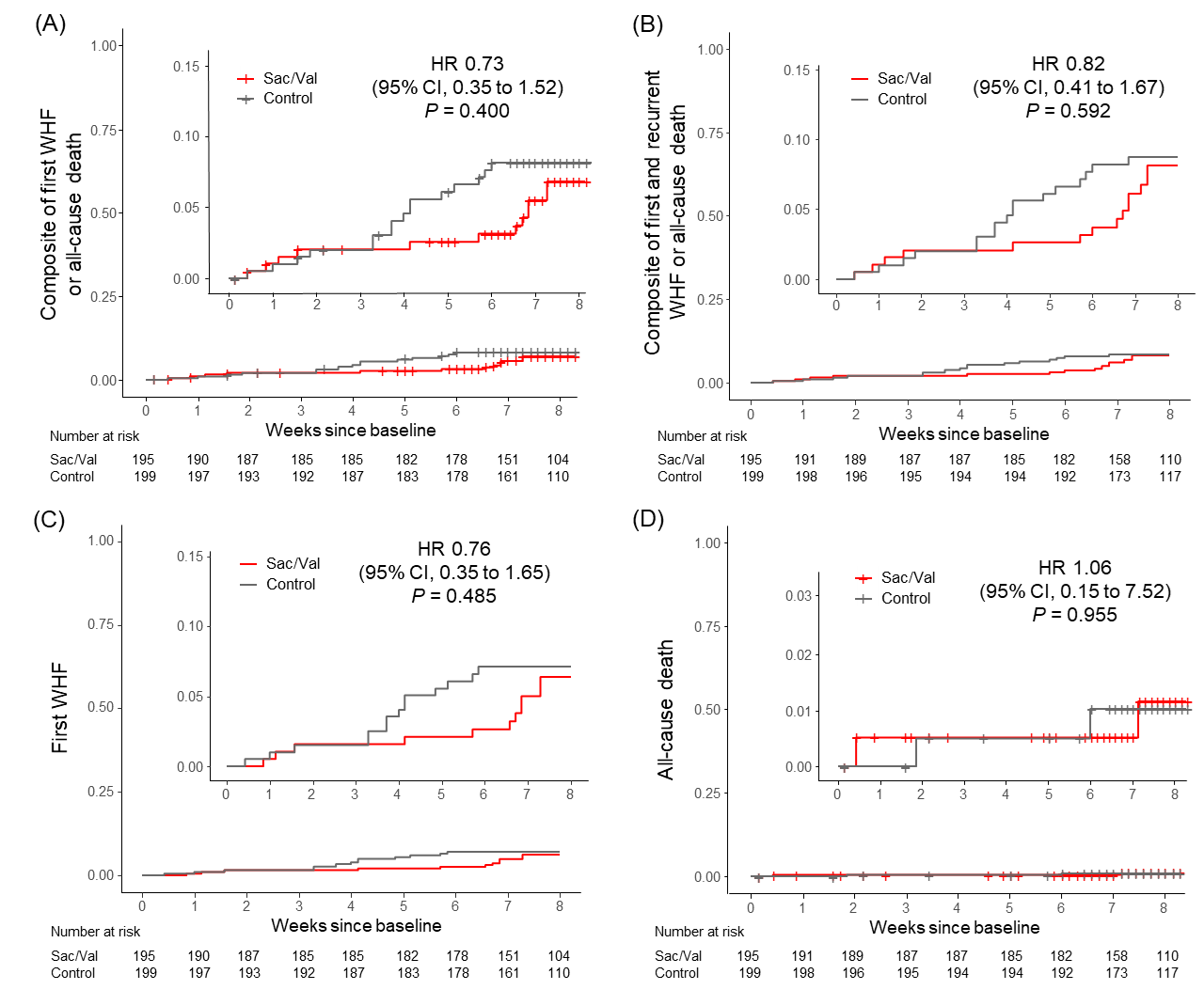


Estimated cumulative incidence function of the composite of first WHF* or all-cause death (A), mean cumulative function of the composite of first and recurrent WHF* or all-cause death (B), cumulative incidence function of the first WHF* (C), and cumulative incidence function of all-cause death (D).

* Defined as (i) unplanned rehospitalization; (ii) initiation of intravenous treatment (vasodilator or inotropic agent) for heart failure during index hospitalization, excluding at rehospitalization; (iii) urgent visit due to heart failure requiring intravenous treatment (vasodilator, inotropic agent, or diuretic); or (iv) initiation of oral diuretic (loop diuretic, thiazide-type diuretic, or tolvaptan) or at least a 50% increase in its dose (outpatient).

CI, confidence interval; HR, hazard ratio; Sac/Val, sacubitril/valsartan; WHF, worsening heart failure.
